# HFPO-DA and Other PFAS in Air Downwind of a Fluoropolymer Production Plant in the Netherlands: Measurements and Modeling

**DOI:** 10.1021/acs.est.4c13943

**Published:** 2025-04-21

**Authors:** Joost Dalmijn, Julia J. Shafer, Jonathan P. Benskin, Matthew E. Salter, Jana H. Johansson, Ian T. Cousins

**Affiliations:** † Department of Environmental Science, Stockholm University, SE-10691 Stockholm, Sweden; ‡ Department of Thematic Studies—Environmental Change, Linköping University, 581 83 Linköping, Sweden

**Keywords:** GenX, FRD-902, 6:2 FTSA, emulsifier, processing aid, polymerization byproducts, emission abatement, atmospheric dispersion, particulates, FLEXPART, particle phase, aerosols

## Abstract

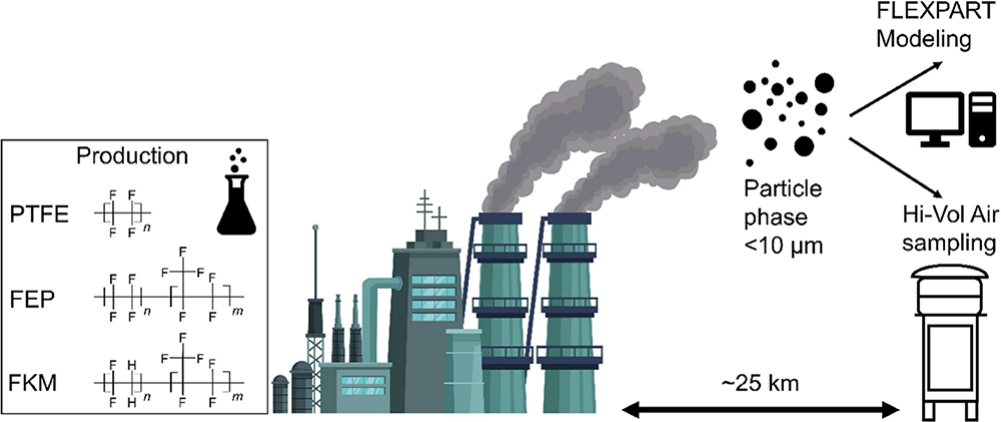

Emissions of historical fluorinated processing aids used in fluoropolymer production are known to have contributed significantly to environmental levels of persistent perfluoroalkyl acids (PFAAs). Less is known about emissions of contemporary processing aids and the efficacy of technology used to contain them. To address this, we investigated the occurrence of hexafluoropropylene oxide dimer acid (HFPO-DA) and other per- and polyfluoroalkyl substances (PFAS) in airborne PM10 near a fluoropolymer production plant in the Netherlands. The 20-week high-volume air sampling campaign coincided with installation of emission abatement systems. HFPO-DA levels ranged from below detection limits to 98.66 pg m^–3^ when the wind came from the plant, and decreased to a maximum of 12.21 pg m^–3^ postabatement. Lagrangian dispersion modeling using FLEXPART revealed good concordance between measured and modeled HFPO-DA concentrations (Pearson’s *r* = 0.83, *p* ≤ 0.05, Wilmott’s *d* = 0.71, mean absolute error = 3.66 pg m^–3^), providing further evidence that the plant is a point source. Modeling also suggested that HFPO-DA could undergo long-range atmospheric transport with detectable HFPO-DA air concentrations predicted up to several thousand kilometers away. Besides HFPO-DA, the fluorinated processing aid 6:2 fluorotelomer sulfonate and the suspected polymerization byproducts, hydrogen-substituted perfluoroalkyl carboxylic acids, were also observed, highlighting the complex mixture of PFAS emitted by the plant.

## Abstract 2

This study assesses atmospheric PFAS emissions from a fluoropolymer production plant and evaluates emission abatement effectiveness using measurements and atmospheric dispersion modeling.

## 1 Introduction

Fluoropolymer production plants (FPPs) are known point sources of per- and polyfluoroalkyl substances (PFAS) to the environment.^1^ The most studied emissions of FPPs involve fluorinated processing aids.^2^ These stable and strong surfactants have been used for emulsion polymerization of certain fluoropolymers (e.g., polytetrafluoroethylene (PTFE)) since the 1950s^3^ and are designed to emulsify and stabilize aqueous polymerization dispersions, while minimizing interference with the polymerization reaction.^4^ Processing aids are by definition not part of the end-product and emissions typically occur during processing steps after polymerization, when the polymer dispersion is washed or dried.^5^ Historically, salts of perfluoroalkyl carboxylic acids (PFCAs), such as perfluorooctanoic and perfluorononanoic acid (PFOA and PFNA, respectively) were the most common fluoropolymer processing aids.^3^ However, due to their persistence, toxicity and bioaccumulation potential, these substances have become increasingly regulated, with PFOA added to Annex A of the Stockholm Convention on Persistent Organic Pollutants in 2019,^6,7^ and long-chain PFCAs (including PFNA) currently under review.^8^ In addition to fluorinated processing aids, other PFAS are also used, formed, or emitted during fluoropolymer production. These include monomers, polymerization and monomer production byproducts, chain transfer and curing agents, and fluorinated solvents.^9^

Since the start of the phase-out of long-chain PFCAs from fluoropolymer production in 2002, industry has introduced various replacement processing aids.^10^ Most of these substances are ammonium salts of perfluoroalkylether carboxylic acids (PFECAs) which contain fewer perfluoroalkyl moieties than long-chain PFCAs.^11^ However, like the processing aids they replaced, these substances are PFAS according to the OECD definition.^12^ The ammonium salt of hexafluoropropylene oxide dimer acid (HFPO-DA, “GenX”), is a replacement that was first introduced around 2009 by Chemours’ predecessor DuPont as a processing aid in their emulsion polymerization portfolio.^13^

Due to their high acid dissociation constants (*K*_a_), PFECAs occur predominantly in their anionic forms in the environment.^14−16^ The inclusion of ether linkages in their tail structure enhances surfactant strength without additional perfluoroalkyl moieties, while also increasing solubility compared to their long-chain predecessors.^17^ Despite the lower bioaccumulation potential of replacements,^18^ concerns about the persistence and toxicity of PFECAs remain.^19,20^ Moreover, due to their higher water solubility, these replacements are more mobile in soils and water, hampering treatment technologies aimed at removing PFAS based on sorption (e.g., to activated carbon).^21,22^ This has led to the classification of HFPO-DA as a Substance of Very High Concern (SVHC) by the European Chemicals Agency (ECHA) in 2019.^23^

After release into the atmosphere, perfluoroalkyl (ether) carboxylic acids (PF(E)CAs) are thought to mostly partition to the accumulation mode of the particle phase.^24−26^ Consequently, a portion of FPP emissions may travel long distances from the source, potentially contaminating remote regions.^27^ High ground-level air concentrations of particle-bound pollutants are generally associated with strong winds from the direction of a point source, as turbulent airflows caused by wind-surface interactions bring the buoyant plume from the stack down to ground level.^28^ Therefore, sampling and analysis of PFAS in particulates provides valuable insights into air emissions of these substances from such sources. It should be noted that the pH of atmospheric particles has a broad range (in Europe: 2.6–6.7, average = 3.9)^29^ and a range of different p*K*_a_ values for PF(E)CAs has been reported (HFPO-DA: −0.77 to 2.84).^30,11^ Therefore, partial partitioning of HFPO-DA to the gas phase to at least a minor extent cannot be ruled out.^31^

Most research to date has focused on contamination of surface water around FPPs.^32^ For example, concentrations of HFPO-DA up to 812 ng L^–1^ were reported downstream of the Dordrecht FPP.^33^ Similarly, this processing aid was detected in surface water near a Chemours HFPO-DA production site in Fayetteville, North Carolina (US) (631 ng L^–1^) and other sites where it is applied or produced, e.g., in the Ohio River near Washington, West Virginia (US) (10 ng L^–1^) and in the Xiaoqing river near FPPs in China (∼9000 ng L^–1^).^34−36^

Fewer data are available on HFPO-DA and other PFAS emissions from fluoropolymer production to air. A study published in 2006 by Barton et al. using cascade impactors at the fence line of an FPP near Washington, West Virginia (US), reported PFOA concentrations in the μg m^–3^ range.^37^ Similarly, a study conducted in 2007 using high-volume air sampling with glass fiber filters revealed peak PFOA concentrations of 828 pg m^–3^ at the Hazelrigg atmospheric monitoring station, 20 km northwest of a FPP in Thornton-Cleveleys (UK).^38^ Similar concentrations have been reported in more recent studies near FPPs in China,^39,40^ while a recent study in the US found PFAS levels in the low pg m^–3^ range on PM2.5 particulates.^41^ Investigations in the US and the Netherlands have indicated that HFPO-DA is subject to atmospheric transport and deposition, with the FPPs acting as a point source.^42,36^

Starting in the 2010s, regulatory pressure led to the introduction of additional emission abatement measures at various FPPs in Europe and the US that could reduce air emissions of certain PFAS by up to 99%.^43−45^ While these measures could contribute to lowering overall PFAS concentrations in these regions, their efficacy has never been investigated or reported in the peer-reviewed literature. Moreover, most published data on atmospheric emissions from FPPs have focused on a limited suite of known PFAS monitored using low-resolution mass spectrometry close to FPPs or through short-term sampling (i.e., over a few days).^38,37,39,26,41^ Clearly, a more comprehensive approach is needed to fully understand the extent of emissions from these facilities, along with the impact of potential abatement systems.

The aim of this study was to investigate the occurrence of the fluoropolymer processing aid HFPO-DA and other suspect and target PFAS at a site 25 km downwind of the Dordrecht FPP, before-, during- and after the installation of abatement systems. These data, together with measurements of wind speed and direction, were collected to provide a comprehensive picture of PFAS emissions from the plant and determine for the first time the efficacy of added abatement technology. Additionally, through the measurements at a distance of 25 km from the source the regional transport of emissions was investigated. Atmospheric dispersion modeling of emissions was used to further validate the measurements and to assess the local and long-range impacts of HFPO-DA emissions by this plant.

## 2 Materials and Methods

### 2.1 Study Site

The current study investigated the Chemours’ Dordrecht Works FPP. This plant has a total fluoropolymer production capacity of around 19,000 t per year and produces PTFE (Teflon), fluorinated ethylene propylene (Teflon FEP) and fluoroelastomers (Viton FKM).^46^ The site also produces the feedstock substance chlorodifluoromethane (HCFC-22) which is used for producing the monomers tetrafluoroethylene (TFE) and hexafluoropropylene (HFP) via pyrolysis. The ammonium salt of HFPO-DA is used here as a processing aid for the production of PTFE and FEP through emulsion polymerization, entirely replacing the ammonium salt of PFOA (ammonium perfluorooctanoate; APFO) in 2012. Additionally, the fluoropolymer processing aid Capstone FS10 (6:2 fluorotelomer sulfonate; 6:2 FTSA) is used at the site as a processing aid for the production of FKM.^47^

Air emissions of the Dordrecht FPP are regulated by and reported to the local authority Dienst Centraal Milieubeheer Rijnmond (DCMR) and the Province of South Holland. HFPO-DA is predominantly released to the air by the plant from the drying of polymer dispersions in a process similar to thermal desorption. It is unknown if HFPO-DA is released from the stacks as its ammonium salt, in dissociated form, as a neutral acid in the gas phase or a combination of these forms. Additionally, and as opposed to other PF(E)CAs, a considerable proportion of the HFPO-DA is thermally decarboxylated during this process and leaves the stack of the plant as fluoroether E1.^46^ Limits on the emissions of HFPO-DA to air from the plant were reduced from 660 (2012–2016) to 450 kg yr^–1^ in 2017, 95 kg yr^–1^ in 2020 and 2.3 kg yr^–1^ (20 kg yr^–1^ of E1) in 2021.^48,46^ Additionally, the current environmental permit states that small amounts (i.e., around 1 kg yr^–1^) of C4–C18 PFCAs and around 3 kg yr^–1^ of 6:2 FTSA are emitted to air by the plant under normal operating circumstances. In order to reach these emission levels, Chemours installed an abatement system (“Sequoia”), which is based on activated carbon scrubbers and connected the stacks of their PTFE and FEP production lines in Dordrecht to this system. The Sequoia system was first tested on a pilot scale starting in 2020 and fully connected during the summer of 2021. Fine tuning of the operational parameters of the system was concluded in the spring of 2022. Spent activated carbon from the abatement system is incinerated off-site.

### 2.2 Standards and Reagents

Standards for a total of 50 native PFAS (including HFPO-DA) as well as a suite of ^13^C isotope-labeled internal standards (ISs) were obtained from Wellington Laboratories (Guelph, ON, CA) and Apollo Scientific (Bredbury, UK). A full list of native standards, ISs and recovery standards (RSs) is provided in Table S1 of the Supporting Information-1 (SI-1)).

### 2.3 Sampling

**Figure 1:**
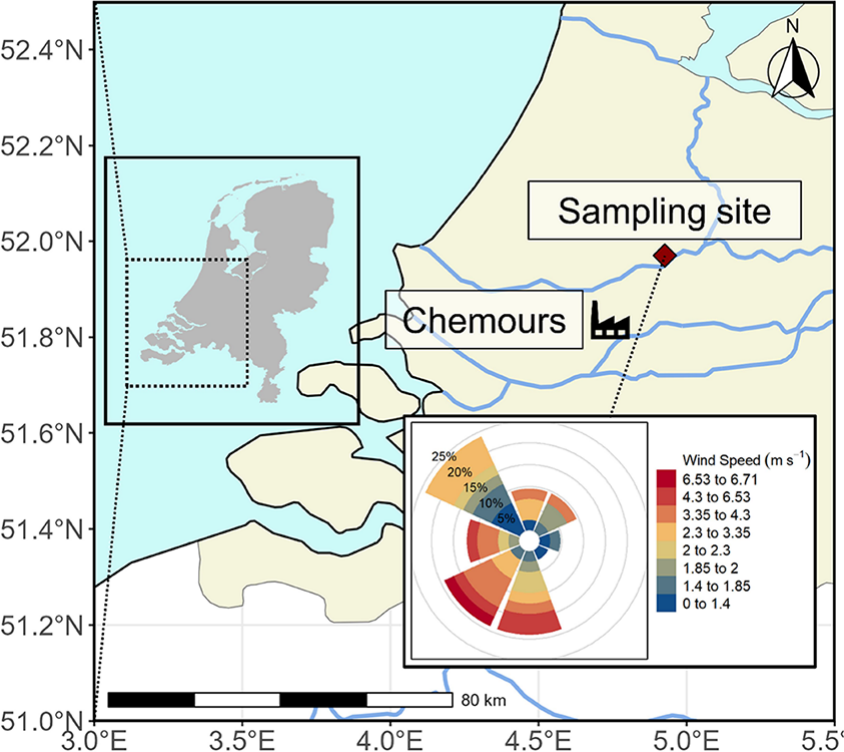
Map of the study area showing the location of the air sampling site at the Cabauw Meteorological Observatory and Chemours plant (red diamond), as well as the wind rose showing wind speeds and directions at the sampling site during the sampling campaigns.

Sampling campaigns were carried out in the summer and fall of 2021 in the Netherlands at the Cabauw meteorological observatory (located at 51.971° N, 4.927° E). This site was located about 25 km northeast of the FPP, which is downwind under prevailing meteorological conditions (Figure 1). Hourly wind direction and wind speed data were collected at this site and downloaded from the Dutch Royal Meteorological Institute (KNMI) website.^49^ Average wind speed and direction over the sampling periods were calculated using the formulas given in eqs 1–4 in SI-1. Additionally, meteorological parameters including relative humidity, precipitation, temperature and radiation were also collected or calculated from the hourly data (Table S10 and Figure S10).^49^

Airborne particulates were collected on 203 × 254 mm QM-A quartz fiber filters (QFFs) (VWR, Radnor, PA, USA), using a high-volume air sampler (Sierra Instruments, USA) equipped with a PM10 inlet at a height of 2 m above the ground and using a flow rate of 67 m^3^/h. QFFs were baked at 800 °C and individually packaged in aluminum foil prior to use. A common sampling artifact associated with this method is that gas-phase compounds partly sorb to the QFFs. As such, the resulting data does not necessarily reflect substances occurring exclusively in the particulate phase, and therefore it should not be used to assess gas-particle partitioning.^50^

After sampling, the QFFs were folded inward and packed in aluminum foil and individual zip-lock bags and subsequently stored in a freezer at −20 °C. Sampling volumes were around 1600 m^3^ for 24 h of sampling. In the first sampling campaign, multiple 24-h air samples were taken over a period of 3 weeks in June 2021. Subsequently, a second sampling campaign was carried out, involving weekly samples which were collected from July to October 2021. In October 2021, several additional daily samples were taken. Unfortunately, sampling was briefly interrupted in September 2021 due to a power failure.

### 2.4 Extraction

Prior to extraction, the QFFs were spiked with 2000 pg of a ^13^C-labeled IS PFAS mixture (Table S1) and then cut in half using solvent cleaned scissors. Punches of all QFFs were taken for the analysis of sea spray aerosol tracer ions using previously described methodology.^51^ The two portions of the QFF were extracted separately (due to their size), and the final extracts recombined prior to instrumental analysis. The extraction method was previously described in Sha et al.^51^ Briefly, QFFs were sonicated in 20 mL of methanol (MeOH, Honeywell, UPLC-grade) for 20 min in a 50 mL centrifuge tube. The MeOH was subsequently transferred to a clean tube and the procedure repeated three times. Thereafter, the extracts (including from the duplicate portion of QFF) were combined and reduced to dryness using a Biotage TurboVap. The samples were then reconstituted in 130 μL of methanol and sonicated for 15 min. Thereafter, 150 μL of Milli-Q (Merck, Darmstadt, DE) ultrapure water with 4 mM ammonium acetate (NH_4_CH_3_CO_2_) was added and the extracts were transferred to an Eppendorf tube with a 0.2 μm nylon centrifuge filter. The extract was centrifuged for 10 min at 12,000 rpm (16.1 × 10^3^ g) and transferred to a 300 μL PP LC-MS vial. Twenty μL of 20 pg/μL (400 pg) M8 PFOA and M8 PFOS RS was added and the samples were stored in a fridge at 4 °C until analysis.

### 2.5 UHPLC-HRMS Analysis

Instrumental analysis was conducted using a Dionex Ultimate 3000 ultrahigh-performance liquid chromatograph (UHPLC) linked to a Q-Exactive HF Orbitrap (Thermo Fisher Scientific, Waltham, MA, USA) with an electrospray ionization source (ESI) operated in negative mode. The UHPLC was equipped with a 1.7 μm, 50 × 2.1 mm Waters Acquity BEH C18 column and a Waters Acquity BEH C18 guard column (Waters, Wilmslow, UK), both maintained at 50 °C. The mobile phase, consisting of A: H_2_O:ACN (95:5) and B: ACN:H_2_O (95:5), both with 2 mM ammonium bicarbonate (NH_4_HCO_3_), was passed through a Waters PFC Isolator column (Waters, Wilmslow, UK). A 25 μL injection volume was used, and the LC gradient program details are available in Table S2. The Orbitrap was operated in full scan (scan range: 150–1800 Da, resolution: 120,000 fwhm)–data-dependent MS^2^ (resolution: 15,000 fwhm) mode, employing an inclusion list generated from approximately 5000 potential PFAS masses based on homologous series of PF(E)CAs and perfluoroalkyl (ether) sulfonic acids (PF(E)SAs) with various moieties and substitutions. Further details on HRMS settings can be found in Table S2 of the SI.

### 2.6 Data Handling

Data were processed using Thermo Fisher TraceFinder version 4.1 software. Individual PFAS were quantified using their exact mass (with 5 ppm mass accuracy tolerance) and retention times with an 8-point, relative response-based calibration curve (0.03 to 135 pg μL^–1^, with 15 pg μL^–1^ of IS in every standard). Additionally, full-scan and acquired MS^2^ spectra of the target analytes in both the calibration standard and the sample were compared for confirmation. Due to the unavailability of labeled standards for all PFAS included in our analysis, surrogate ISs (i.e., not exactly matched) were employed for quantification of some substances (Table S1). IS recoveries were calculated in relation to the RS (M8 PFOA or M8 PFOS; Table S9). Final concentrations in air samples were calculated by dividing the total mass of the detected analyte (pg) by the total volume of air sampled (m^3^).

The suspect screening method also used Thermo Fisher TraceFinder version 4.1 software in conjunction with Thermo Fisher Xcalibur Qual Browser. The initial list of suspects was based on matches (within 5 ppm mass error) to the in-house database used as an inclusion list during instrumental analysis. Thereafter, suspect peaks were inspected manually for MS^2^ and in-source fragmentation patterns, isotopic ratios, and retention times, which were compared to available literature. Further, for suspect matches with an acquired MS^2^ spectrum, in-silico predictions and fragment library matching were performed using Sirius5^52^ software and the MetFrag online fragmentation tool.^53^ In order to perform the in-silico predictions, .raw files were converted to.mzml files using Proteowizard MSConvert version 3.0.24081. Finally, to confirm putative identifications, authentic standards were procured and reanalyzed using the original instrumental analysis conditions. Features with a Schymanski confidence level of 1 or 2 or that were part of a homologous series of multiple suspects were included in the results and discussion.^54^

### 2.7 Atmospheric Dispersion Modeling

The FLEXPART Lagrangian atmospheric dispersion model was used to simulate HFPO-DA air concentrations at Cabauw near the Chemours’ facility to validate measurements.^55^ Two types of simulations were performed with the model, each requiring modifications to the COMMAND, OUTGRID, and RELEASE files: a local simulation to validate measurements and a regional-scale simulation to assess long-range transport. The local simulation covered the 5-month sampling campaign period (2021-06-01 to 2021-10-31) and was conducted in forward mode. A spatial domain between 4.4269°–5.0269° longitude and 51.515°–52.115° latitude was defined, with four vertical layers at 100, 500, 1000, and 50,000 m, using a spatial resolution of 0.002°. HFPO-DA was released at a height of 25.9 m, approximating the stack height of the processing plant, and boundary layer turbulence was calculated using the Gaussian model.

The distribution of HFPO-DA on aerosols was modeled using two log-normal modes based on size-resolved measurements by Lin et al.^26^ with each mode represented by a separate species file. The smaller mode had a mean diameter of 1.2 μm, a geometric standard deviation of 3 and contained 19% of the total mass, while the larger mode had a mean diameter of 12 μm, a geometric standard deviation of 1.6, and contained 81% of the total mass.

Due to uncertainties in emission rates, two scenarios based on environmental permits were evaluated: 95 and 3.2 kg yr^–1^.^46,43^ The evaluations suggested a substantial decrease in HFPO-DA emissions after June 22, leading to the construction of a mixed scenario using 95 kg yr^–1^ for most of June and 3.2 kg yr^–1^ for the remainder of the period (July to October 2021).

The regional-scale simulation covered a one-year period (January 1 to December 31, 2020) and was conducted in forward mode to assess long-range atmospheric transport under unabated emissions (450 kg yr^–1^). A spatial domain between −50° to 50° longitude and 20° to 80° latitude was defined, with the same four vertical layers as the local simulation, using a spatial resolution of 0.1°. The same Gaussian turbulence model, stack height and log-normal modes were used for this simulation. Detailed information about the atmospheric dispersion modeling can be found in Supporting Information-2 (SI-2).

### 2.8 QA/QC

Instrumental limits of detection (ILDs) and quantification (ILQs) were determined using the standard error of residuals for the lowest three points on the calibration curve, divided by the slope of the calibration curve and multiplied by 3.3 and 10 for ILD and ILQ, respectively. Due to considerable variability in matrix effects among samples, sample-specific method detection and quantification limits (MDLs and MQLs, respectively) were calculated by dividing the areas corresponding to the response of the ILD and ILQ by the area of the internal standard in the sample. These values were then multiplied by the amount of internal standard added to each sample and then converted to concentrations (in units of pg m^–3^) using the equation for the calibration curve and the amount of air sampled (Tables S5 and S6). Additionally, general (nonsample specific) MDLs and MQLs were calculated by taking the mean IS response of all samples and using the calculation above with the mean amount of air sampled (Table S4). For figures, concentrations between MDL and MQL were used as-is, while those below MDL were substituted with zero. When an IS was missing, the concentrations were also substituted with zero. For calculations, the concentrations below sample MDL were substituted with the general, nonsample specific MDL divided by the square root of two (MDL/√2).

To assess accuracy and precision of our entire sampling and sample handling procedure, triplicate baked filters were spiked with a 3000 pg mixture of native PFAS and subjected to the same transport and storage conditions as the samples. Immediately prior to extraction, the QC samples were fortified with ISs and then extracted in the same manner as real samples. Recoveries therefore reflect losses encountered during transport and storage. Contamination introduced during sampling and sample handling was monitored using triplicate field blanks, collected at different time points during the campaign by inserting filters in the sampler and directly removing and packaging them according to the same method as the samples. Finally, contamination introduced in the laboratory was monitored by extracting triplicate unused, baked QFFs together with real samples.

Analysis of fortified QFFs revealed that recoveries of target PFAS, with accuracies (±standard deviation) for 14 of 16 substances ranged from 77 ± 4.7% (PFTriDA) to 116 ± 2.4% (PFOS; Table S3). These data indicate minimal losses during QFF transport and storage, and acceptable performance of surrogate (i.e., nonexactly matched) ISs. The only exception was PFTeDA, which displayed lower recoveries but good repeatability (17 ± 7.9%). PFTeDA might be less well extracted compared to its shorter-chained surrogate IS (^13^C_2_–PFDoDA). Overall, reported levels for PFTeDA might be significantly lower than the actual air concentrations because the IS was added to the QFFs prior to extraction in the laboratory.

Despite good IS-corrected recoveries, matrix-induced ionization suppression was significant across all samples, as shown by low and often variable IS responses (Table S9). This was particularly problematic for HFPO-DA and the long-chain PFCAs (PFUnDA, PFDoDA, PFTriDA, and PFTeDA), and tended to be more severe in weekly-, compared to daily samples (i.e., when more air was sampled). Nevertheless, significant associations between PFAS concentrations and IS recoveries were not observed, indicating that PFAS concentrations were unaffected by variable IS response, and suitable for use. To account for impacts of variable IS recoveries on detection limits, we calculated both sample- and target specific MDLs. Further, when an IS was not observable, the target was reported as “No IS” (Tables S4–S6). MDLs and MQLs were generally higher for HFPO-DA than other targets, which was partly attributable to matrix effects, but also the tendency of this substance to undergo decarboxylation, which can occur thermally, in combination with certain polar aprotic solvents and readily in the ESI source of mass spectrometers (Figure S1).^56−58^ We partly mitigated this effect by summing the area responses of both the molecular ion ([M-H]^−^, *m*/*z* = 328.9670), major in-source fragment ([M-CO_2_H]^−^, *m*/*z* = 284.9780), HCO_3_-adduct ([M + HCO_3_]^−^; *m*/*z* = 390.9676) and dimer-adduct ([2M-H]^−^, *m*/*z* = 658.9422) of HFPO-DA in both the calibration standards and the samples. For the IS, a similar summing strategy was carried out with the isotopically labeled fragments. Nevertheless, additional work is still needed to improve the detection limits of this substance.

## 3 Results and Discussion

### 3.1 HFPO-DA

#### 3.1.1 Measured Ambient Concentrations

HFPO-DA was detected in 18 out of 43 samples (detection frequency of 41%) at concentrations ranging from 0.31 to 98.66 pg m^–3^. At its peak, HFPO-DA concentrations were approximately 25-fold higher than the PFAS with the second-highest measured concentration (i.e., PFOA; 4.02 pg m^–3^), highlighting the importance of including this target as part of routine monitoring. Analysis of wind direction during the campaigns showed that HFPO-DA was commonly present when the wind was blowing from a southwesterly direction, consistent with the location of the plant (Figure 3).

Following multiple spikes in HFPO-DA in June, concentrations dropped and remained at close to- or below MDLs from July-September (<MQL-2.17 pg m^–3^), followed by a spike at the end of October (maximum of 12.21 pg m^–3^; Figure 1). The consistently lower levels from July-September may be attributed to installation of the abatement systems on the stack of the Chemours plant around this time, but the exact date at which the abatement system became fully operational is not known. Nevertheless, considering that the emissions of HFPO-DA after implementation of the abatement process were reduced by about 99% from 450 to 3.2 kg yr^–1^, some breakthrough can be expected, especially since Chemours was still fine-tuning the system during this period (Personal communication with Marc Reijmers, a Chemours employee at the time of communication).^46,59^

Similar to the mixed scenario simulation, the samples were divided into two periods assuming the emission abatement started during the first weekly sample (WS1). Median concentrations of HFPO-DA were 0.44 pg m^–3^ and <LOD for the first 16 samples (NL1–NL16) and the last 27 samples (WS1–DS13) respectively, while mean concentrations for these two periods were 11.51 and 1.31 pg m^–3^ respectively. A Mann–Whitney *U* test showed that the median concentration during the first period was not significantly greater than the second period (*U* = 139, *p* = 0.051). However, this result is borderline at the 5% significance level and possibly influenced by the concentrations of HFPO-DA below MDLs for many of the measurements (25 out of 43 samples).

Other factors could also have contributed to the lower measured concentrations of HFPO-DA after the first period. For instance, saturation of the QFFs with particulates may have occurred due to the high sampling volumes of the weekly samples (i.e., from July–October), causing lower sampling flow rates and thus less captured PFAS.

#### 3.1.2 Atmospheric Dispersion Modeling

The FLEXPART model demonstrated strong predictive accuracy for ambient HFPO-DA concentrations, with statistical analyses showing good agreement (Pearson’s *r* = 0.83, *p* ≤ 0.05, Wilmott’s *d* = 0.71, Mean Absolute Error (MAE) = 3.66 pg m^–3^) between measured and modeled values (Tables S14, S15 and Figure S7). These results confirmed our hypothesis that Chemours serves as the local point source for airborne particle-bound HFPO-DA. While the model slightly underestimated peak concentrations at Cabauw (Mean Bias Error (MBE) = −3.06 pg m^–3^), these discrepancies likely resulted from using simplified emission estimates and assuming constant emission rates throughout the sampling period. According to the model results based on the permitted emission rates before and after abatement (95 kg yr^–1^ and 3.2 kg kg yr^–1^ respectively), mean concentrations of HFPO-DA at the sampling site decreased by 90.2% from 4.6 to 0.45 pg m^–3^, while measured mean concentrations decreased by 88.6% from 11.51 to 1.31 pg m^–3^.

To assess contamination patterns around the Chemours FPP, we analyzed daily atmospheric concentrations and both wet and dry deposition during June 2021 using a 95 kg yr^–1^ emission scenario (Figure S8). Similar to findings reported by D’Ambro et al.^60^ for a North Carolina Chemours facility, the highest deposition rates occurred closest to the source, reaching approximately 1 μg m^–2^ within 556 m of the facility. Deposition decreased with distance, dropping to around 0.1 μg m^–2^ day^–1^ between 556 and 1.7 km, and further declining to 0.03 μg m^–2^ day^–1^ between 1.7 and 2.8 km. Atmospheric concentrations followed a similar pattern, peaking at roughly 10 ng m^–2^ near the source and decreasing to 1.8 and 0.5 ng m^–2^ day^–1^ at the respective distance ranges.

The modeled results above align with recommendations made in 2021 by the Dutch National Institute of Public Health and Environment (RIVM) against consuming vegetables grown within 1 km of the Dordrecht facility due to HFPO-DA and PFOA contamination.^61^ However, both our results and a study at a Chemours site in West Virginia suggest that significant daily deposition could continue beyond this radius (Figure S8).^36^ Given HFPO-DA’s high persistence and historical emissions, areas outside the current radius may also face substantial contamination, potentially warranting a reassessment of existing guidelines.

To understand broader regional impacts, we modeled HFPO-DA dispersion across Europe under unabated emissions (450 kg yr^–1^). The model estimated annual total (wet and dry) deposition fluxes of 30–142 μg m^–2^ yr^–1^ near the source in Dordrecht, with significant deposition extending predominantly to the northeast (Figure S9 and Table S16). Distant cities, such as London and Hamburg, located 298 km west and 481 km northeast respectively, showed similar deposition ranges of 36–172 and 59–279 ng m^–2^ yr^–1^. Even Reykjavik, over 3000 km from the source, received measurable deposition of 0.5–2.4 ng m^–2^ yr^–1^. These results demonstrate HFPO-DA’s capacity for long-range atmospheric transport and establish a clear link between the Dordrecht FPP and widespread European contamination. More details on the results of the large-scale FLEXPART simulations are provided in SI-2.

### 3.2 Capstone FS10 (6:2 FTSA)

**Figure 2:**
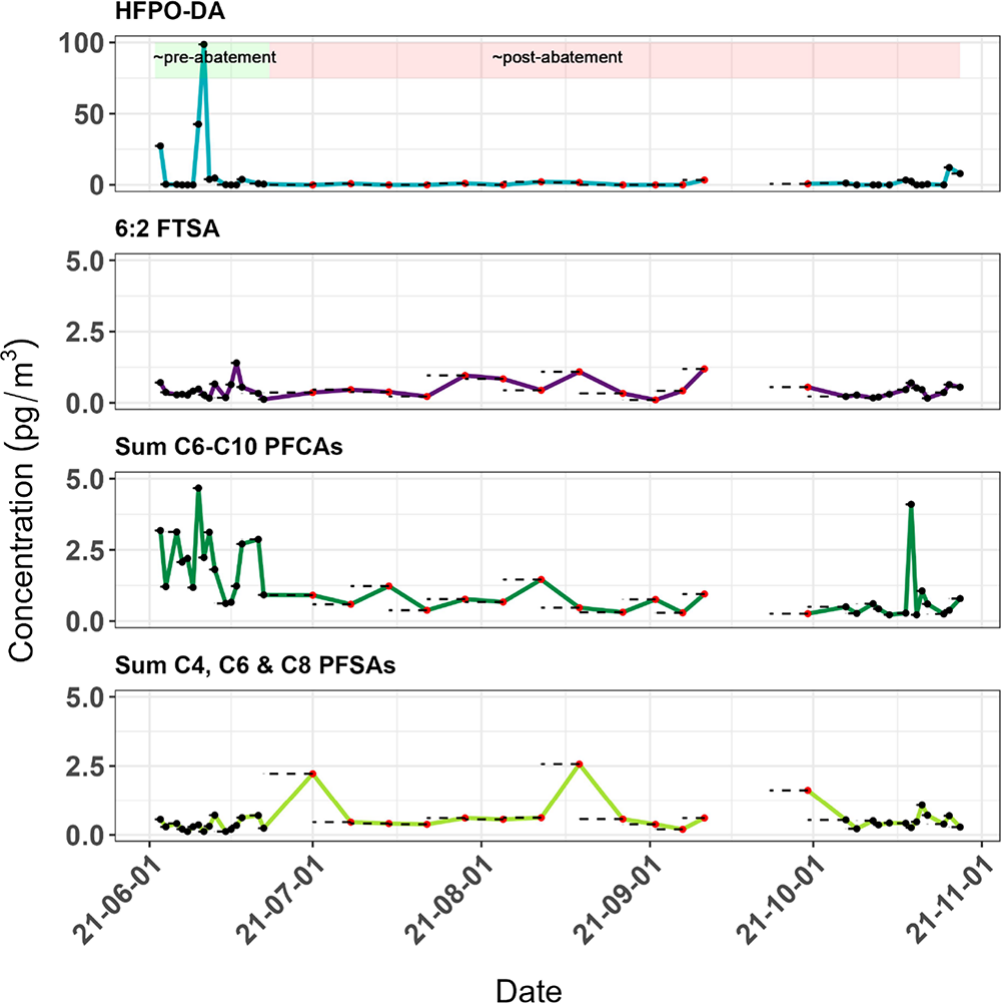
Measurements of PFAS concentrations in air (pg m^–3^) sampled from the Cabauw meteorological observatory over 5 months. The black points indicate samples taken during the daily sampling campaigns, while the red points represent the samples taken during the weekly sampling campaign. The interruption in the sampling was due to a power failure. Note the different scale for HFPO-DA.

The other processing aid used at Chemours Dordrecht, Capstone FS10 or 6:2 FTSA (used for producing FKM), was measured in all samples (0.10–1.40 pg m^–3^) but at much lower concentrations than the maximum observed for HFPO-DA (Figure 2). Less FKM is produced at Chemours compared to PTFE and FEP, which in turn leads to less use and subsequent emissions of 6:2 FTSA compared to HFPO-DA in the unabated scenario.^46^ Additionally, 6:2 FTSA is longer-chained and more hydrophobic than HFPO-DA and is therefore potentially more readily removed from the FKM dispersion after polymerization (personal communication with Frenk Hulsebosch from Chemours). Lastly, unlike part of the PTFE and FEP produced in Dordrecht, FKMs are not dried to a fine powder, also limiting potential emissions of fluorinated processing aid to the atmosphere.^47^ The stacks of the FKM production process are not connected to the Sequoia abatement system and air emissions of 6:2 FTSA (3 kg yr^–1^) were already within regulatory limits. As such, this might explain why, as opposed to HFPO–DA, a noticeable decrease in peak air concentrations of 6:2 FTSA was not observed during our sampling campaigns.

**Figure 3:**
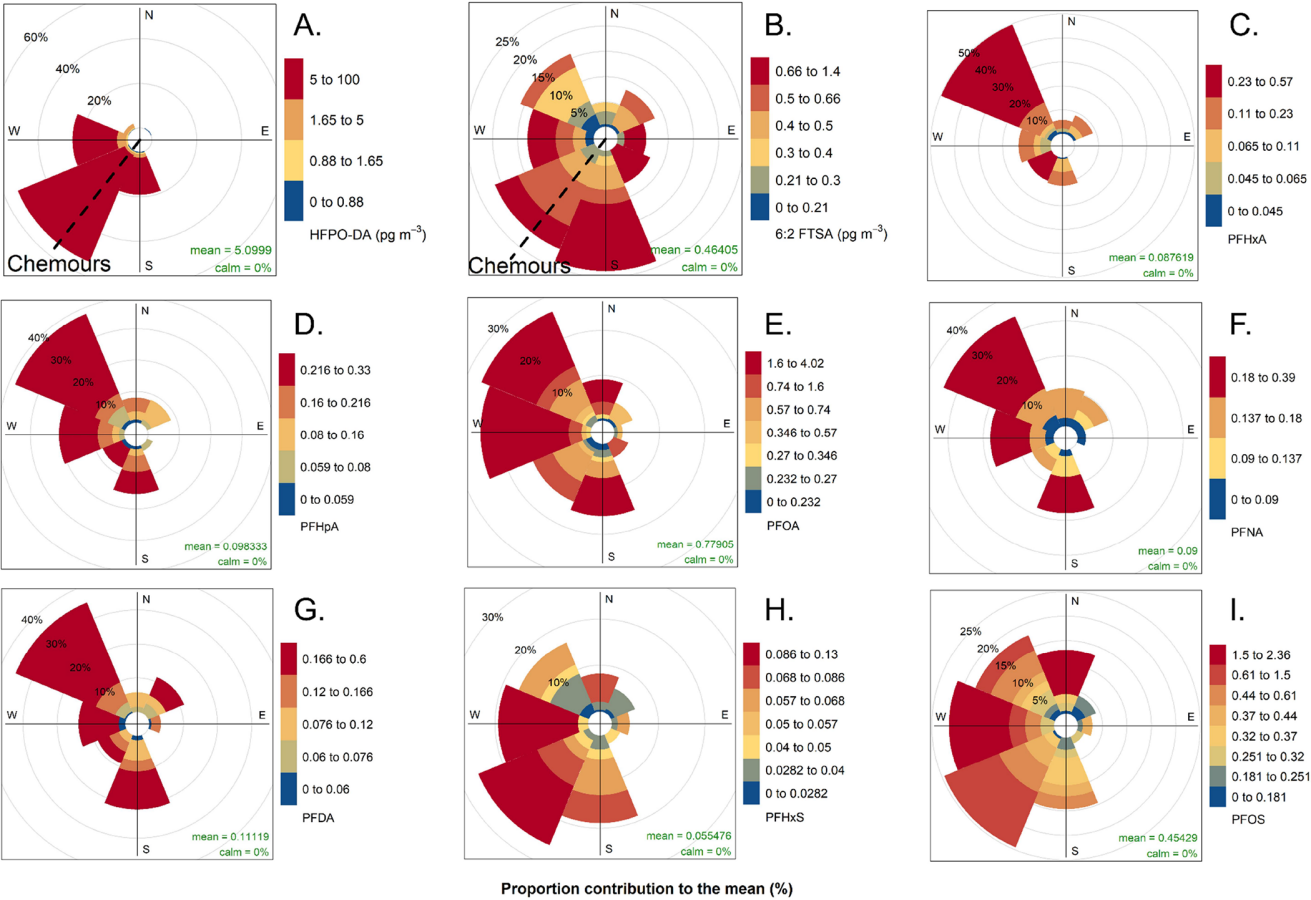
Pollution roses for the HFPO-DA (A) and 6:2 FTSA (B), C6–C10 PFCA (C–G), PFHxS (H), and PFOS (I) air concentrations at the Cabauw observatory during the sampling campaigns, showing which wind directions contributed most to the overall mean air concentrations of these PFAS. The direction of the Chemours FPP is indicated by the dashed line on the wind roses for HFPO-DA and 6:2 FTSA. More information on the derivation of these figures can be found in Section S1.1 of the SI.

Consistent with observations of HFPO-DA, higher levels of 6:2 FTSA were associated with wind coming from the direction of the plant (Figure 3). However, these peaks were not as pronounced as the peak observed for HFPO-DA. Air concentrations of HFPO-DA and 6:2 FTSA had a nonsignificant positive association (Spearman’s *r* 0.21: *p* = 0.19; Table S12). Therefore, as 6:2 FTSA is also a component of aqueous film forming foams (AFFF) it could have other sources besides the Chemours plant.^62^ Additionally, this substance has been used and emitted at the FPP and other sites for a longer period of time compared to HFPO-DA, likely leading to a difference between the environmental background of this substance and HFPO-DA in The Netherlands. This may explain why 6:2 FTSA was also associated with other wind directions besides those coming from the Chemours plant (Figure 3). Lastly, MDLs for 6:2 FTSA were considerably lower than those of HFPO-DA, probably leading to the quantification of this substance in more samples due to the higher sensitivity of the method (Tables S5–S7).

### 3.3 Other Target PFAS

Other target PFAS detected during the Dutch campaign included PFBA, PFPeA, PFHxA, PFHpA, PFOA, PFNA, PFDA, PFUnDA, PFDoDA, PFTriDA, PFTeDA, PFBS, PFHxS, and PFOS (Table S7). Partly due to poor recoveries of the internal standards and low sensitivity for these substances in the matrix, PFBA, PFTeDA, PFPeA, and PFTriDA were only detected in one or two samples. The C_6_–C_12_ PFCAs displayed higher detection frequencies: PFHxA (58%), PFHpA (63%), PFOA (100%), PFNA (56%), PFDA (72%), PFUnDA (28%), PFDoDA (21%). The perfluoroalkanesulfonic acids (PFSAs) PFBS (30%), PFHxS (100%), PFOS (100%) were also detected.

Peak levels of PFAAs were lower than those of HFPO-DA (98.66 pg m^–3^), with PFOA having the second highest peak level (4.02 pg m^–3^), followed by PFOS (2.33 pg m^–3^). Generally, levels of other measured PFAS were less variable than those of HFPO-DA and did not show a similarly high peak as observed for HFPO-DA. Additionally, average HFPO-DA concentrations during the entire sampling campaign (5.10 pg m^–3^) exceeded the average of the sum of all other detected PFAS in the air samples (2.45 pg m^–3^). The average detected ΣPFAA air levels were around five times higher than background levels measured in Central Europe^63^ and were 1.25 pg m^–3^ for ΣC_6_–C_10_ PFCAs, 0.55 pg m^–3^ for ΣC_4_, C_6_, and C_8_ PFSAs and 0.46 pg m^–3^ for 6:2 FTSA respectively.

Dominant wind directions for air concentrations of PFCAs were as follows (Figure 3): PFHxA (NW), PFHpA (NW), PFOA (NW, W, S), PFNA (NW, W, S), PFDA (NW, S), PFUnDA (SW), PFDoDA (S) and for PFSAs: PFBS (N, NW, S, SW), PFHxS (SW, W, NW), PFOS (NW, SW). Notably, high air PFAS levels were often not associated with easterly winds. However, easterly winds were relatively uncommon during the sampling period (Figure S2 and Table S11 show the wind rose and wind direction distribution during sampling).

Some PFAS levels were correlated (Spearman’s *r*: *p* ≤ 0.05, Table S12). Most notably, HFPO-DA concentrations were moderately positively associated with levels of PFOA (*r* = 0.39 and *p* ≤ 0.01) and PFHpA (*r* = 0.31 and *p* ≤ 0.05). The Dordrecht plant used APFO as a processing aid until 2012 and has emitted considerable amounts of PFOA to the local environment,^64,65^ possibly explaining the positive associations between concentrations of HFPO-DA and PFOA and PFHpA. Chemours’ emissions of PFOA to air between 1998 and 2012 amounted to about 13 t in total.^66^ Correlations between C_4_–C_6_, C_9_–C_14_ PFCAs and HFPO-DA were not statistically significant (*p* > 0.05, Table S12). Although it is possible that the wind sector of the Chemours site is a source area of residual PFOA and other associated PFCAs, our data indicates that these substances could have multiple additional source areas, including a possible source northwest of the sampling site (Figure 3). Similar to HFPO-DA, the peak concentrations of PFHxA, PFHpA, PFOA, and PFDA all decreased during sampling (see Table S8), possibly showing the effect of the abatement system. However, a seasonal or sampling effect cannot be completely ruled out, as sampling started in the summer and ended in the autumn. These effects could include meteorological conditions; total radiation and temperature were higher during the first period (Figure S10), while the second period saw lower temperatures and more precipitation. Sampling effects could be the caused by the higher sampling volumes during weekly sampling or the increased matrix effects from these samples leading to higher MDLs. These could confound the lower observed levels of PFCAs during this second period. Additionally, the PFSAs (PFBS, PFHxS, and PFOS) were moderately positively associated with each other, as were some short-chain (C_4_–C_7_) and long-chain (C_8_–C_14_) PFCAs (*r* > 0.30 and *p* ≤ 0.05; Table S12).

The influence of sea spray aerosol (SSA) on levels of PFAS was found to be weak. No statistically significant correlation between the sodium (Na^+^) tracer ion and any of the measured PFAS was found (Table S12). Both PFHxS and PFOS had a moderately positive nonsignificant Spearman’s *r* with air concentrations of Na^+^ (0.26 and 0.20, with *p* = 0.1 and 0.2 respectively; Table S12). The dominant wind directions associated with higher levels of the Na^+^ tracer ion corresponded closely with the location of the North Sea. This suggests a possible potential for the ocean to act as one of the sources of these long-chain PFSAs to the ambient air at the sampling site (Figures 1, 3H–I and S3), although additional sampling at the site and at sites closer to the shore is needed to investigate the importance of this source in relation to other sources and to increase the statistical power of this association.^51^

Other potential regional atmospheric PFAS sources include a 3M site in the Port of Antwerp (80 km SW) that is a historical source of PFSAs and their precursors to the environment through the production of Scotchgard stain repellents.^67^ A waste incineration plant operated by Indaver NV with recorded atmospheric emissions of HFPO-DA and other PFAS is also located close to this site.^68^ Activated carbon used in the emission abatement of Chemours is incinerated at this plant. Additionally, large industrial areas with (petro)chemical industries, such as the Port of Rotterdam (40 km W), the Moerdijk (40 km SW), Sloegebied (100 km SW) and Dow Chemical (100 km SW) are located relatively close by. The sampling site is also in close proximity to both Schiphol (40 km N) and Rotterdam (30 km W) airports, which could be potential sources of AFFF-related PFAS. Lastly, due to the high population density of The Netherlands, many highly populated urban centers can be found in all directions within 30 km of the sampling site. Overall, we cannot rule out that some of the measured PFAA air concentrations reflect local background levels, considering historical emissions in the region, the low variability of measured air concentrations and the high population density of The Netherlands.^69^

### 3.4 Suspect Screening

Suspects identified in an earlier study in surface water PFAS close to Chemours Dordrecht by Gebbink et al.^33^ could not be identified in this study. However, a number of other suspects were identified in the air samples which may be associated with the FPP (Table S14).

#### 3.4.1 Hydrogen-Substituted Perfluoroalkyl Carboxylic Acids

A homologous series of suspects was identified as hydrogen-substituted perfluoroalkyl carboxylic acids (H-PFCAs) and included at least 7 homologues (H-PFHxA, H-PFHpA, H-PFOA, H-PFNA, H-PFDA, H-PFUnDA, and H-PFDoDA), putatively identified by additional fragmentation (MS^2^ spectra), exact masses, retention times and fragmentation patterns (with [M-CHO_2_F]^−^ being a common in-source loss; Figure S6).^70^ Acquisition of authentic standards of H-PFOA, H-PFNA, and H-PFUnDA confirmed the identities of these homologues. However, our attempts to reanalyze sample extracts together with H-PFCA calibration curves revealed higher baseline noise and weaker responses than the original chromatograms, hindering quantification. As a result, H-PFCA concentrations were semiquantified using calibration curves of their fully fluorinated analogs.

Estimated ∑H-PFCA concentrations ranged from 0.01 to 2.71 pg m^–3^. The air concentrations of H-PFCAs peaked in the same sample as the air concentrations of HFPO-DA (Figure S2), suggesting that H-PFCAs were released from the same source as HFPO-DA. H-PFCAs are commonly encountered near fluoropolymer production plants that produce PTFE.^34,71^ On C18 stationary phases, H-PFCAs nearly coelute with PFCAs with one less perfluorinated carbon (e.g., H-PFOA almost coelutes with PFHpA), indicating that the H atom on the terminal carbon significantly reduces the hydrophobicity of the perfluoroalkyl chain. In-source formation of H-PFCAs from PFCAs (suggested previously by Barrett et al.) was ruled out based on chromatographic separation (Figure S5).^72^ Further, H-PFCAs were absent in PFCA standards, spike/recovery tests, lab blanks or field blanks in the present work. Overall, detection of H-PFCAs here corroborate prior findings by Chemours,^73^ which reported formation of H-capped PTFE chains with carboxylic groups after TFE polymerization, and Strynar et al., who reported H-capped polyvinylidene fluoride (PVDF) chains with carboxylic groups around a FPP that produces PVDF.^74^ Additionally, Sworen et al. further elaborated potential formation pathways of H-PFCAs, as well as some of the suspects reported by Gebbink et al. as byproducts formed during the polymerization of PTFE.^75,33^

#### 3.4.2 Bisphenol AF

Minor traces of the fluoroelastomer curing agent bisphenol AF (BPAF; *m*/*z* = 335.0512) were identified in 1 sample collected from Cabauw. Reanalysis of the samples together with a calibration curve prepared with a standard of BPAF confirmed the identification and revealed a concentration of 0.11 pg m^–3^, which coincided with the wind direction from the plant. However, because this level was also close to the MDL and the substance was only detected in one sample, additional work is needed to determine whether the Chemours FPP is a point source of BPAF to the air.

### 3.5 Implications

Overall, this study provides the first evidence that regulatory action and implementation of abatement systems by the fluoropolymer production industry have been effective in reducing direct emissions and resulting environmental levels of the processing aid HFPO-DA at the Chemours Dordrecht plant. Furthermore, it was shown that part of the previous unabated emissions could have undergone long-range atmospheric transport to locations hundreds to thousands of kilometers away from the point source. While emissions from the plant were reduced by the emission abatement system, HFPO-DA in spent activated carbon could cause emissions and pose waste management constraints at the off-site incineration plant. Furthermore, the results of this study, as well as the findings from a previous review of FPP emissions,^9^ demonstrate the importance of including more PFAS than only fluorinated processing aids in future monitoring of air surrounding FPPs. Examples include volatile PFAS, such as E1, Ether A, Ether B and various HFCs, which could unfortunately not be measured using the methods of this study. From data in the environmental permit it was apparent that emissions of PFAS to air from the plant occurred already prior to the polymerization process; mainly through synthesis and oligomerization of monomers. Polymerization and further processing, such as fluoroelastomer curing, could contribute to additional emissions of processing aids and other PFAS; as indicated by the detection of H-PFCAs and bisphenol-AF in air samples in the present work. Nevertheless, many uncertainties regarding emissions from the Chemours FPP still remain, including the environmental fate of many of the PFAS identified here, and the risk they pose to the surrounding population. Additional investigations into these substances, stack or fence-line sampling along with further long-term air monitoring is therefore clearly needed, and should be carried out by the responsible authorities.

## Supporting Information Available

Target analyte list, instrumental settings used, calculations, meteorological observations, sample MDLs, sample MQLs, IS recoveries, sample concentrations, and additional study information and statistics (PDF)
Additional information on the FLEXPART atmospheric dispersion modeling (PDF)
