# Supplementary material for: HFPO-DA and Other PFAS in Air Downwind of a Fluoropolymer Production Plant in the Netherlands: Measurements and Modeling
Source: Environ Sci Technol. 2025 Apr 21;59(17):8662–72. doi: 10.1021/acs.est.4c13943 (PMC12060278; doi:10.1021/acs.est.4c13943)
Supplement: Supplementary file 1 — es4c13943_si_001.pdf [file es4c13943_si_001.pdf]

Supporting information 1 for 'HFPO-DA and other PFAS in air downwind of a fluoropolymer production plant in the Netherlands: measurements and modeling'

Joost Dalmijn[a]* Julia J. Shafer[a], Jonathan P. Benskin[a], Matthew E. Salter[a], Jana H. Johansson[b] and Ian T. Cousins[a]*

[a] Department of Environmental Science, Stockholm University, SE-10691 Stockholm, Sweden
[b] Department of Thematic Studies—Environmental Change, 581 83 Linköping University, Linköping, Sweden
*E-mail: joost.dalmijn@aces.su.se
*E-mail: ian.cousins@aces.su.se

Summary: 35 pages, 17 tables, 10 figures.

S1.1 Wind direction analysis

Average wind speed and direction over the sampling periods were calculated from the hourly data using the following equations:

1. $V_{East(i)} = mean(WS_{(i)} * sin(WD_{(i)} * \frac{\pi}{180}))$

2. $V_{North(i)} = mean(WS_{(i)} * cos(WD_{(i)} * \frac{\pi}{180}))$

3. $WD_{avg} = 360 + atan2(V_{East}, V_{North}) * \frac{180}{\pi}$ (°)
4. $WS_{avg} = \sqrt{(V_{East}{}^2 + V_{North}{}^2)}$ (ms^{-1})

In which V is the vector of wind speed (WS, ms^{-1}), wind direction (WD, °) and atan2 is the 2-argument arctangent. Subsequently, the averaged wind speed and wind direction data, along with the PFAS air concentration data were used as input for the pollutionRose function of the openair R package 1 for plotting the pollution roses of the data. These plots take the mean air concentrations of an analyte from all the samples (5.) and plot the sums of the proportions (%, 6.) individual concentrations contribute to this mean concentration at different wind directions.

5. $\frac{\sum air\ concentrations}{n}$

6. $\frac{\sum air\ conentrations}{single\ concentration} \times 100$

Bin widths for the wind roses were determined by taking the square root of the number of samples > MDL and rounding up.

Table S1. Information (name, acronym, formula, CAS number, and exact mass) for PFAS targets included in the present work, along with their internal standards (ISs). Recovery standards (RSs; ***bold and italics***) are also shown for PFOS and PFOA.

**Name**	**Formula**	**Internal standard**	**CAS**	**Exact mass**
**Perfluoroalkyl carboxylic acids (PFCAs)**				
Perfluorobutanoic acid (PFBA)	$C_4HF_7O_2$	$^{13}C_4$ PFBA	375-22-4	212.9792
Perfluoropentanoic acid (PFPeA)	$C_4HF_9O_2$	$^{13}C_5$ PFPeA	2706-90-3	262.9760
Perfluorohexanoic acid (PFHxA)	$C_6HF_{11}O_2$	$^{13}C_2$ PFHxA	307-24-4	312.9728
Perfluorocyclohexane carboxylic acid (PFCHCA)	$C_7HF_{11}O_2$	$^{13}C_2$ PFHxA	374-88-9	324.9729
Perfluoroheptanoic acid (PFHpA)	$C_7HF_{13}O_2$	$^{13}C_4$ PFHpA	375-85-9	362.9696
Perfluorooctanoic acid (PFOA)	$C_8HF_{15}O_2$	$^{13}C_4$ PFOA<br>***$^{13}C_8$ PFOA***	335-67-1	412.9666
Perfluorononanoic acid (PFNA)	$C_9HF_{17}O_2$	$^{13}C_5$ PFNA	375-95-1	462.9632
Perfluorodecanoic acid (PFDA)	$C_{10}HF_{19}O_2$	$^{13}C_5$ PFDA	335-76-2	512.9600
Perfluoroundecanoic acid (PFUnDA)	$C_{11}HF_{21}O_2$	$^{13}C_2$ PFUnDA	2058-94-8	562.9568
Perfluorododecanoic acid (PFDoDA)	$C_{12}HF_{23}O_2$	$^{13}C_2$ PFDoDA	307-55-1	612.9536
Perfluorotridecanoic acid (PFTriDA)	$C_{13}HF_{25}O_2$	$^{13}C_2$ PFDoDA	72629-94-8	662.9504
Perfluorotetradecanoic acid (PFTeDA)	$C_{14}HF_{27}O_2$	$^{13}C_2$ PFDoDA	376-06-7	712.9472
Perfluorohexadecanoic acid (PFHxDA)	$C_{16}HF_{31}O_2$	$^{13}C_2$ PFDoDA	67905-19-5	812.9408
Perfluorooctadecanoic acid (PFOcDA)	$C_{18}HF_{35}O_2$	$^{13}C_2$ PFDoDA	16517-11-6	912.9345
**Perfluoroalkane sulfonic acids (PFSAs)**				
Perfluorobutane sulfonic acid (PFBS)	$C_4HF_9O_3S$	$^{18}O_2$ PFHxS	375-73-5	298.9429
Perfluorohexane sulfonic acid (PFHxS)	$C_6HF_{13}O_3S$	$^{18}O_2$ PFHxS	355-46-4	398.9366
Perfluorooctane sulfonic acid (PFOS)	$C_8HF_{17}O_3S$	$^{13}C_4$ PFOS<br>***$^{13}C_8$ PFOS***	1763-23-1	498.9302
Perfluorodecane sulfonic acid (PFDS)	$C_{10}HF_{21}O_3S$	$^{13}C_4$ PFOS	335-77-3	598.9238
**Perfluoroalkylether carboxylic acids (PFECAs)**				
Perfluoro-3-methoxymethanoic acid (PMPA)	$C_4HF_7O_3$	$^{13}C_4$ PFBA	377-73-1	228.9738
Perfluoro-4-methoxybutanoic acid (PMBA)	$C_5HF_9O_3$	$^{13}C_5$ PFPeA	863090-89-5	278.9709
Perfluoro-3-tetrahydro-2-furancarboxylic acid (Furan-PFECA)	$C_5HF_7O_3$	$^{13}C_5$ PFPeA	65578-62-3	196.9836
Perfluoro-3,6-dioxaheptanoic acid (PFO2HpA)	$C_5HF_9O_5$	$^{13}C_2$ PFHxA	151772-58-6	200.9787

Hexfluoropropylene oxide dimer acid (HFPO-DA)	$C_6HF_{11}O_3$	$^{13}C_3$ HFPO-DA	13252-13-6	284.9780
Perfluoro-4-isopropoxybutanoic acid (PFIPBA)	$C_7HF_{13}O_3$	$^{13}C_4$ PFOA	801212-59-9	378.9642
Perfluoro-3,6-dioxaoctanoic acid (PFO2OA, EEA)	$C_6HF_{11}O_4$	$^{13}C_4$ PFHpA	80153-82-8	250.9760
3H-Perfluoro-3-[(3-methoxy-propoxy)propanoic acid (DONA)	$C_7H_2F_{12}O_3$	$^{13}C_4$ PFHpA	919005-14-4	376.9689
8-Iodoperfluoro(6-oxaoctanoic) acid (IPFOOA)	$C_7HF_{12}IO_3$	$^{13}C_4$ PFOA	948014-44-6	486.8701
Perfluoro-3,6-dioxadecanoic acid (PFO2DA)	$C_8HF_{15}O_4$	$^{13}C_4$ PFOA	137780-69-9	350.9691
2-Perfluoropentoxy-2,3,3,3-tetrafluoropropanoic acid (PFPeOPA)	$C_8HF_{15}O_3$	$^{13}C_4$ PFOA	504435-11-4	428.9608
Perfluoro-3,6,9-trioxadecanoic acid (PFO3DA)	$C_7HF_{13}O_5$	$^{13}C_4$ PFOA	151772-59-7	316.9674
Hexafluoropropylene oxide trimer acid (HFPO-TA)	$C_9HF_{17}O_4$	$^{13}C_5$ PFNA	13252-14-7	184.9834
Perfluoro-3,6,9-trioxatridecanoic acid (PFO3TriDA)	$C_{10}HF_{19}O_5$	$^{13}C_5$ PFDA	330562-41-9	466.9575
Hexafluoropropylene oxide tetramer acid (HFPO-TeA)	$C_{12}HF_{23}O_5$	$^{13}C_2$ PFDoDA	65294-16-8	350.9691
Hexafluoropropylene oxide pentamer acid (HFPO-PeA)	$C_{15}HF_{29}O_6$	$^{13}C_2$ PFDoDA	65150-95-0	516.9550
Hexafluoropropylene oxide hexamer acid (HFPO-HxA)	$C_{18}HF_{35}O_7$	$^{13}C_2$ PFDoDA	52481-85-3	682.9400
**Fluorotelomer sulfonic acids (FTSAs)**				
4:2 fluorotelomer sulfonic acid (4:2 FTSA)	$C_6H_5F_9O_3S$	$^{13}C_2$ 6:2 FTSA	75746-90-8	326.9743
6:2 fluorotelomer sulfonic acid (6:2 FTSA)	$C_8H_5F_{13}O_3S$	$^{13}C_2$ 6:2 FTSA	27619-97-2	426.9679
8:2 fluorotelomer sulfonic acid (8:2 FTSA)	$C_{10}H_5F_{17}O_3S$	$^{13}C_2$ 6:2 FTSA	251099-16-8	526.9615
**Fluoroalkanesulfonamides (FASAs)**				
Perfluorooctanesulfonamide (FOSA)	$C_8H_2F_{17}NO_2S$	$^{13}C_8$ FOSA	754-91-6	497.9462
N-ethylperfluorooctanesulfonamide (N-EtFOSA)	$C_{10}H_6F_{17}NO_2S$	d3-MeFOSAA	4151-50-2	525.9775
N-methylperfluorooctanesulfonamide (N-MeFOSA)	$C_9H_4F_{17}NO_2S$	d3-MeFOSAA	865-71-4	511.9618
Perfluorooctanesulfonamido acetic acid (FOSAA)	$C_{10}H_4F_{17}NO_4S$	d3-MeFOSAA		555.9517
N-ethylperfluorooctanesulfonamido acetic acid (N-EtFOSAA	$C_{12}H_8F_{17}NO_4S$	d3-MeFOSAA	1336-61-4	583.9830
N-methylperfluorooctanesulfonamido acetic acid (N-MeFOSAA)	$C_{11}H_6F_{17}NO_4S$	d3-MeFOSAA	2355-31-9	569.9673
N-ethylperfluorooctanesulfonamidoethanol (N-EtFOSE)	$C_{12}H_{10}F_{17}NO_3S$	d3-MeFOSAA	1691-99-2	630.0248
N-methylperfluorooctanesulfonamidoethanol (N-MeFOSE)	$C_{11}H_8F_{17}NO_3S$	d3-MeFOSAA	24448-09-7	616.0092
**Chlorinated perfluoroalkylether sulfonic acids (Cl-PFESAs)**				

2-(6-chloro-dodecafluorohexyloxy)-tetrafluoroethane sulfonic acid (F53-B Major)	$C_8HClF_{16}O_4S$	$^{13}C_5$ PFDA	756426-58-1	530.8956
11-Chloroeicosafluoro-3-oxaundecane-1-sulfonic acid (F53-B Minor)	$C_{10}HClF_{20}O_4S$	$^{13}C_5$ PFDA	83329-89-9	630.8892
**Hydrogen-substituted perfluoroalkyl carboxylic acids (H-PFCAs)***				
H-perfluorooctanoic acid (H-PFOA)	$C_8H_2F_{14}O_2$	$^{13}C_4$ PFOA	13973-14-3	394.9758
H-perfluorononanoic acid (H-PFNA)	$C_9H_2F_{16}O_2$	$^{13}C_5$ PFNA	76-21-1	444.9726
H-Perfluoroundecanoic acid (H-PFUnDA)	$C_{11}H_2F_{20}O_2$	$^{13}C_2$ PFUnDA	1765-48-6	544.9663
**Fluorinated bisphenol***				
Bisphenol AF	$C_{15}H_{10}F_6O_2$	$^{13}C_4$ PFOA	1478-61-1	335.0512

*Analyzed at a later stage using a separate calibration curve

Table S2. Settings of UHPLC-Orbitrap analysis

**HESI settings**			
Spray voltage	3.7 kV		
Capillary temperature	320 °C		
Sheath gas	30 arbitrary units (au)		
Aux gas	10 au		
Aux gas heater temperature	320 °C		
**HRMS settings**			
AGC target	Full scan: 3 000 000, ddMS2: 500 000		
Resolution	Full scan: 120 000, ddMS2: 15 000 FWHM		
HCD	35		
**Mobile phase gradient**			
Time (min)	Mobile phase A (%)	Mobile phase B (%)	Flow (mL/min)
0.0-0.5	90	10	0.4
0.5-8.0	90>1	10>99	0.4
8.0-11.0	1	99	0.4
11.0-11.1	1>90	99>10	0.4
11.1-13	90	10	0.4

Table S3. Spike/recovery test results (ng). 3 ng spiked per substance.

Sample	PFBA	PFPeA	PFHxA	PFHpA	PFOA	PFNA	PFDA	PFUnDA	PFDoDA	PFTriDA	PFTeDA	PFBS	PFHxS	PFOS	6:2 FTS	HFPO-DA
**SR1**	2.73	2.82	2.74	2.74	3.09	2.86	2.84	3.40	3.04	2.46	0.76	1.95	3.17	3.53	2.83	2.77
**SR2**	2.67	2.58	2.58	3.09	3.03	2.79	2.77	3.27	3.04	2.27	0.49	2.47	3.00	3.41	2.81	2.44
**SR3**	2.44	2.63	2.52	2.76	2.96	2.75	2.99	3.39	2.96	2.19	0.29	2.72	3.18	3.53	2.72	2.57
**SD**	0.15	0.13	0.11	0.20	0.06	0.05	0.11	0.07	0.05	0.14	0.24	0.39	0.10	0.07	0.06	0.17
**Mean**	2.62	2.68	2.62	2.86	3.03	2.80	2.87	3.36	3.01	2.31	0.51	2.38	3.11	3.49	2.79	2.59
**Recovery (%)**	87.20	89.29	87.17	95.36	100.92	93.33	95.53	111.83	100.28	76.91	17.06	79.31	103.82	116.39	92.88	86.47
**SD (%)**	5.1	4.3	3.8	6.5	2.1	1.8	3.8	2.4	1.5	4.7	7.9	13.1	3.3	2.4	2.0	5.1

Table S4. General, non-sample-specific method detection and quantification limits (MDLs and MQLs; pg m^{-3}), calculated from the mean internal standard (IS) response of all samples.

	PFBA	PFPeA	PFHxA	PFHpA	PFOA	PFNA	PFDA	PFUnDA	PFDoDA	PFTriDA	PFTeDA	PFBS	PFHxS	PFOS	6:2 FTS	HFPO-DA
**MDL**	0.625	0.004	0.002	0.011	0.001	0.017	0.008	0.002	0.006	0.013	0.009	0.052	0.002	0.003	0.013	0.240
**MQL**	1.877	0.018	0.048	0.026	0.013	0.034	0.025	0.008	0.016	0.042	0.042	0.053	0.018	0.008	0.035	0.674
**MDL/2**	0.313	0.002	0.001	0.005	0.001	0.008	0.004	0.001	0.003	0.007	0.004	0.026	0.001	0.002	0.006	0.120

Table S5. Method detection limits (MDLs; pg m^{-3}). “No IS” indicates that the MDL could not be determined because the internal standard was not observable.

Sample	PFBA	PFPeA	PFHxA	PFHpA	PFOA	PFNA	PFDA	PFUnDA	PFDoDA	PFTriDA	PFTeDA	PFBS	PFHxS	PFOS	6:2 FTS	HFPO-DA
NL1	0.38	0.10	0.04	0.04	0.05	0.16	0.13	No IS	No IS	No IS	No IS	0.16	0.022	0.02	0.06	1.45
NL2	0.27	0.07	0.02	0.02	0.04	0.19	0.13	No IS	No IS	No IS	No IS	0.07	0.022	0.02	0.03	0.47
NL3	1.15	0.06	0.01	0.02	0.04	0.23	0.41	No IS	No IS	No IS	No IS	0.06	0.011	0.02	0.03	0.30
NL4	0.10	0.06	0.02	0.01	0.02	0.03	0.01	0.01	0.08	0.24	0.34	0.08	0.022	0.00	0.03	0.37
NL5	0.45	0.08	0.03	0.03	0.03	0.08	0.04	1.03	No IS	No IS	No IS	0.09	0.022	0.01	0.03	0.48
NL6	0.45	0.13	0.04	0.05	0.05	0.10	0.03	0.10	0.49	1.48	2.11	0.10	0.022	0.00	0.05	1.38
NL7	0.44	0.12	0.03	0.04	0.04	0.07	0.01	0.04	0.10	0.31	0.45	0.09	0.022	0.00	0.04	0.86
NL8	0.45	0.13	0.03	0.06	0.09	0.52	1.00	No IS	No IS	No IS	No IS	0.17	0.022	0.09	0.06	0.83
NL9	0.45	0.09	0.02	0.03	0.03	0.08	0.04	0.53	No IS	No IS	No IS	0.08	0.022	0.01	0.03	0.46
NL10	0.45	0.18	0.05	0.05	0.05	0.18	0.07	0.34	No IS	No IS	No IS	0.12	0.022	0.01	0.05	1.68
NL11	0.30	0.04	0.01	0.01	0.02	0.08	0.13	No IS	No IS	No IS	No IS	0.05	0.011	0.02	0.02	0.21
NL12	0.39	0.07	0.03	0.02	0.03	0.09	0.05	0.48	No IS	No IS	No IS	0.06	0.022	0.01	0.03	0.43
NL13	1.88	0.15	0.04	0.06	0.08	0.27	0.36	No IS	No IS	No IS	No IS	0.19	0.022	0.03	0.08	1.18
NL14	1.06	0.11	0.03	0.04	0.04	0.09	0.03	0.13	1.17	3.52	5.04	0.08	0.022	0.00	0.04	0.70
NL15	0.46	0.06	0.01	0.02	0.02	0.06	0.02	0.12	No IS	No IS	No IS	0.05	0.007	0.00	0.02	0.44
NL16	0.21	0.05	0.02	0.02	0.01	0.02	0.01	0.03	0.20	0.60	0.86	0.03	0.022	0.00	0.01	0.20
WS1	0.46	0.05	0.03	0.05	0.12	No IS	No IS	No IS	No IS	No IS	No IS	0.14	0.002	0.07	0.05	0.53
WS2	0.46	0.05	0.03	0.03	0.03	0.08	0.02	0.04	0.22	0.66	0.95	0.03	0.003	0.00	0.03	0.77
WS3	0.35	0.05	0.01	0.03	0.03	0.05	0.01	0.01	0.01	0.04	0.06	0.03	0.003	0.00	0.03	0.65
WS4	0.14	0.04	0.01	0.02	0.02	0.07	0.01	0.05	0.10	0.29	0.41	0.02	0.003	0.00	0.02	0.46
WS5	0.41	0.04	0.02	0.04	0.04	0.10	0.02	0.04	0.27	0.81	1.16	0.04	0.003	0.00	0.03	1.07
WS6	0.28	0.09	0.07	0.06	0.05	0.08	0.01	0.02	0.02	0.06	0.08	0.05	0.006	0.00	0.03	1.70
WS7	0.40	0.02	0.02	0.03	0.03	0.05	0.01	0.01	0.01	0.04	0.06	0.03	0.003	0.00	0.02	0.87
WS8	0.45	0.01	0.01	0.04	0.08	1.70	No IS	No IS	No IS	No IS	No IS	0.08	0.003	0.07	0.06	0.48
WS9	0.92	0.04	0.01	0.03	0.03	0.09	0.02	0.09	0.80	2.39	3.42	0.03	0.003	0.00	0.03	0.56
WS10	0.62	0.05	0.01	0.02	0.02	0.03	0.00	0.00	0.01	0.02	0.02	0.02	0.004	0.00	0.01	0.44
WS11	0.18	0.07	0.03	0.05	0.04	0.07	0.01	0.01	0.02	0.06	0.08	0.04	0.004	0.00	0.03	1.88
WS12	0.69	0.17	0.06	0.07	0.08	0.12	0.02	0.02	0.03	0.08	0.12	0.08	0.006	0.00	0.05	4.61
WS13	0.20	0.04	0.02	0.04	0.07	0.61	No IS	No IS	No IS	No IS	No IS	0.08	0.003	0.04	0.05	0.52
WS14	0.27	0.01	0.01	0.02	0.02	0.03	0.00	0.01	0.01	0.02	0.03	0.02	0.003	0.00	0.02	0.85
DS1	4.60	0.31	0.12	0.15	0.11	0.20	0.03	0.02	0.02	0.05	0.07	0.10	0.021	0.00	0.04	2.53
DS2	0.57	0.11	0.03	0.03	0.02	0.03	0.00	0.00	0.00	0.01	0.02	0.07	0.023	0.00	0.03	0.85
DS3	0.42	0.10	0.03	0.04	0.03	0.04	0.00	0.00	0.00	0.01	0.02	0.11	0.021	0.00	0.05	0.98
DS4	0.11	0.03	0.01	0.01	0.01	0.01	0.00	0.00	0.00	0.01	0.01	0.04	0.181	0.00	0.03	0.30
DS5	2.57	0.30	0.09	0.16	0.10	0.15	0.02	0.01	0.01	0.04	0.05	0.12	0.022	0.00	0.05	3.42
DS6	1.05	0.11	0.04	0.07	0.05	0.09	0.01	0.02	0.02	0.06	0.08	0.08	0.007	0.00	0.04	2.33
DS7	1.69	0.22	0.08	0.14	0.09	0.15	0.02	0.02	0.01	0.04	0.06	0.12	0.023	0.00	0.05	3.40
DS8	0.49	0.42	0.15	0.19	0.15	0.22	0.03	0.03	0.02	0.07	0.11	0.14	0.023	0.00	0.05	5.17
DS9	1.57	0.34	0.10	0.13	0.09	0.11	0.01	0.01	0.01	0.03	0.04	0.18	0.036	0.00	0.06	5.08
DS10	0.21	0.06	0.02	0.02	0.02	0.02	0.00	0.00	0.00	0.01	0.01	0.06	0.021	0.00	0.02	0.66
DS11	1.17	0.14	No IS	0.08	0.06	0.10	0.01	0.02	0.02	0.07	0.10	0.09	0.007	0.00	0.05	4.35
DS12	1.60	0.30	0.09	0.14	0.10	0.17	0.02	0.03	0.06	0.17	0.25	0.14	0.020	0.00	0.06	4.84

DS13	0.66	0.10	0.04	0.05	0.04	0.08	0.01	0.02	0.07	0.21	0.31	0.09	0.012	0.00	0.04	0.78

Table S6. Method Quantification Limits (MQLs; pg m^{-3}). “No IS” indicates that the MQL could not be determined because the internal standard was not observable.

Sample	PFBA	PFPeA	PFHxA	PFHpA	PFOA	PFNA	PFDA	PFUnDA	PFDoDA	PFTriDA	PFTeDA	PFBS	PFHxS	PFOS	6:2 FTS	HFPO-DA
NL1	0.87	0.33	0.50	0.15	0.22	0.40	0.43	No IS	No IS	No IS	No IS	0.22	0.04	0.07	0.16	4.05
NL2	0.62	0.21	0.16	0.08	0.15	0.47	0.44	No IS	No IS	No IS	No IS	0.10	0.03	0.05	0.08	1.32
NL3	2.64	0.18	0.12	0.08	0.16	0.56	1.42	No IS	No IS	No IS	No IS	0.09	0.02	0.06	0.08	0.84
NL4	0.24	0.18	0.13	0.05	0.10	0.07	0.02	0.05	0.20	0.90	1.07	0.11	0.03	0.01	0.07	1.05
NL5	1.03	0.25	0.31	0.09	0.12	0.19	0.13	3.54	No IS	No IS	No IS	0.12	0.03	0.03	0.09	1.35
NL6	1.03	0.42	0.38	0.18	0.20	0.26	0.10	0.34	1.25	5.51	6.54	0.14	0.03	0.01	0.13	3.87
NL7	1.01	0.38	0.31	0.14	0.15	0.17	0.05	0.13	0.26	1.16	1.38	0.12	0.03	0.01	0.10	2.42
NL8	1.03	0.42	0.26	0.20	0.38	1.28	3.46	No IS	No IS	No IS	No IS	0.23	0.04	0.24	0.16	2.34
NL9	1.04	0.27	0.16	0.10	0.12	0.20	0.14	1.82	No IS	No IS	No IS	0.12	0.03	0.02	0.09	1.29
NL10	1.03	0.57	0.53	0.17	0.22	0.45	0.25	1.17	No IS	No IS	No IS	0.16	0.03	0.03	0.13	4.70
NL11	0.70	0.11	0.09	0.04	0.08	0.20	0.46	No IS	No IS	No IS	No IS	0.07	0.02	0.05	0.05	0.58
NL12	0.89	0.21	0.26	0.07	0.13	0.21	0.17	1.65	No IS	No IS	No IS	0.09	0.03	0.02	0.08	1.20
NL13	4.31	0.48	0.44	0.20	0.32	0.67	1.25	No IS	No IS	No IS	No IS	0.25	0.04	0.09	0.23	3.29
NL14	2.44	0.35	0.29	0.14	0.17	0.23	0.11	0.44	2.99	13.13	15.60	0.11	0.03	0.01	0.11	1.97
NL15	1.05	0.18	0.16	0.08	0.09	0.16	0.07	0.41	No IS	No IS	No IS	0.07	0.01	0.01	0.06	1.22
NL16	0.48	0.17	0.14	0.05	0.05	0.06	0.02	0.10	0.51	2.24	2.66	0.04	0.03	0.01	0.04	0.57
WS1	1.05	0.15	0.31	0.17	0.53	No IS	No IS	No IS	No IS	No IS	No IS	0.18	0.01	0.19	0.15	1.48
WS2	1.06	0.16	0.35	0.10	0.13	0.20	0.06	0.15	0.56	2.46	2.93	0.04	0.01	0.01	0.07	2.16
WS3	0.80	0.17	0.15	0.10	0.14	0.12	0.03	0.03	0.04	0.16	0.19	0.05	0.01	0.00	0.08	1.81
WS4	0.31	0.14	0.16	0.08	0.10	0.16	0.05	0.17	0.24	1.07	1.27	0.03	0.01	0.01	0.06	1.29
WS5	0.94	0.14	0.29	0.12	0.17	0.25	0.06	0.12	0.69	3.03	3.60	0.06	0.01	0.01	0.09	2.99
WS6	0.65	0.29	0.88	0.20	0.19	0.20	0.04	0.05	0.05	0.21	0.25	0.06	0.01	0.00	0.09	4.75
WS7	0.91	0.05	0.20	0.09	0.11	0.13	0.02	0.03	0.03	0.15	0.17	0.04	0.01	0.00	0.06	2.43
WS8	1.03	0.04	0.15	0.13	0.32	4.20	No IS	No IS	No IS	No IS	No IS	0.11	0.01	0.20	0.17	1.34
WS9	2.12	0.14	0.16	0.10	0.12	0.21	0.08	0.29	2.03	8.92	10.60	0.04	0.01	0.01	0.07	1.55
WS10	1.43	0.15	0.15	0.08	0.08	0.07	0.01	0.02	0.01	0.06	0.07	0.03	0.01	0.00	0.04	1.23
WS11	0.41	0.23	0.32	0.17	0.18	0.16	0.03	0.04	0.05	0.21	0.25	0.06	0.01	0.00	0.09	5.26
WS12	1.58	0.55	0.68	0.25	0.33	0.30	0.06	0.07	0.07	0.30	0.36	0.11	0.01	0.01	0.13	12.87
WS13	0.47	0.13	0.29	0.12	0.30	1.51	No IS	No IS	No IS	No IS	No IS	0.10	0.01	0.11	0.13	1.46
WS14	0.62	0.04	0.14	0.07	0.07	0.07	0.01	0.02	0.02	0.08	0.09	0.02	0.00	0.00	0.04	2.38
DS1	10.55	0.98	1.49	0.52	0.48	0.50	0.09	0.06	0.04	0.17	0.20	0.14	0.03	0.01	0.12	7.08
DS2	1.31	0.36	0.30	0.11	0.10	0.08	0.01	0.01	0.01	0.05	0.05	0.10	0.03	0.01	0.08	2.39
DS3	0.97	0.33	0.34	0.14	0.12	0.10	0.02	0.01	0.01	0.05	0.05	0.15	0.03	0.01	0.14	2.76
DS4	0.25	0.08	0.06	0.03	0.03	0.03	0.01	0.01	0.01	0.02	0.03	0.06	0.03	0.00	0.08	0.83
DS5	5.91	0.96	1.09	0.56	0.44	0.38	0.05	0.05	0.03	0.13	0.16	0.16	0.03	0.01	0.14	9.57
DS6	2.42	0.34	0.45	0.23	0.21	0.22	0.04	0.05	0.05	0.21	0.25	0.11	0.01	0.01	0.10	6.50
DS7	3.87	0.69	1.00	0.49	0.40	0.36	0.05	0.06	0.04	0.16	0.18	0.17	0.04	0.01	0.15	9.51
DS8	1.13	1.33	1.84	0.66	0.62	0.56	0.10	0.10	0.06	0.27	0.33	0.20	0.04	0.01	0.15	14.46
DS9	3.62	1.07	1.15	0.46	0.37	0.28	0.04	0.04	0.02	0.10	0.12	0.24	0.05	0.01	0.17	14.21
DS10	0.48	0.18	0.18	0.07	0.07	0.06	0.01	0.01	0.01	0.03	0.04	0.09	0.03	0.01	0.07	1.84
DS11	2.69	0.44	No IS	0.28	0.26	0.25	0.04	0.06	0.06	0.25	0.30	0.13	0.02	0.01	0.12	12.16
DS12	3.67	0.96	1.11	0.49	0.43	0.41	0.08	0.10	0.15	0.65	0.77	0.19	0.03	0.01	0.17	13.52

DS13	1.52	0.33	0.41	0.18	0.18	0.19	0.05	0.08	0.18	0.80	0.95	0.13	0.02	0.01	0.11	0.73

Table S7. Sample concentrations (pg m^{-3}). Values below Method Quantification Limits (MQLs) are in *italics*. Values below method detection limits (MDLs) are listed as "<MDL). "No IS" indicates that the concentration could not be determined because the internal standard was not observable.

Sample	PFBA	PFPeA	PFHxA	PFHpA	PFOA	PFNA	PFDA	PFUnDA	PFDoDA	PFTriDA	PFTeDA	PFBS	PFHxS	PFOS	6:2 FTS	HFPO-DA
NL1	<MDL	<MDL	*0.21*	0.21	2.76	<MDL	<MDL	No IS	No IS	No IS	No IS	<MDL	0.06	0.50	0.71	27.35
NL2	<MDL	<MDL	0.24	0.22	0.60	<MDL	*0.15*	No IS	No IS	No IS	No IS	<MDL	*0.02*	0.27	0.37	<MDL
NL3	<MDL	<MDL	0.23	0.33	2.24	*0.33*	<MDL	No IS	No IS	No IS	No IS	<MDL	0.04	0.37	0.28	*0.31*
NL4	9.25	0.36	0.57	0.26	0.74	0.14	0.34	*0.02*	<MDL	<MDL	<MDL	*0.08*	*0.01*	0.12	0.30	<MDL
NL5	<MDL	<MDL	*0.13*	0.18	1.62	*0.15*	*0.12*	<MDL	No IS	No IS	No IS	<MDL	*0.01*	0.11	0.27	<MDL
NL6	<MDL	<MDL	*0.11*	*0.12*	0.57	*0.11*	0.27	<MDL	<MDL	<MDL	<MDL	<MDL	0.04	0.25	0.41	<MDL
NL7	<MDL	<MDL	*0.10*	0.19	4.02	0.19	0.17	<MDL	<MDL	<MDL	<MDL	<MDL	0.09	0.27	0.48	42.60
NL8	<MDL	<MDL	0.40	0.27	1.56	<MDL	<MDL	No IS	No IS	No IS	No IS	<MDL	*0.03*	*0.09*	0.28	98.66
NL9	<MDL	<MDL	0.23	0.29	2.38	*0.17*	*0.05*	<MDL	No IS	No IS	No IS	<MDL	0.05	0.26	0.16	3.90
NL10	<MDL	<MDL	*0.05*	0.22	0.93	*0.23*	0.38	<MDL	No IS	No IS	No IS	<MDL	0.09	0.61	0.66	4.79
NL11	<MDL	<MDL	*0.03*	0.06	0.53	<MDL	<MDL	No IS	No IS	No IS	No IS	<MDL	0.02	0.10	0.18	<MDL
NL12	<MDL	<MDL	*0.04*	*0.06*	0.35	*0.09*	*0.12*	<MDL	No IS	No IS	No IS	<MDL	*0.02*	0.18	0.64	<MDL
NL13	<MDL	<MDL	<MDL	*0.08*	1.15	<MDL	<MDL	No IS	No IS	No IS	No IS	<MDL	0.05	0.29	1.40	<MDL
NL14	<MDL	<MDL	0.48	0.33	1.04	0.26	0.60	<MDL	<MDL	<MDL	<MDL	0.20	0.04	0.38	0.55	3.87
NL15	<MDL	<MDL	*0.12*	0.18	2.07	0.25	0.25	<MDL	No IS	No IS	No IS	<MDL	0.06	0.63	0.33	*0.89*
NL16	<MDL	<MDL	0.17	0.16	0.35	0.17	0.07	<MDL	<MDL	<MDL	<MDL	<MDL	0.04	0.20	0.12	0.57
WS1	<MDL	<MDL	<MDL	<MDL	0.91	No IS	No IS	No IS	No IS	No IS	No IS	0.42	0.07	1.71	0.36	<MDL
WS2	<MDL	<MDL	<MDL	<MDL	0.27	*0.12*	0.10	*0.10*	<MDL	<MDL	<MDL	0.05	0.05	0.36	0.46	*0.86*
WS3	<MDL	<MDL	*0.04*	*0.07*	0.67	0.17	0.14	0.07	0.07	<MDL	<MDL	0.12	0.04	0.25	0.38	<MDL
WS4	<MDL	<MDL	*0.03*	*0.04*	0.15	*0.09*	0.07	<MDL	<MDL	<MDL	<MDL	<MDL	0.04	0.34	0.22	<MDL
WS5	<MDL	<MDL	<MDL	<MDL	0.63	<MDL	0.14	<MDL	<MDL	<MDL	<MDL	0.07	0.05	0.49	0.96	*1.07*
WS6	<MDL	<MDL	<MDL	<MDL	0.36	*0.09*	0.13	*0.05*	*0.04*	<MDL	<MDL	0.12	0.06	0.38	0.84	<MDL
WS7	<MDL	<MDL	<MDL	*0.05*	0.59	0.14	0.17	0.18	0.20	*0.13*	<MDL	0.13	0.07	0.41	0.44	*2.17*
WS8	<MDL	<MDL	*0.06*	*0.09*	*0.32*	<MDL	No IS	No IS	No IS	No IS	No IS	0.11	0.11	2.33	1.09	1.64
WS9	<MDL	<MDL	<MDL	<MDL	0.24	<MDL	*0.07*	<MDL	<MDL	<MDL	<MDL	0.06	0.05	0.45	0.33	<MDL
WS10	<MDL	<MDL	*0.05*	0.11	0.31	0.17	0.08	0.03	*0.01*	<MDL	<MDL	<MDL	0.07	0.31	0.10	<MDL
WS11	<MDL	<MDL	<MDL	<MDL	0.22	<MDL	0.07	<MDL	<MDL	<MDL	<MDL	<MDL	0.03	0.17	0.42	<MDL
WS12	<MDL	<MDL	<MDL	<MDL	0.58	<MDL	0.15	0.12	0.10	<MDL	<MDL	0.18	0.06	0.37	1.19	<MDL
WS13	<MDL	<MDL	<MDL	<MDL	*0.26*	<MDL	No IS	No IS	No IS	No IS	No IS	0.17	0.09	1.35	0.55	*0.73*
WS14	<MDL	<MDL	*0.02*	*0.04*	0.23	*0.07*	0.08	0.03	0.03	<MDL	<MDL	0.05	0.06	0.42	0.22	*1.26*
DS1	<MDL	<MDL	<MDL	<MDL	*0.24*	<MDL	*0.03*	<MDL	<MDL	<MDL	<MDL	<MDL	*0.03*	0.19	0.27	<MDL
DS2	<MDL	<MDL	*0.07*	*0.07*	0.29	0.08	0.07	0.02	0.03	<MDL	<MDL	<MDL	0.06	0.45	0.17	<MDL
DS3	<MDL	<MDL	*0.05*	*0.05*	0.24	*0.06*	0.02	*0.01*	*0.01*	<MDL	<MDL	<MDL	0.04	0.32	0.20	<MDL
DS4	<MDL	<MDL	*0.01*	*0.01*	*0.02*	<MDL	*0.00*	<MDL	<MDL	<MDL	<MDL	<MDL	*0.01*	0.08	0.11	<MDL
DS5	<MDL	<MDL	<MDL	<MDL	*0.22*	<MDL	<MDL	<MDL	<MDL	<MDL	<MDL	<MDL	0.06	0.37	0.30	<MDL
DS6	<MDL	<MDL	<MDL	<MDL	0.26	<MDL	*0.02*	<MDL	<MDL	<MDL	<MDL	<MDL	0.07	0.35	0.46	*3.39*
DS7	<MDL	<MDL	*0.09*	*0.29*	0.58	0.39	0.52	0.52	0.65	0.84	0.22	<MDL	*0.03*	0.23	0.70	<MDL
DS8	<MDL	<MDL	<MDL	<MDL	*0.22*	<MDL	<MDL	<MDL	<MDL	<MDL	<MDL	<MDL	0.07	0.39	0.52	<MDL
DS9	<MDL	<MDL	<MDL	<MDL	0.74	*0.12*	0.11	*0.03*	0.06	<MDL	<MDL	<MDL	0.13	0.95	0.46	<MDL
DS10	*0.27*	<MDL	*0.05*	*0.07*	0.35	0.09	0.04	*0.00*	<MDL	<MDL	<MDL	<MDL	0.11	0.60	0.16	<MDL
DS11	<MDL	<MDL	No IS	<MDL	*0.21*	<MDL	*0.04*	<MDL	<MDL	<MDL	<MDL	<MDL	0.07	0.32	0.36	<MDL

DS12	<MDL	<MDL	<MDL	<MDL	*0.38*	<MDL	<MDL	<MDL	<MDL	<MDL	<MDL	<MDL	0.10	0.59	0.64	*12.21*
DS13	<MDL	<MDL	*0.04*	*0.09*	0.34	*0.10*	0.10	*0.05*	<MDL	<MDL	<MDL	<MDL	0.04	0.23	0.55	7.94

Table S8. Sample concentrations (pg m^{-3}). Values above method detection limits (MDLs) were corrected by subtracting MDL/2 values shown in Table S4

Sample	PFBA	PFPeA	PFHxA	PFHpA	PFOA	PFNA	PFDA	PFUnDA	PFDoDA	PFTriDA	PFTeDA	PFBS	PFHxS	PFOS	6:2 FTS	HFPO-DA
NL1	<MDL	<MDL	0.20	0.21	2.76	<MDL	<MDL	<MDL	<MDL	<MDL	<MDL	<MDL	0.06	0.49	0.70	27.23
NL2	<MDL	<MDL	0.24	0.21	0.60	<MDL	0.15	<MDL	<MDL	<MDL	<MDL	<MDL	0.02	0.27	0.36	<MDL
NL3	<MDL	<MDL	0.23	0.33	2.24	0.32	<MDL	<MDL	<MDL	<MDL	<MDL	<MDL	0.04	0.36	0.27	0.19
NL4	8.94	0.36	0.57	0.25	0.74	0.13	0.33	0.02	<MDL	<MDL	<MDL	0.06	0.01	0.12	0.30	<MDL
NL5	<MDL	<MDL	0.13	0.18	1.61	0.15	0.12	<MDL	<MDL	<MDL	<MDL	<MDL	0.01	0.11	0.26	<MDL
NL6	<MDL	<MDL	0.10	0.12	0.57	0.10	0.27	<MDL	<MDL	<MDL	<MDL	<MDL	0.04	0.25	0.40	<MDL
NL7	<MDL	<MDL	0.10	0.18	4.02	0.19	0.17	<MDL	<MDL	<MDL	<MDL	<MDL	0.08	0.27	0.48	42.48
NL8	<MDL	<MDL	0.40	0.26	1.56	<MDL	<MDL	<MDL	<MDL	<MDL	<MDL	<MDL	0.03	0.09	0.27	98.54
NL9	<MDL	<MDL	0.22	0.29	2.38	0.16	0.05	<MDL	<MDL	<MDL	<MDL	<MDL	0.05	0.26	0.15	3.78
NL10	<MDL	<MDL	0.05	0.21	0.93	0.22	0.38	<MDL	<MDL	<MDL	<MDL	<MDL	0.09	0.61	0.65	4.67
NL11	<MDL	<MDL	0.03	0.05	0.53	<MDL	<MDL	<MDL	<MDL	<MDL	<MDL	<MDL	0.02	0.10	0.17	<MDL
NL12	<MDL	<MDL	0.04	0.05	0.35	0.08	0.11	<MDL	<MDL	<MDL	<MDL	<MDL	0.02	0.18	0.64	<MDL
NL13	<MDL	<MDL	<MDL	0.07	1.15	<MDL	<MDL	<MDL	<MDL	<MDL	<MDL	<MDL	0.05	0.29	1.40	<MDL
NL14	<MDL	<MDL	0.48	0.33	1.04	0.25	0.60	<MDL	<MDL	<MDL	<MDL	0.17	0.04	0.38	0.55	3.75
NL15	<MDL	<MDL	0.12	0.17	2.07	0.25	0.24	<MDL	<MDL	<MDL	<MDL	<MDL	0.06	0.63	0.32	0.77
NL16	<MDL	<MDL	0.17	0.15	0.35	0.16	0.07	<MDL	<MDL	<MDL	<MDL	<MDL	0.04	0.20	0.11	0.45
WS1	<MDL	<MDL	<MDL	<MDL	0.91	<MDL	<MDL	<MDL	<MDL	<MDL	<MDL	0.39	0.07	1.71	0.35	<MDL
WS2	<MDL	<MDL	<MDL	<MDL	0.27	0.11	0.10	0.10	<MDL	<MDL	<MDL	0.02	0.04	0.36	0.45	0.74
WS3	<MDL	<MDL	0.04	0.06	0.67	0.16	0.14	0.07	0.07	<MDL	<MDL	0.09	0.04	0.25	0.38	<MDL
WS4	<MDL	<MDL	0.03	0.03	0.15	0.08	0.07	<MDL	<MDL	<MDL	<MDL	<MDL	0.04	0.34	0.21	<MDL
WS5	<MDL	<MDL	<MDL	<MDL	0.63	<MDL	0.14	<MDL	<MDL	<MDL	<MDL	0.05	0.05	0.49	0.95	0.95
WS6	<MDL	<MDL	<MDL	<MDL	0.36	0.08	0.12	0.05	0.04	<MDL	<MDL	0.09	0.06	0.38	0.83	<MDL
WS7	<MDL	<MDL	<MDL	0.05	0.59	0.13	0.16	0.18	0.20	0.13	<MDL	0.10	0.06	0.41	0.43	2.05
WS8	<MDL	<MDL	0.06	0.09	0.32	<MDL	<MDL	<MDL	<MDL	<MDL	<MDL	0.09	0.10	2.33	1.08	1.52
WS9	<MDL	<MDL	<MDL	<MDL	0.24	<MDL	0.07	<MDL	<MDL	<MDL	<MDL	0.03	0.05	0.45	0.32	<MDL
WS10	<MDL	<MDL	0.05	0.10	0.31	0.16	0.07	0.03	0.00	<MDL	<MDL	<MDL	0.07	0.31	0.09	<MDL
WS11	<MDL	<MDL	<MDL	<MDL	0.22	<MDL	0.07	<MDL	<MDL	<MDL	<MDL	<MDL	0.03	0.17	0.41	<MDL
WS12	<MDL	<MDL	<MDL	<MDL	0.58	<MDL	0.15	0.12	0.10	<MDL	<MDL	0.15	0.06	0.36	1.19	<MDL
WS13	<MDL	<MDL	<MDL	<MDL	0.26	<MDL	<MDL	<MDL	<MDL	<MDL	<MDL	0.15	0.09	1.35	0.54	0.61
WS14	<MDL	<MDL	0.02	0.04	0.23	0.06	0.07	0.02	0.03	<MDL	<MDL	0.03	0.06	0.42	0.21	1.14
DS1	<MDL	<MDL	<MDL	<MDL	0.24	<MDL	0.02	<MDL	<MDL	<MDL	<MDL	<MDL	0.03	0.19	0.26	<MDL
DS2	<MDL	<MDL	0.07	0.07	0.29	0.07	0.07	0.02	0.03	<MDL	<MDL	<MDL	0.06	0.44	0.17	<MDL
DS3	<MDL	<MDL	0.05	0.04	0.24	0.05	0.02	0.00	0.00	<MDL	<MDL	<MDL	0.04	0.32	0.19	<MDL
DS4	<MDL	<MDL	0.12	0.06	0.18	<MDL	0.03	<MDL	<MDL	<MDL	<MDL	<MDL	0.08	0.64	0.86	<MDL
DS5	<MDL	<MDL	<MDL	<MDL	0.22	<MDL	<MDL	<MDL	<MDL	<MDL	<MDL	<MDL	0.06	0.36	0.29	<MDL
DS6	<MDL	<MDL	<MDL	<MDL	0.26	<MDL	0.02	<MDL	<MDL	<MDL	<MDL	<MDL	0.07	0.35	0.45	3.27
DS7	<MDL	<MDL	0.09	0.28	0.58	0.38	0.52	0.52	0.65	0.83	0.21	<MDL	0.03	0.23	0.69	<MDL
DS8	<MDL	<MDL	<MDL	<MDL	0.22	<MDL	<MDL	<MDL	<MDL	<MDL	<MDL	<MDL	0.07	0.39	0.51	<MDL
DS9	<MDL	<MDL	<MDL	<MDL	0.74	0.12	0.11	0.03	0.05	<MDL	<MDL	<MDL	0.13	0.95	0.46	<MDL
DS10	<MDL	<MDL	0.05	0.07	0.35	0.09	0.04	0.00	<MDL	<MDL	<MDL	<MDL	0.11	0.60	0.15	<MDL
DS11	<MDL	<MDL	<MDL	<MDL	0.21	<MDL	0.03	<MDL	<MDL	<MDL	<MDL	<MDL	0.07	0.32	0.35	<MDL
DS12	<MDL	<MDL	<MDL	<MDL	0.38	<MDL	<MDL	<MDL	<MDL	<MDL	<MDL	<MDL	0.10	0.59	0.63	12.09

DS13	<MDL	<MDL	0.04	0.08	0.34	0.09	0.09	0.05	<MDL	<MDL	<MDL	<MDL	0.04	0.23	0.55	7.82

Table S9. Relative recoveries of IS per sample (%).

Sample	PFBA	PFPeA	PFHxA	PFHpA	PFOA	PFNA	PFDA	PFUnDA	PFDoDA	PFTriDA	PFTeDA	PFBS	PFHxS	PFOS	6:2 FTS	HFPO-DA
NL1	71.44	63.38	24.80	51.98	37.88	21.12	3.41	No IS	No IS	No IS	No IS	32.89	32.89	6.30	38.77	16.92
NL2	62.81	61.58	50.03	58.32	35.50	11.44	2.11	No IS	No IS	No IS	No IS	203.18	203.18	24.79	242.98	32.58
NL3	9.03	44.21	41.10	36.34	19.35	5.81	0.40	No IS	No IS	No IS	No IS	40.39	40.39	3.94	39.56	31.37
NL4	131.84	59.25	50.07	77.40	43.44	61.05	39.27	15.34	2.57	2.57	2.57	31.34	31.34	33.73	44.48	33.28
NL5	28.48	40.28	19.02	40.03	32.85	21.32	5.29	0.20	No IS	No IS	No IS	35.37	35.37	9.58	45.93	24.11
NL6	50.46	41.87	27.09	34.56	34.17	27.57	11.86	3.73	0.69	0.69	0.69	42.62	42.62	29.09	42.09	14.81
NL7	33.34	30.44	21.88	30.24	30.61	27.79	16.41	6.37	2.12	2.12	2.12	54.67	54.67	38.95	63.07	15.45
NL8	28.05	23.15	22.35	18.12	10.17	3.11	0.20	No IS	No IS	No IS	No IS	26.83	26.83	1.56	34.75	13.64
NL9	23.14	29.70	29.91	28.35	25.80	16.19	4.23	0.32	No IS	No IS	No IS	32.32	32.32	11.17	37.65	20.59
NL10	45.92	28.45	17.92	35.24	28.51	14.53	4.60	0.99	No IS	No IS	No IS	95.63	95.63	28.43	107.60	11.19
NL11	20.73	43.74	32.64	42.05	25.72	9.85	0.74	No IS	No IS	No IS	No IS	38.17	38.17	3.17	42.49	27.55
NL12	27.45	40.35	19.02	42.00	24.81	15.69	3.46	0.36	No IS	No IS	No IS	42.51	42.51	10.50	44.49	22.50
NL13	15.40	46.77	29.77	40.06	27.16	13.69	1.26	No IS	No IS	No IS	No IS	25.14	25.14	4.50	24.58	22.25
NL14	17.29	40.67	29.10	36.46	32.82	24.68	8.81	2.33	0.23	0.23	0.23	53.90	53.90	31.36	54.00	23.58
NL15	20.09	40.42	26.82	33.26	29.79	18.59	6.84	1.23	No IS	No IS	No IS	45.85	45.85	21.42	48.07	19.09
NL16	27.59	26.94	19.03	32.40	32.87	29.09	12.97	3.38	0.43	0.43	0.43	85.35	85.35	43.55	104.57	25.60
WS1	9.62	22.91	6.26	7.13	2.53	0.00	No IS	No IS	No IS	No IS	No IS	4.56	4.56	0.25	4.81	7.49
WS2	17.20	39.53	10.24	22.93	18.02	12.74	7.36	3.04	0.54	0.54	0.54	26.41	26.41	10.15	14.59	9.31
WS3	45.04	72.82	46.88	42.38	35.08	40.75	32.89	30.69	16.72	16.72	16.72	39.47	39.47	32.40	19.85	21.82
WS4	42.66	33.12	16.08	20.24	17.91	11.29	6.88	1.86	0.90	0.90	0.90	25.11	25.11	6.68	11.52	11.33
WS5	22.36	52.21	14.00	20.59	15.96	11.69	8.10	4.06	0.50	0.50	0.50	19.40	19.40	7.76	11.18	7.74
WS6	53.66	39.77	7.75	21.29	23.72	23.94	20.52	15.73	12.27	12.27	12.27	38.13	38.13	32.03	24.73	8.06
WS7	34.88	218.41	30.59	41.32	38.69	34.55	34.27	22.79	15.82	15.82	15.82	44.88	44.88	28.69	23.72	14.40
WS8	5.40	49.29	7.55	5.33	2.29	0.18	No IS	No IS	No IS	No IS	No IS	6.36	6.36	0.21	3.59	4.61
WS9	7.22	37.01	18.30	18.01	16.31	9.92	4.78	1.26	0.12	0.12	0.12	24.11	24.11	6.32	12.32	10.84
WS10	14.03	45.10	26.09	30.61	32.83	38.49	33.26	31.58	23.70	23.70	23.70	56.16	56.16	37.67	33.49	18.06
WS11	126.67	74.66	31.92	36.44	38.64	43.66	36.67	29.41	18.06	18.06	18.06	50.23	50.23	37.18	30.06	10.90
WS12	47.20	45.98	21.38	36.67	29.63	33.65	31.08	27.28	18.08	18.08	18.08	31.64	31.64	34.72	24.13	6.39
WS13	21.38	25.59	6.81	9.98	4.40	0.91	No IS	No IS	No IS	No IS	No IS	8.01	8.01	0.42	5.55	7.54
WS14	32.07	150.78	27.41	34.94	39.29	38.56	32.31	26.08	19.22	19.22	19.22	57.74	57.74	31.67	27.35	9.22
DS1	3.75	13.57	5.20	9.36	10.86	10.85	10.77	15.43	16.76	16.76	16.76	35.51	35.51	42.80	37.45	6.16
DS2	16.07	19.74	13.84	22.92	28.19	34.66	36.20	37.86	34.42	34.42	34.42	34.83	34.83	39.15	43.00	9.71
DS3	34.96	34.93	20.25	30.06	38.44	47.04	53.17	56.47	55.81	55.81	55.81	41.61	41.61	50.32	38.65	13.66
DS4	45.00	46.51	44.10	49.82	48.76	51.01	40.69	39.97	35.35	35.35	35.35	52.19	52.19	50.59	43.48	14.82
DS5	8.40	17.43	8.95	10.96	14.95	17.99	21.84	24.24	27.21	27.21	27.21	38.13	38.13	47.36	40.44	5.73
DS6	20.11	48.98	21.13	25.63	30.47	30.53	29.58	21.88	16.71	16.71	16.71	26.31	26.31	29.70	24.61	8.27
DS7	17.74	33.73	13.54	17.37	22.99	26.05	30.50	29.61	32.27	32.27	32.27	39.54	39.54	50.55	40.74	7.98
DS8	35.97	10.30	4.35	7.62	8.77	10.04	9.53	10.17	10.83	10.83	10.83	22.55	22.55	27.94	27.23	3.11
DS9	15.38	17.59	9.52	14.75	20.21	27.43	32.99	36.79	41.92	41.92	41.92	42.94	42.94	54.86	57.27	4.33
DS10	44.09	41.26	23.61	36.21	41.00	47.66	48.44	48.25	49.20	49.20	49.20	44.91	44.91	50.69	59.75	12.85
DS11	22.37	46.29	0.00	26.73	31.10	32.66	32.38	25.95	17.30	17.30	17.30	28.65	28.65	33.30	25.76	5.46
DS12	20.09	25.86	13.03	18.49	22.76	24.71	20.87	18.51	8.28	8.28	8.28	35.17	35.17	39.68	35.20	6.02
DS13	25.56	39.45	18.72	26.48	29.17	27.48	20.40	12.21	3.55	3.55	3.55	30.09	30.09	24.60	31.40	19.56

Table S10. Mean meteorological observations during sampling, wherein e = vapor pressure (hPa), es = saturation vapor pressure (hPa), RH = relative humidity (%), T = temperature (ºC), Precip = precipitation (mm), TotalRad= radiation (J/m^2), WS = wind speed (m/s), WD = wind direction (º), From = starting date, To = end date.

Sample	WS	WD	WD	e	es	RH	T	Precip	TotalRad	**From**	**To**
**NL1**	1.95	195	↑	16.59	26.50	62.61	21.75	0	2761	2021-06-02	2021-06-03
**NL2**	1.29	317	↘	17.55	23.42	74.92	19.69	0	2545	2021-06-03	2021-06-04
**NL3**	2.91	333	↘	17.29	20.24	85.41	16.37	0.275	954	2021-06-04	2021-06-06
**NL4**	3.18	316	↘	14.07	17.49	80.49	16.18	0	2375	2021-06-06	2021-06-07
**NL5**	2.84	12	↓	12.67	18.91	67.03	18.42	0	3006	2021-06-07	2021-06-08
**NL6**	1.57	39	↙	13.71	21.66	63.29	19.32	0	2985	2021-06-08	2021-06-09
**NL7**	1.9	290	→	15.35	23.06	66.56	18.71	0	2734	2021-06-09	2021-06-10
**NL8**	1.57	246	↗	13.26	22.35	59.31	18.24	0	2933	2021-06-10	2021-06-11
**NL9**	2.08	266	→	17.32	21.35	81.10	18.28	0	2038	2021-06-11	2021-06-12
**NL10**	3.68	275	→	15.80	21.13	74.77	14.68	0	2327	2021-06-12	2021-06-13
**NL11**	1.22	325	↘	13.35	21.00	63.60	19.41	0	7891	2021-06-13	2021-06-15
**NL12**	1.87	62	↙	13.93	23.15	60.14	19.23	0	2809	2021-06-15	2021-06-16
**NL13**	1.16	126	↖	19.00	31.27	60.76	24.55	0	2215	2021-06-16	2021-06-17
**NL14**	0.38	331	↘	21.13	29.33	72.06	23.36	9.15	3079	2021-06-17	2021-06-18
**NL15**	1.5	336	↘	17.96	21.21	84.68	18.16	22.025	3626	2021-06-18	2021-06-21
**NL16**	3.89	28	↙	12.69	16.63	76.28	14.54	0.1	1642	2021-06-21	2021-06-22
**WS1**	1.12	343	↓	15.90	19.43	81.81	16.70	14.9	12488	2021-06-22	2021-07-01
**WS2**	2.24	200	↑	16.43	20.04	82.00	17.29	38.6	10831	2021-07-01	2021-07-08
**WS3**	1.97	327	↘	17.71	21.09	83.95	18.24	8.925	10069	2021-07-08	2021-07-15
**WS4**	2.33	352	↓	16.08	21.76	73.89	18.47	1.55	16189	2021-07-15	2021-07-22
**WS5**	2.38	216	↗	16.52	20.59	80.23	18.09	32.475	15054	2021-07-22	2021-07-29
**WS6**	0.81	68	↙	14.94	19.79	75.50	16.33	0.05	4881	2021-07-29	2021-08-05
**WS7**	4.05	209	↗	15.80	19.84	79.63	17.21	28.925	11856	2021-08-05	2021-08-12
**WS8**	3.37	249	↗	16.16	19.74	81.86	17.08	8.7	8774	2021-08-12	2021-08-19

**WS9**	1.75	332	↘	16.36	19.81	82.58	17.13	10.1	12236	2021-08-19	2021-08-27
**WS10**	3.56	354	↓	15.50	18.93	81.88	16.61	1.325	7332	2021-08-27	2021-09-02
**WS11**	1.92	44	↙	14.95	19.53	76.53	16.60	0	8953	2021-09-02	2021-09-07
**WS12**	1.51	165	↖	18.32	23.72	77.25	19.99	6.75	5405	2021-09-07	2021-09-11
**WS13**	3.13	210	↗	14.90	17.97	82.90	15.52	14.775	6624	2021-09-23	2021-09-30
**WS14**	4.65	195	↑	12.93	15.09	85.70	13.04	63	5055	2021-09-30	2021-10-07
**DS1**	1.83	85	←	11.99	13.55	88.50	11.22	0	2425	2021-10-07	2021-10-09
**DS2**	2.39	297	↘	12.25	14.10	86.93	11.57	7.35	891	2021-10-09	2021-10-12
**DS3**	2.28	307	↘	10.19	12.24	83.29	9.62	0.1	642	2021-10-12	2021-10-14
**DS5**	3.95	241	↗	13.23	15.43	85.72	13.46	2.5	638	2021-10-14	2021-10-15
**DS6**	2.08	184	↑	10.17	12.51	81.26	10.18	0.075	2099	2021-10-15	2021-10-18
**DS7**	4.16	186	↑	15.37	16.57	92.72	14.68	1.45	180	2021-10-18	2021-10-19
**DS8**	6.71	207	↗	16.24	18.66	87.01	16.18	0.95	539	2021-10-19	2021-10-20
**DS9**	5.73	243	↗	12.60	14.57	86.52	13.20	18.25	458	2021-10-20	2021-10-21
**DS10**	5.31	270	→	9.00	11.55	77.92	8.99	2.9	517	2021-10-21	2021-10-22
**DS11**	2.4	178	↑	9.93	11.05	89.83	8.56	2.225	1493	2021-10-22	2021-10-25
**DS12**	4.08	203	↗	12.62	13.27	95.06	11.28	0.25	363	2021-10-25	2021-10-26
**DS13**	5.02	201	↙	12.95	14.57	88.86	12.24	0	1429	2021-10-26	2021-10-28

Table S11. Wind sectors, their range and the number (n) of samples per sector.

Sector	Degrees (º)	n
N	337.5 – 22.5	4
NE	22.5 – 67.5	4
E	67.5 – 112.5	3
SE	112.5 – 157.5	1
S	157.5 – 202.5	8
SW	202.5 – 247.5	8
W	247.5 – 292.5	5
NW	292.5 – 337.5	10
		Total: 43

Table S12. Spearman's rho values for the detected PFAS and Na^+ in air samples. Values where $p \leq 0.05$ are marked with an asterisk*, $p \leq 0.01$** and $p \leq 0.001$ ***.

r	PFBA	PFPeA	PFHxA	PFHpA	PFOA	PFNA	PFDA	PFUnDA	PFDoDA	PFTriDA	PFTeDA	PFBS	PFHxS	PFOS	6:2 FTS	HFPO-DA
**PFBA**																
**PFPeA**	0.72***															
**PFHxA**	0.16	0.27														
**PFHpA**	0.13	0.2	0.92***													
**PFOA**	0.11	0.12	0.49***	0.63***												
**PFNA**	0.06	0.12	0.49***	0.6***	0.45**											
**PFDA**	0.21	0.23	0.25	0.3*	0.28	0.63***										
**PFUnDA**	0.18	0.16	-0.15	-0.08	-0.05	0.33*	0.42**									
**PFDoDA**	0	-0.08	-0.17	-0.11	-0.03	0.26	0.34*	0.84***								
**PFTriDA**	0	-0.03	-0.04	0.14	0.08	0.29	0.31*	0.45**	0.49***							
**PFTeDA**	0	-0.02	0.1	0.24	0.04	0.27	0.25	0.32*	0.35*	0.72***						
**PFBS**	0.21	0.2	-0.25	-0.25	0.06	-0.05	0.21	0.34*	0.27	0.11	-0.1					
**PFHxS**	-0.35*	-0.26	-0.36*	-0.33*	-0.11	-0.14	-0.22	-0.02	0.11	-0.04	-0.18	0.12				
**PFOS**	-0.28	-0.22	-0.27	-0.29	-0.07	-0.16	-0.17	-0.06	0.07	-0.05	-0.16	0.33*	0.81***			
**6:2 FTS**	0.1	-0.09	-0.27	-0.14	0.13	-0.22	0.13	0.06	0.01	0.14	0.17	0.29	0.27	0.32*		
**HFPO-DA**	0	-0.12	0.23	0.31*	0.39**	0.16	-0.01	-0.13	-0.24	0.02	-0.12	0.05	0.25	0.18	0.21	
**Na**	-0.02	0.24	0.13	0.01	-0.23	0.1	-0.04	0.02	0.01	-0.16	-0.25	-0.12	0.26	0.2	-0.29	-0.09

Table S13. Estimated air concentrations (pg m^{-3}) of H-PFCAs in the samples

ID	H-PFHxA	H-PFHpA	H-PFOA	H-PFNA	H-PFDA
NL1	<MDL	<MDL	0.03	0.01	<MDL
NL2	<MDL	0.1	0.12	0.18	<MDL
NL3	0.08	0.12	0.18	0.2	0.51
NL4	0.03	0.05	0.06	0.03	0.05
NL5	0.15	0.01	0.06	0.05	0.08
NL6	0.2	0.04	0.04	0.07	0.09
NL7	0.17	0.28	0.2	0.24	0.22
NL8	0.18	0.42	0.31	0.55	1.25
NL9	0.1	0.1	0.07	0.08	0.13
NL10	0.36	<MDL	0.07	0.11	0.03
NL11	0.06	0.03	0.03	0.05	0.04
NL12	0.2	<MDL	0.04	0.03	0.04
NL13	0.16	0.03	0.03	0.03	0.1
NL14	0.17	0.07	0.09	0.08	0.15
NL15	0.08	0.05	0.06	0.05	0.06
NL16	0.09	0.02	0.03	0.03	0.03
WS1	<MDL	0.01	0.01	<MDL	<MDL
WS2	<MDL	<MDL	0.02	0.02	0.03
WS3	<MDL	0.01	0.03	0.03	<MDL
WS4	<MDL	<MDL	0.02	0.02	0.01
WS5	<MDL	0.01	0.02	0.03	0.04
WS6	<MDL	<MDL	0.03	0.03	0.06
WS7	<MDL	0.01	0.04	0.05	0.06
WS8	<MDL	0.02	0.03	<MDL	<MDL
WS9	<MDL	0.01	0.01	0.02	0.05
WS10	<MDL	0.01	0.02	0.02	0.01
WS11	<MDL	<MDL	0	0.02	0.04
WS12	<MDL	<MDL	0	0.03	0.06
WS13	<MDL	<MDL	0.01	0.02	<MDL
WS14	<MDL	<MDL	0.01	0.01	0.02
DS1	<MDL	0.03	0.1	0.09	0.09
DS2	0.09	0.05	0.03	0.03	0.03
DS3	0.06	0.01	0.01	0.02	0.01
DS5	0.34	0.02	0.02	0.04	0.06
DS6	0.05	<MDL	0.02	0.04	0.05
DS7	<MDL	<MDL	0.03	0.11	0.14
DS8	<MDL	0.02	0.04	0.06	0.08
DS9	<MDL	0.03	0.02	0.03	0.03
DS10	<MDL	0.01	0.01	0.01	0.01
DS11	<MDL	<MDL	0.03	0.02	0.02
DS12	<MDL	<MDL	0.02	0.05	0.06
DS13	<MDL	<MDL	0.02	0.05	0.06

Table S14. PFAS suspects identified through suspect screening along with information associated with their structural assignment. Suspects were assigned level 1 when a calibration standard was acquired; level 2b with presence in a homologous series and an acquired MS^2 spectrum; level 3 when they were part of a homologous series.

Class	Name	m/z	RT	Standard acquired	MS^2 (sample)	Confidence level	Formula	In-source fragment detected	Suspected origin
**H-PFCAs** 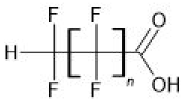	H-PFHxA	294.9816	2.25	No	No	3	$C_6H_2F_{10}O_2$	$[M-CO_2F-H]^-$	Polymerization by-product
	H-PFHpA	344.9784	3.10	No	No	3	$C_7H_2F_{12}O_2$	$[M-CO_2F-H]^-$	Polymerization by-product
	H-PFOA	394.9753	3.65	Yes	Yes	1	$C_8H_2F_{14}O_2$	$[M-CO_2F-H]^-$	Polymerization by-product
	H-PFNA	444.9721	4.02	Yes	Yes	1	$C_9H_2F_{16}O_2$	$[M-CO_2F-H]^-$	Polymerization by-product
	H-PFDA	494.9689	4.46	No	Yes	2b	$C_{10}H_2F_{18}O_2$	$[M-CO_2F-H]^-$	Polymerization by-product
	H-PFUnDA	544.9657	4.72	Yes	Yes	1	$C_{11}H_2F_{20}O_2$	-	Polymerization by-product
	H-PFDoDA	594.9625	5.02	No	No	3	$C_{12}H_2F_{22}O_2$	-	Polymerization by-product
**Fluorinated Bisphenol** 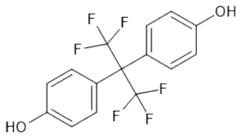	BPAF	335.0512	4.98	Yes	No	1	$C_{15}H_{10}F_6O_2$	-	Fluoroelastomer curing agent

Table S15. Modelled (FLEXPART) and measured ambient air concentrations of HFPO-DA (pg m^{-3}) in the particle phase. The greyed values show when the mixed scenario uses values from the unabated emission scenario (95 kg yr^{-1}), while the values below show the use of the abated scenario (3.2 kg yr^{-1}). Note that for the measurements values under MDL have been replaced by 0.00. The values where the modelled concentrations are >0 but <MDL and measurements are <MDL are in italics. These values represent modelled concentrations that were too low to be detected.

Sample	FLEXPART (mixed scenario)	Measurements Cabauw	MDL HFPO-DA	FLEXPART (95 kg yr^{-1})	FLEXPART (3.2 kg yr^{-1})
NL1	29.62	27.35	1.45	29.62	1.00
NL2	0.00	0.00	0.47	0.00	0.00
NL3	0.00	0.31	0.30	0.00	0.00
NL4	0.00	0.00	0.37	0.00	0.00
NL5	0.00	0.00	0.48	0.00	0.00
NL6	0.04	0.00	1.38	0.04	0.00
NL7	5.34	42.60	0.86	5.34	0.18
NL8	32.14	98.66	0.83	32.14	1.08
NL9	0.00	3.90	0.46	0.00	0.00
NL10	0.00	4.79	1.68	0.00	0.00
NL11	0.00	0.00	0.21	0.00	0.00
NL12	0.00	0.00	0.43	0.00	0.00
NL13	0.00	0.00	1.18	0.00	0.00
NL14	0.00	3.87	0.70	0.00	0.00
NL15	6.83	0.89	0.44	6.83	0.23
NL16	0.00	0.57	0.20	0.00	0.00
WS1	*0.29*	*0.00*	*0.53*	8.55	0.29
WS2	1.16	0.86	0.77	34.33	1.16
WS3	*0.02*	*0.00*	*0.65*	0.60	0.02
WS4	0.00	0.00	0.46	0.00	0.00
WS5	0.58	1.07	1.07	17.17	0.58
WS6	*0.23*	*0.00*	*1.70*	6.71	0.23
WS7	1.55	2.17	0.87	46.05	1.55
WS8	0.42	1.64	0.48	12.40	0.42
WS9	*0.03*	*0.00*	*0.56*	0.96	0.03
WS10	0.00	0.00	0.44	0.00	0.00
WS11	0.00	0.00	1.88	0.00	0.00
WS12	*0.33*	*0.00*	*4.61*	9.69	0.33
WS13	0.78	0.73	0.52	23.08	0.78
WS14	0.57	1.26	0.85	16.82	0.57
DS1	0.00	0.00	2.53	0.00	0.00
DS2	0.00	0.00	0.85	0.00	0.00
DS3	0.00	0.00	0.98	0.00	0.00
DS4	0.37	0.00	0.30	10.90	0.37
DS5	*0.81*	*0.00*	*3.42*	23.99	0.81
DS6	*0.16*	*0.00*	2.33	4.68	0.16
DS7	*1.35*	*0.00*	*3.40*	40.20	1.35
DS8	*0.53*	*0.00*	*5.17*	15.82	0.53
DS9	0.00	0.00	5.08	0.00	0.00
DS10	0.70	0.00	0.66	20.93	0.70
DS11	1.15	12.21	4.35	34.29	1.15
DS12	0.65	7.94	4.84	19.23	0.65

Table S16. Comparative metrics between ambient measured and modelled HFPO-DA concentrations at the Cabauw Meteorological Observatory

Metric	Formula	Value
Pearson's correlation coefficient (r)	$r_{xy} = \frac{\sum_{i=1}^{n}(x_i - \bar{x})(y_i - \bar{y})}{\sqrt{\sum_{i=1}^{n}(x_i - \bar{x})^2}\sqrt{\sum_{i=1}^{n}(y_i - \bar{y})^2}}$	0.83, $p<0.05$
Mean bias error (MBE)	$\frac{1}{n}\sum_{i=1}^{n}(\text{Modeled}_i - \text{Measured}_i)$	-3.06 pg m^{-3}
Mean absolute error (MAE)	$\frac{1}{n}\sum_{i=1}^{n}\lvert\text{Modeled}_i - \text{Measured}_i\rvert$	3.66 pg m^{-3}
Wilmott's index of agreement (d)	$d = 1 - \frac{\sum_{i=1}^{n}(\text{Measured}_i - \text{Modeled}_i)^2}{\sum_{i=1}^{n}\left(\lvert\text{Modeled}_i - \overline{\text{Measured}}\rvert + \lvert\text{Measured}_i - \overline{\text{Measured}}\rvert\right)^2}$	0.72

Table S17. Modeled HFPO-DA deposition ranges in ng m^{-2} $year^{-1}$ at some European cities after a one-year simulation (2020) with unabated emissions (450 kg yr^{-1}) using Chemours Dordrecht as point source and the cities' coordinates and distance from the source.

City	Longitude (º)	Latitude (º)	HFPO-DA deposition (ng m^{-2} yr^{-1})	Distance from source (km)
Dordrecht	4.65	51.85	30023.2 – 142215.3	4
Hamburg	9.95	53.55	58.8 – 278.6	481
London	-0.15	51.45	36.4 – 172.2	298
Copenhagen	12.55	55.65	13.5 – 63.8	822
Stockholm	18.05	59.35	3.5 – 16.6	1467
Reykjavik	-21.85	64.15	0.5 – 2.4	3233

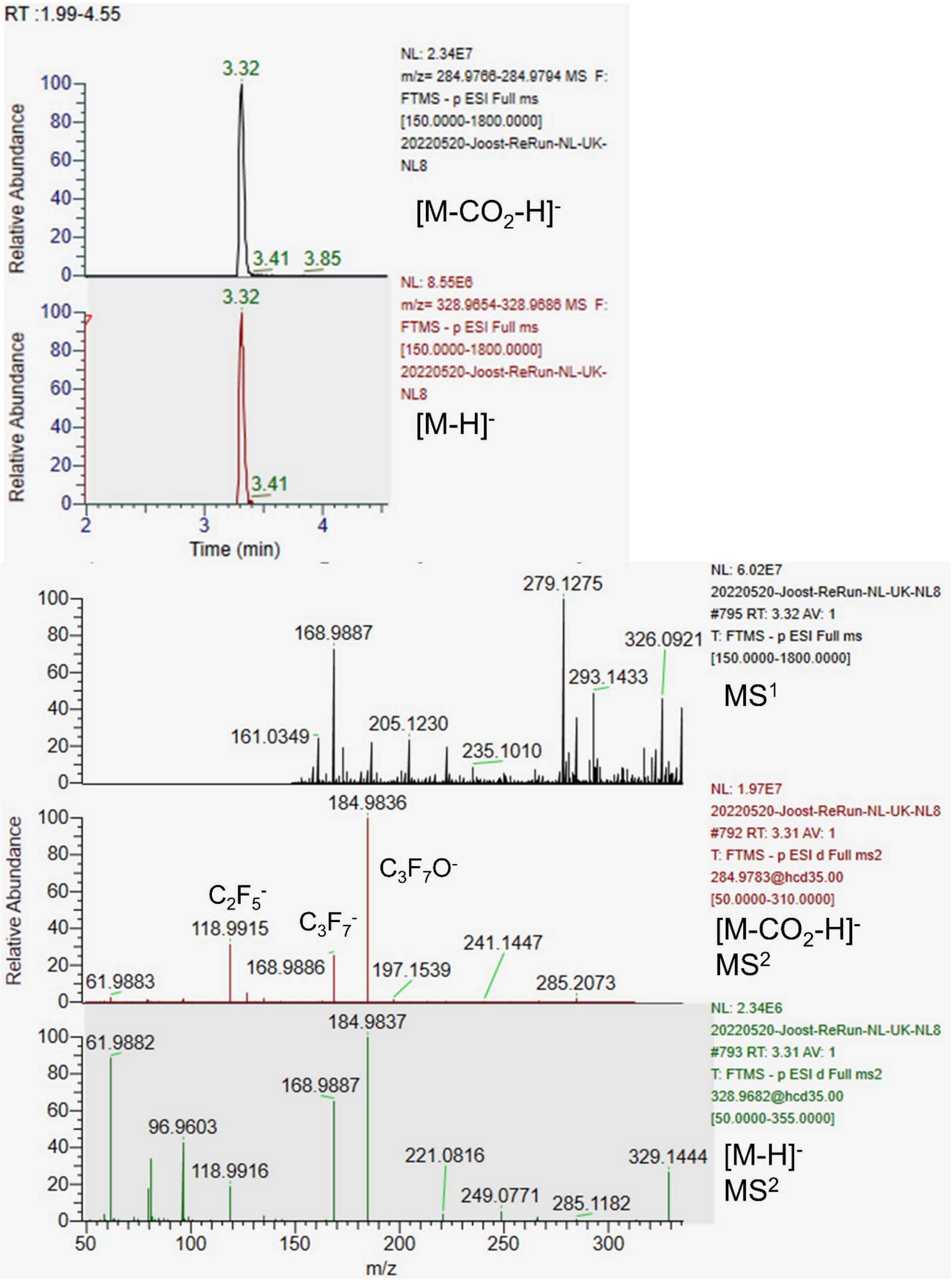

Figure S1. Area responses of both the molecular ion $[M-H]^-$ and the decarboxylated in-source fragment $[M-CO_2-H]^-$ of HFPO-DA in full-scan (MS^1) chromatogram of an air sample and MS^1 and acquired MS^2 spectra of both fragments. Note the higher response of the $[M-CO_2-H]^-$ fragment compared to the $[M-H]^-$ ion in MS^1.

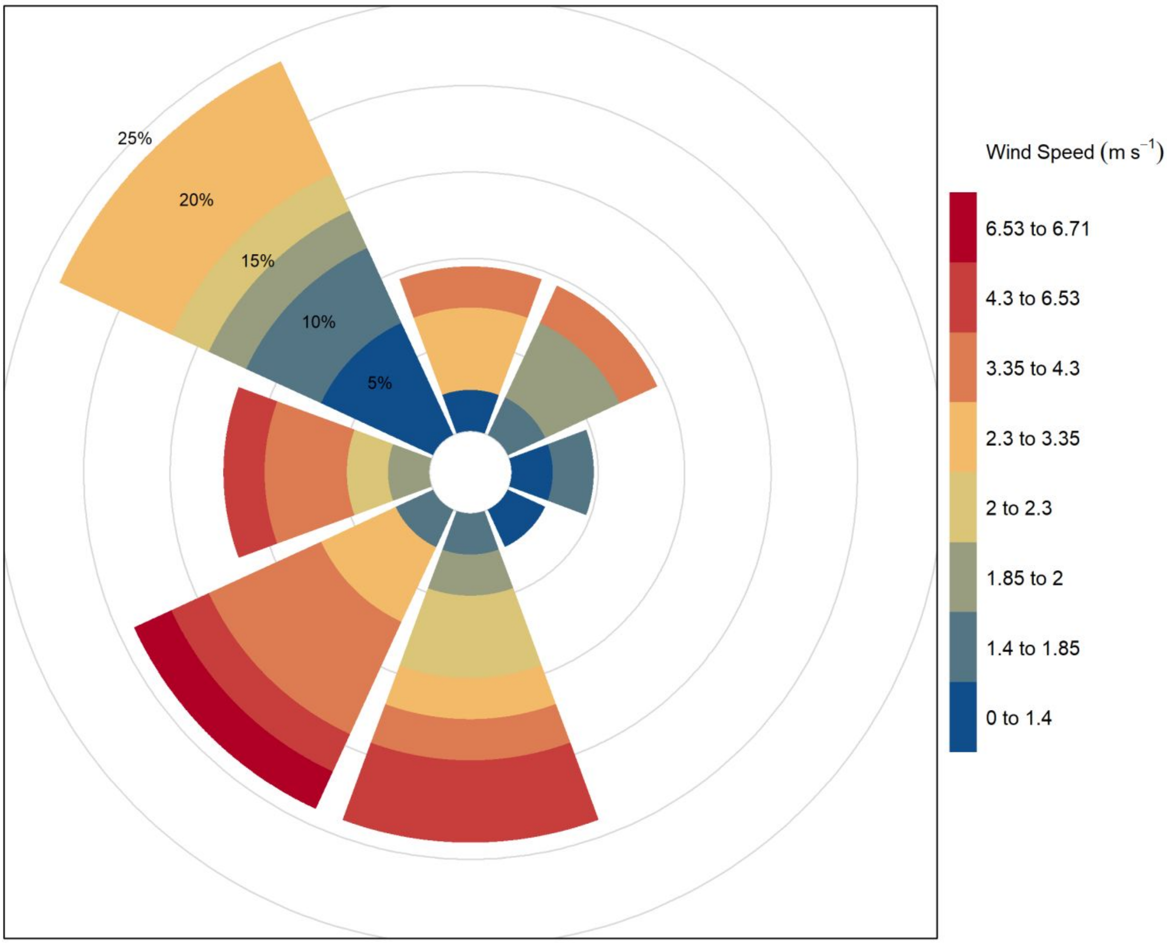

Figure S2. Wind rose of the wind speed and direction during the sampling campaign. Percentages (%) indicate the frequencies of the wind blowing from a particular direction during sampling.

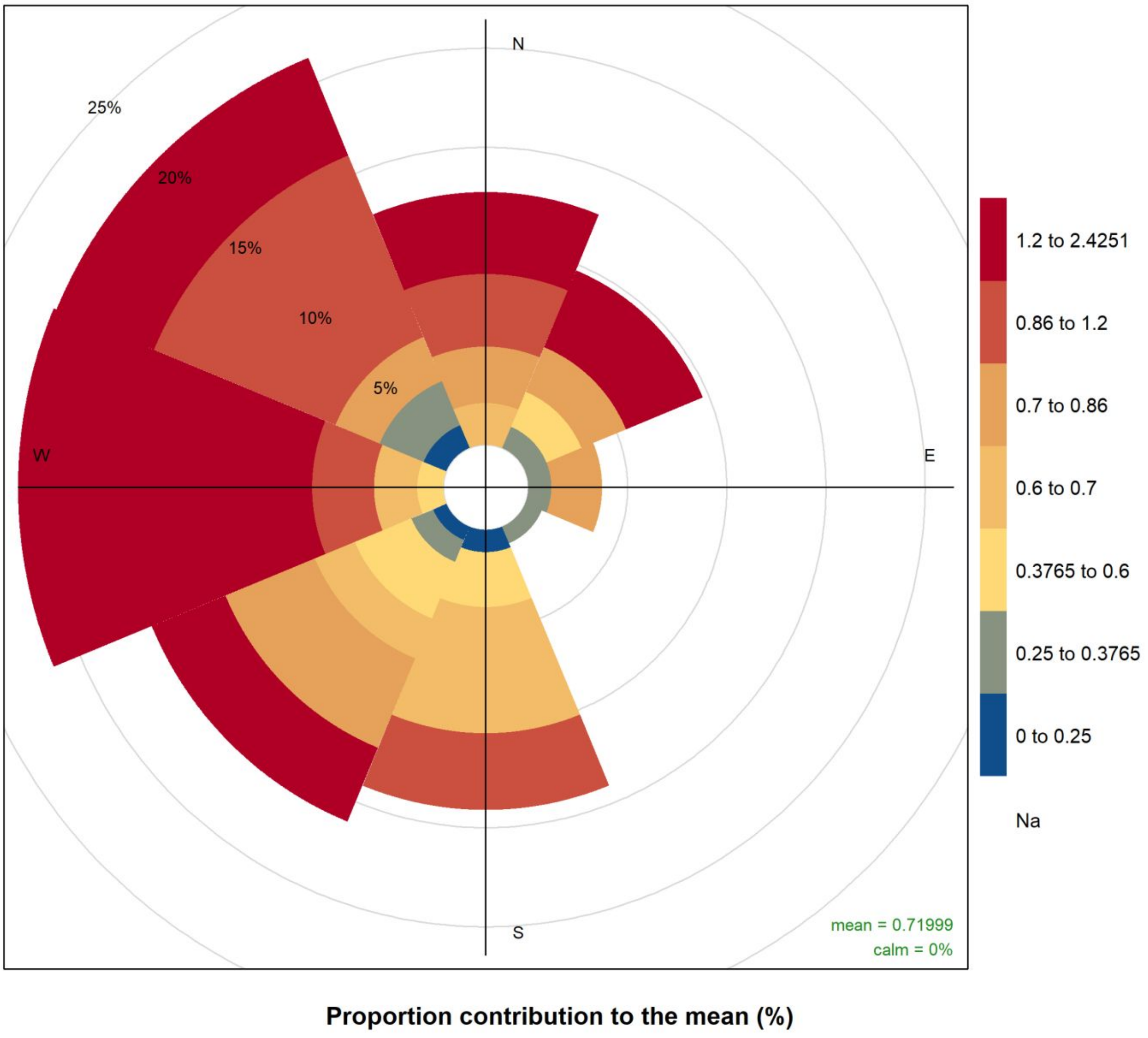

Figure S3. Pollution rose for sodium (Na^+) tracer ion with the proportion of contribution to mean concentrations shown in %. The substance specified on the lower right and concentrations are in μg m^{-3}

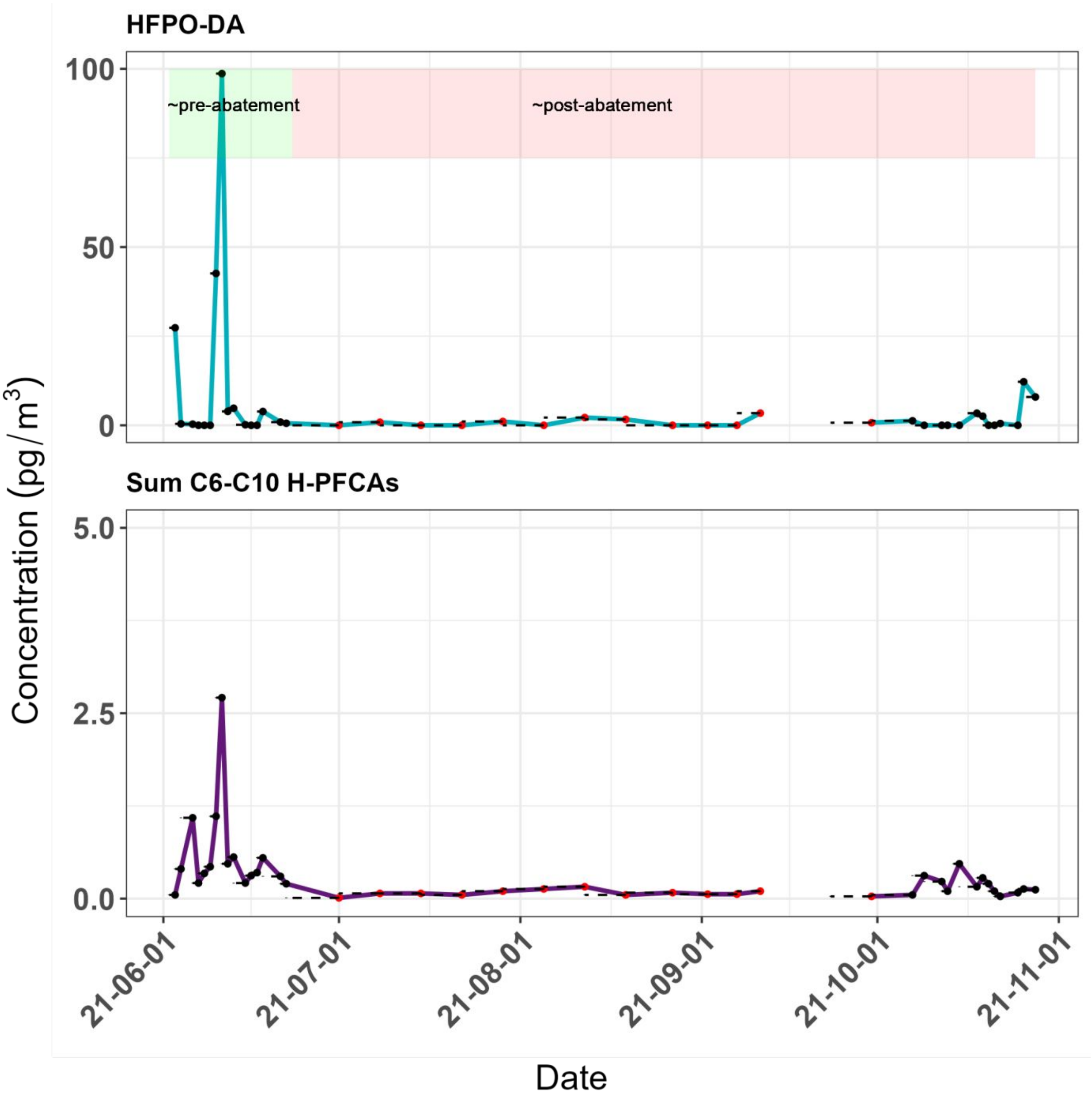

Figure S4. Comparison of the air concentration of HFPO-DA and the estimated air concentrations of H-PFCAs during the sampling campaign showing the overlapping peak in the same sample for both analytes.

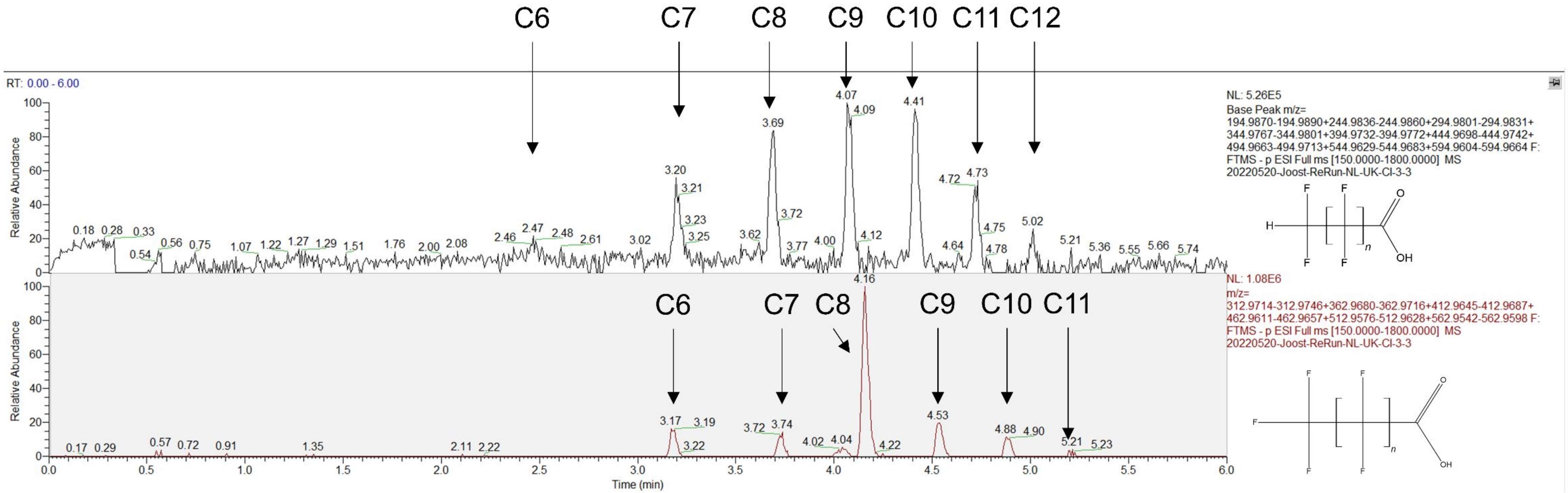

Figure S5. Chromatograms showing extracted masses from full-scan (MS[1]) data for the identification of homologous series of H-PFCAs (above) in an air sample and comparison of retention times with PFCAs (below). C8 refers to H-PFOA and PFOA respectively.

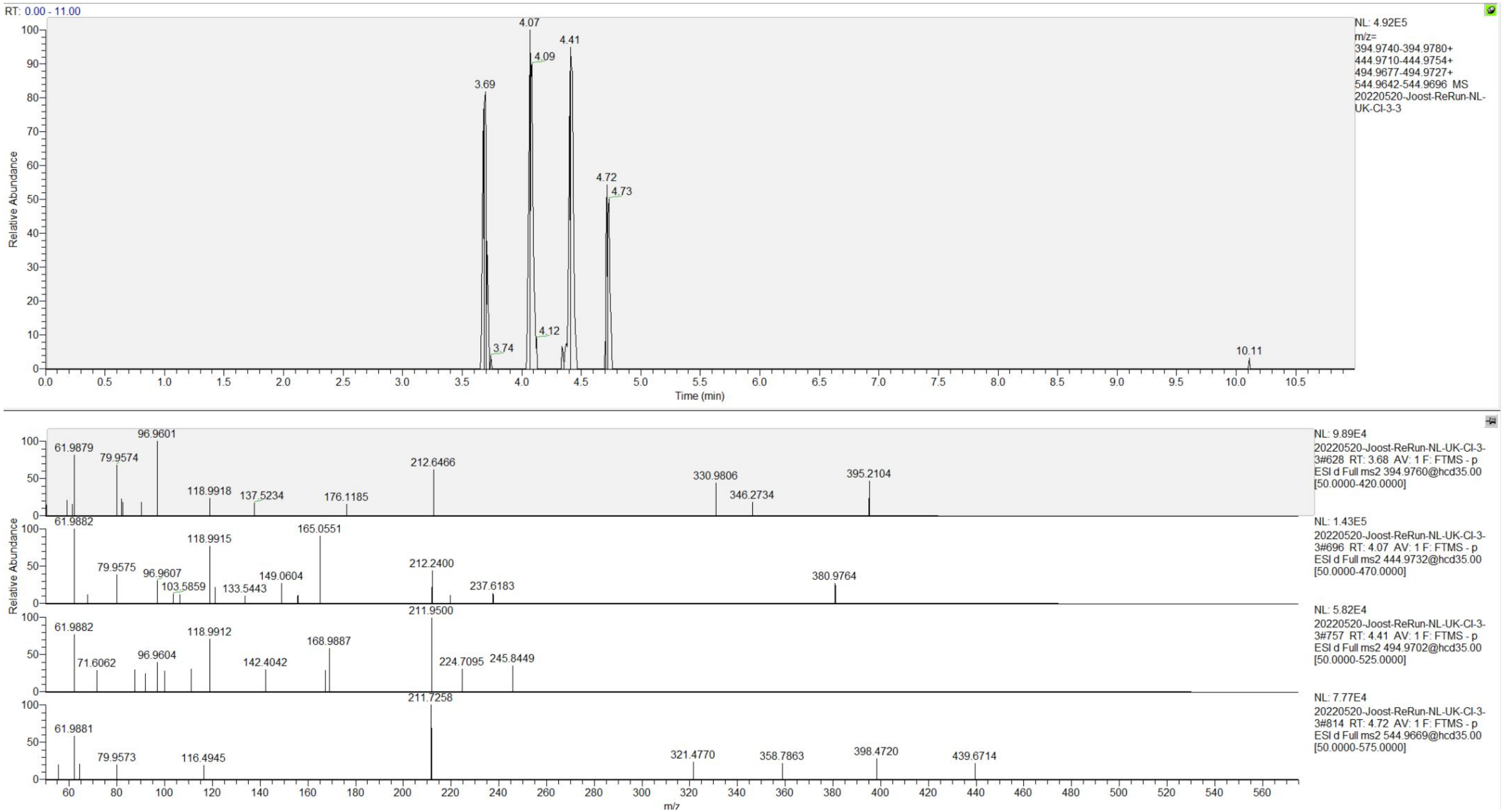

Figure S6. Chromatogram showing extracted masses in full-scan (MS^1) and acquired MS^2 spectra for the C_8-C_{11} H-PFCAs (top to bottom) homologous series in an air sample. Homolog retention times are 3.69 for H-PFOA, 4.07 for H-PFNA, 4.41 for H-PFDA and 4.72 for H-PFUnDA respectively.

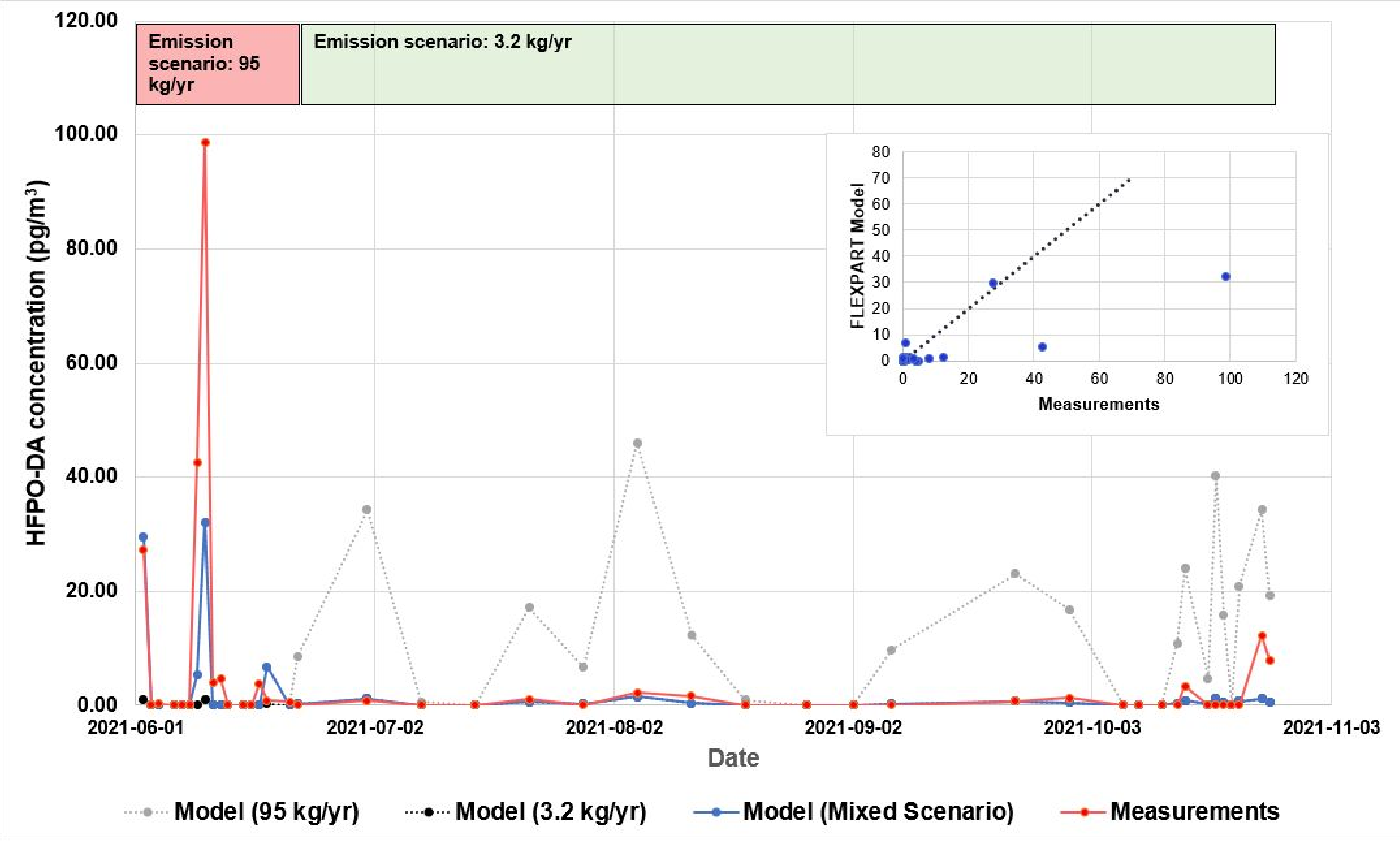

Figure S7. Measured and modelled HFPO-DA concentrations at the Cabauw Meteorological Observatory under the two emission scenarios (95 kg yr^{-1} and 3.2 kg yr^{-1}) as well as the mixed scenario and in the inset graph the measurements vs modelled concentrations under the mixed scenario, showing the 1:1 line.

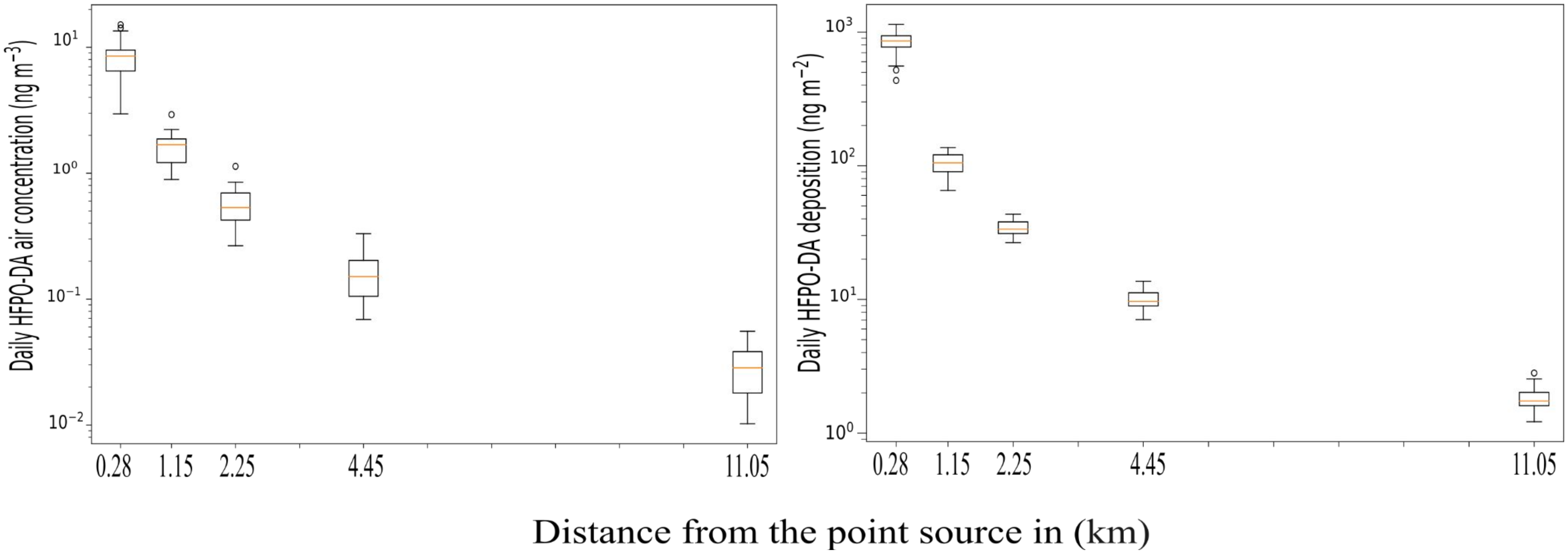

Figure S8. The left panel represents the modeled average daily atmospheric HFPO-DA concentration as a function of distance and the right panel the average daily total HFPO-DA deposition as a function of distance, for June 2021, and using the maximum emission scenario of 95 kg/year. The distances are binned in five bins, with the first bin (0°) representing the grid containing the point source, and distances from source ranging between 0 – 555 m. The second bin (0.01°) represents grid with distances between 555 m – 1.7 km from the source, the third bin (0.02°) represents a grid within the distances between 1.7 km – 2.8 km from the source, the fourth bin (0.04°) represents a grid with distances between 3.9 km – 5 km from the source and the last bin, bin five (0.1°) represents a grid with distances between 10.45 km – 11.6 km from the source. The box and whiskers plots present the median HFPO-DA concentration of the binned distance as the orange line in each box, the upper and lower boundaries of each individual box represent the 75th and 25th percentiles while the whiskers display the interquartile range (IQR) times 1.5 + /- the upper and lower boundary values and the empty circles depict the outliers of each dataset.

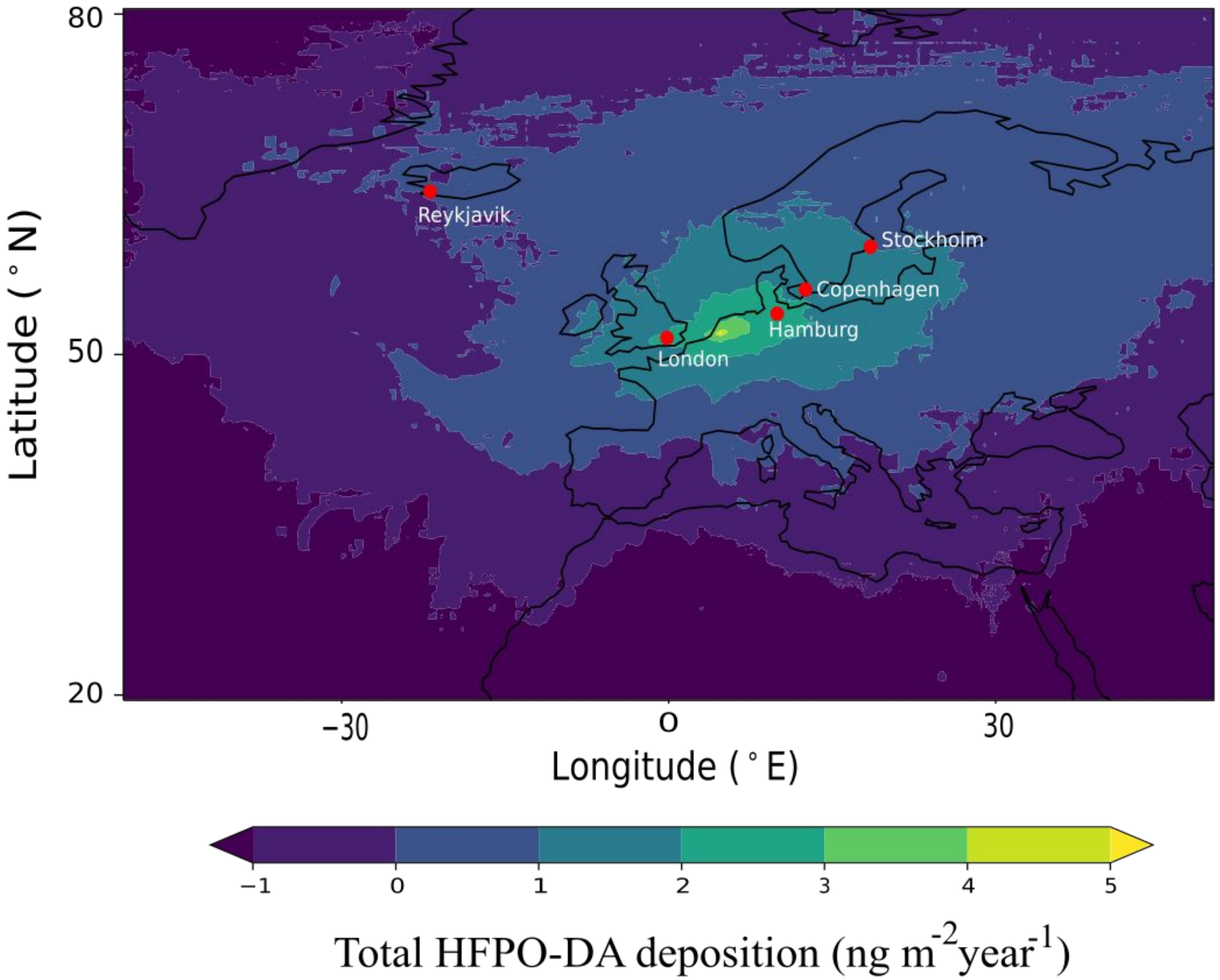

Figure S9. Modeled accumulated wet and dry HFPO-DA deposition after one full year (2020), with the Chemours facility in Dordrecht as the sole point source of the emissions and using the maximum HFPO-DA emission scenario of 450 kg yr^{-2}. The red dots in the plots correspond to different cities in northern Europe mentioned in Table S17 while the scale represents the orders of magnitude with 0 = 1 ng m^{-2} $year^{-1}$.

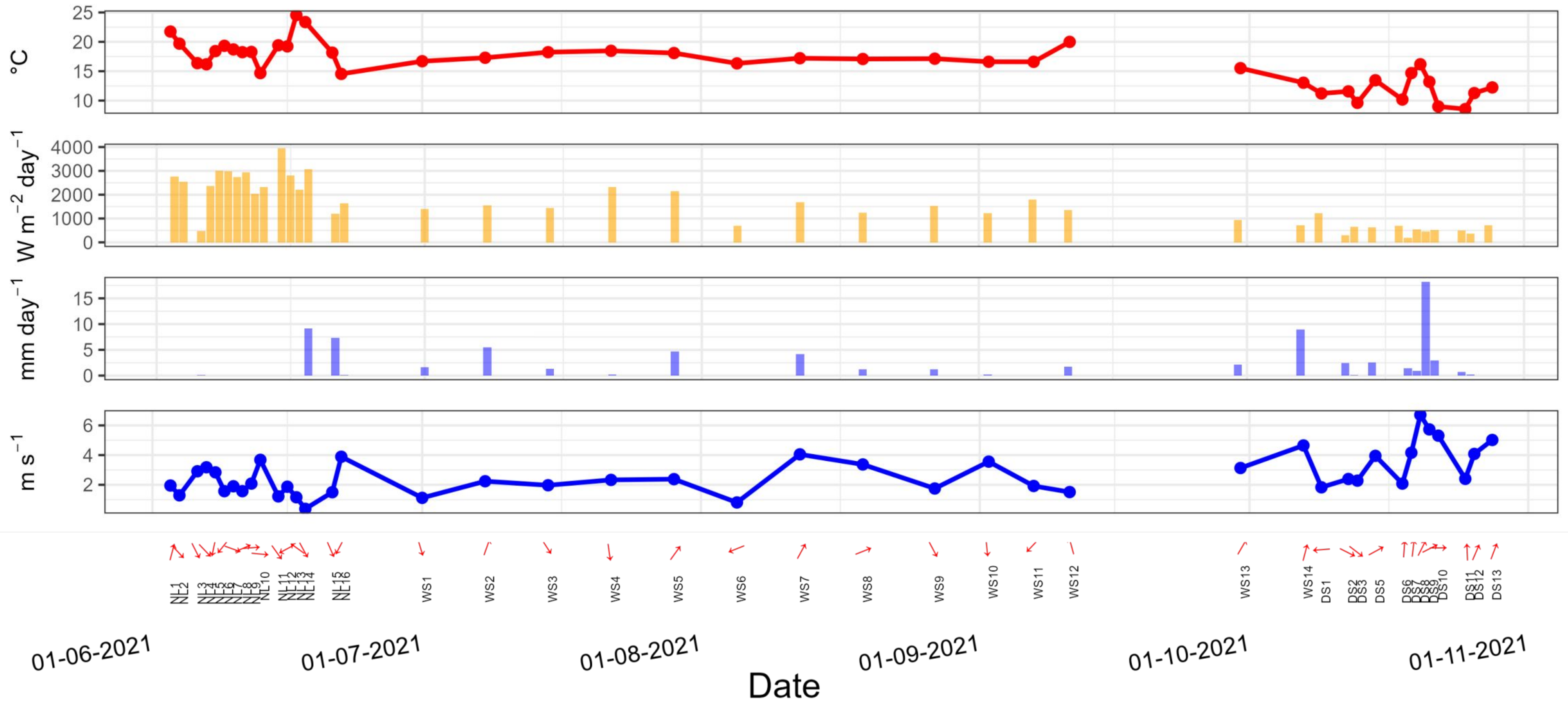

Figure S10. Meteogram showing mean temperature, radiation and precipitation (normalized by day) and wind speed and direction per sample during sampling time at the Cabauw meteorological observatory

References

1. Carslaw DC, Ropkins K. Openair—an R package for air quality data analysis. *Environmental Modelling & Software*. 2012;27: p. 52–61.
