# Supplementary material for: HFPO-DA and Other PFAS in Air Downwind of a Fluoropolymer Production Plant in the Netherlands: Measurements and Modeling
Source: Environ Sci Technol. 2025 Apr 21;59(17):8662–72. doi: 10.1021/acs.est.4c13943 (PMC12060278; doi:10.1021/acs.est.4c13943)
Supplement: Supplementary file 2 — es4c13943_si_002.pdf [file es4c13943_si_002.pdf]

Supporting information 2 for 'HFPO-DA and other PFAS in air downwind of a fluoropolymer production plant in the Netherlands: measurements and modeling' Atmospheric dispersion modeling

Joost Dalmijn [a]*, Julia J. Shafer[a], Jonathan P. Benskin[a], Matthew E. Salter[a], Jana H. Johansson[b] and Ian T. Cousins[a]*

[a]Department of Environmental Science, Stockholm University, SE-10691 Stockholm, Sweden
[b]Department of Thematic Studies—Environmental Change, Linköping University, 581 83 Linköping, Sweden

*E-mail: joost.dalmijn@aces.su.se
*E-mail: ian.cousins@aces.su.se

Summary: this supporting information contains detailed information on model inputs, descriptions of the FLEXPART model, information on model simulations, and additional model results and contains 23 pages, 12 tables and 13 figures.

S2.1 Physical-Chemical Properties

Table S2.1: Physical-chemical properties of HFPO-DA used in the FLEXPART simulation.

HFPO-DA properties	Values
Molar weight (g/mol)	330.5 [2]
Density (kg/m^3)	1850 [2]
Atmospheric half-life	Stable [17;3]

Table S2.1 contains important chemical input parameters of HFPO-DA to the FLEXPART model. Another important property is the acid dissociation constant, pKa, since it helps predict HFPO-DA particle-gas partitioning [1], in the atmosphere. For PFAS the determination of pKa is quite problematic and perfluorooctanoic acid (PFOA) is a prime example. The pKa of PFOA was originally determined using titration techniques that were later shown to overestimate the actual dissociation taking place [10]. This was due to the surfactant nature of PFOA which allows the neutral form to potentially sorb to the water/air interface in turn leading to an overestimated pKa [10]. Another method for determining the pKa of different PFAS is to estimate them using models. All reported pKa values of HFPO-DA and its salt, to date, can be found in Table S2.2. It should be noted that HFPO-DA's estimated pKa of 2.84 and its salt's (GenX) estimated pKa of 3.82 were determined with the same sort of titration method that was shown to overestimate the pKa of PFOA and should thus be considered as quite uncertain.

Table S2.2: Reported pKa values for HFPO-DA an its salt, derived by titration techniques and different models.

Method	Substance	pKa	Reference
Measured (titration)	HFPO-DA	2.84 +/-0.021	DuPont-26349,2008 [16]
Measured (titration)	HFPO-DA	2.45	DuPont-PM-W-726,2011 [8]
Measured (titration)	GenX (salt)	3.82 +/-0.059	DuPont-26349,2008 [16]
Modelled (MarvinSketch v16.10.24)	HFPO-DA	-0.77	ECHA/documents/10162/53
Modelled (OPERA)	GenX (salt)	-0.77	Hopkins et al, 2018 [12]
Modelled (Chemicalize, ChemAxon)	HFPO-DA	-0.77	Hopkins et al, 2018 [12]
Modelled(SPARC)	HFPO-DA	0.06	Gomis et al, 2015 [9]

$$(1-\alpha) = \frac{1}{(1+10^{\,\mathrm{pH}-\mathrm{pKa}})} \tag{1}$$

Equation 1, (The Henderson–Hasselbalch equation) was used to calculate the speciation of an acid, where $(1-\alpha)$ describes the fraction of the anionic phase occurring based on the pKa of the acid and the current environmental pH.

According to Karydis et al. [14] the pH of aerosols in the atmosphere over Europe during 2011-2020 was between 2.6 - 6.7 with an average of pH of 3.9. To get a sense of the HFPO-DA speciation based on its different pKa values and the variability within the atmospheric acidity, the anionic fraction was calculated using equation (1). The calculated percentage of the anionic form in each scenario is presented in Table S2.3 and clearly shows that HFPO-DA is predominantly an anion in the atmosphere apart from when the lowest pH and the largest pKa values are assumed. It should again be noted that the pKa of 2.84 was deduced from a titration method which has been shown to be skewed when analyzing surfactants [10]. It should also be noted that PFAS in its anionic form

results in low volatility and high solubility[1] which in turn favors HFPO-DA in the particle phase in the atmosphere.

Table S2.3: Anionic speciation of HFPO-DA and its dependence on the different reported pka values along with the different potential pH values of aerosols in Europe.

pKa	pH	Speciation
-0.77	6.7	99.99
-0.77	3.9	99.99
-0.77	2.6	99.96
1.81	6.7	99.99
1.81	3.9	99.19
1.81	2.6	86.01
2.84	6.7	99.98
2.84	3.9	92.00
2.84	2.6	36.53

S2.2 HFPO-DA Emissions

Table S2.4: Permitted and consequently estimated HFPO-DA emissions to air between 2012, when emissions started in Dordrecht, up until 2021 and are based on Dutch authority permits. Numbers in brackets note changes in the permitted emissions during the permitting year.[6;7]

Year	Maximum HFPO-DA permitted to air ($kg\,yr^{-1}$)
2012	660
2013	660
2014	660
2015	660
2016	660
2017	450
2018	450
2019	450
2020	450 (95)
2021	95 (3.2)

S2.3 Sensitivity analyses

S2.3.1 Particle mass and size distribution determination

Since particle size is an important determining factor for the atmospheric dispersion of aerosols, HFPO-DA's particle mass distribution had to be estimated[11;5]. The HFPO-DA particle mass and size distribution were determined by utilizing a paper detailing the measured particle concentration of different PFAS in the atmosphere of different Asian cities[15]. Tsukuba, Japan had a strong HFPO-DA signal and was thus used as a proxy. It should be noted that the nearest known point source from a Chemours facility in Shimizu, Shizuoka Prefecture, Japan was about 180 km away from the measurement site and the size distribution might thus be favoring the smaller size-ranges. Five samples, presented as the concentration (pg/m^3) over four size bins, (>10 μm, 2.5 - 10 μm, 1 - 2.5 μm, < 1 μm), were detailed in the study and these were consequently converted into particle mass size distribution (dM/dlogDp) for each bin following Equation 2 and the geometric mean was calculated from the ranges within each size bin following Equation 3. The median of the five

samples, for each particle mass and size distribution, were plotted over the geometric mean which is represented as blue dots in Figure S2.1. Two lognormal modes, were fitted to the distribution and the median diameters, standard deviation and mass fractions for both modes could thus be determined.

$$(\mathrm{dM/dlogDp}) = (\mathrm{Conc})/\mathrm{Log}(\mathrm{Upper}) - \mathrm{Log}(\mathrm{Lower}) \tag{2}$$

Equation 2 depicts the conversion from size-resolved particle concentration to the particle mass and size distribution. Where "Upper" represents the upper end of the size bin and "Lower" the lower end of the size bin. E.g. If a size bin ranges between 0.1 µm - 1µm, Upper would be 1 µm and Lower 0.1 µm[13].

$$\text{Geometric mean diameter} = \sqrt{(\mathrm{Upper} + \mathrm{Lower})} \tag{3}$$

Equation 3 depicts the calculation for the geometric mean diameter for each size bin[13].

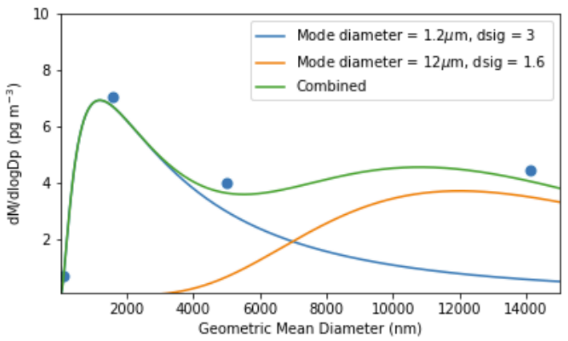

Figure S2.1: This figure depicts two lognormal modes fitted to represent the overall particle size distribution. The blue line, mode 1, represents 74% of the total mass with a mean diameter of 1.2 µm and a sigma of 3. The orange line, mode 2, represents 26% of the total particle mass with a mean diameter of 12 µm and a sigma of 1.6. The green line, the combined mode, represents 100 of the total mass and the blue dots represent the actual particle distribution based on data from Lin et al, 2020[15].

Even though mode 1 seems to represent a larger number of particles, the larger particles, in mode 2, weigh more (Figure S2.1). By calculating the area under the two curves, mode 1 and mode 2, the total mass could be assessed. To get the mass percentage of each size bin, both mode 1 and mode 2 were divided by the total mass. The mass percentage for each mode was thus established to be approximately 19% for mode 1 and 81% for mode 2.

S2.3.2 Spatial resolution

The accuracy of an aerosol's atmospheric trajectory and consequent deposition is highly dependent on spatial and temporal resolution[20]. Since the meteorological data has a set temporal resolution of 3 h, the spatial resolution was the only variable parameter. In order to test the impact of the model's spatial resolution on the particle's atmospheric dispersion and to determine an appropriate spatial resolution to use, two sensitivity analyses were conducted. The first analysis was conducted to establish the resolution settings needed to run FLEXPART in a large-scale simulation with a

domain with coordinates [lon -60 - 71, lat 0 – 86] and the second analysis was conducted to establish the resolution most appropriate for a local simulation with a domain of 50 km x 50 km. The large-scale resolution was determined by running seven simulations with resolutions set between 0.1 and 3 degrees and the local simulation by running 6 simulations between 0.001 – 0.006 degrees. Figure S2.2 and Figure S2.3, show how the atmospheric trajectories are impacted by the change in spatial resolution and represent the large-scale and local scale respectively.

For the large-scale analysis (Figure S2.2), A represents the smallest grid size of 0.1 degrees and shows the most specific trajectory whereas G, representing the largest grid size of 3 degrees, shows the most diffused and spread trajectory and is less reliable. Thus, A was chosen as the resolution parameter moving forward.

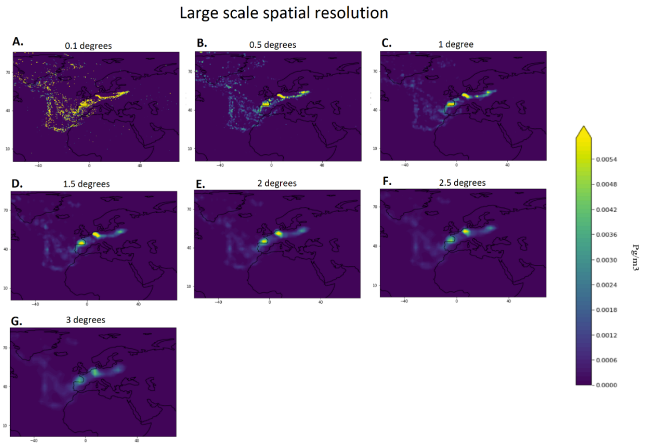

Figure S2.2: Particle concentration in air and aerosols tracers' trajectories for 7 simulations with different spatial resolutions. The simulations ranged between the highest resolution, A to the lowest resolution, G.

For the local simulation (Figure S2.3) the same holds true, where A is more specific and F is more diffuse. However, since running a 0.001 resolution is extremely computationally exhausting and would result in a very slow run, B (0.002 degrees) was chosen instead. This resolution also showed a good spatial accuracy and was slightly less demanding computationally.

S2.3.3 Sampling rate

The FLEXPART concentration output is representative of a time-averaged concentration over a given time period. The time-averaged concentration is based on the number of hypothetical samples collected within a specified interval which is set in seconds in the COMMAND file under LOUTSAMPLE and LSYNCHTIME. The shorter the LOUTSAMPLE and LSYNCHTIME, the more samples are collected and subsequently a higher accuracy can be achieved[19]. However, the computational time will also increase with the decrease of interval length. Thus, to assess what sampling rate was most appropriate for the large-scale and the local simulation, two sensitivity analyses were conducted.

For the large-scale simulation, the model was run for 30 days, with LSYNCHTIME and LOUTSAMPLES set to interval lengths between 100 and 900 s (the average concentration time, set to

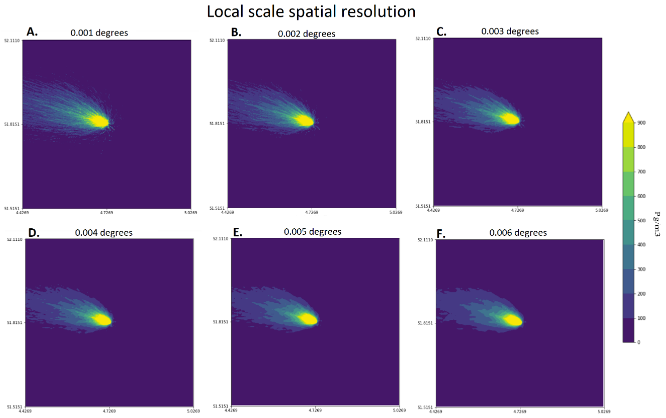

Figure S2.3: Particle concentration in air and aerosol tracers' trajectories for the 6 local simulations with different spatial resolutions. The simulations ranged between the highest resolution, A to the lowest resolution, F.

3600 s in the COMMAND file, has to be a multiple of the LSYNCHTIME and thus 500, 700 and 800 s intervals were excluded) to determine what setting was most appropriate considering both output results and runtime. In Figure S2.4, the total atmospheric concentration at 6 different time steps for each interval is depicted. It is quite clear that the simulations using intervals of 100, 200 and 300 s gave very similar results whereas intervals 400, 600 and 900 s intervals, linked to a lower accuracy, showed similar concentrations. Since the computation time and accuracy are increased with a decreased interval and since intervals 100, 200 and 300 produced similar results, an interval of 300 s should suffice moving forward.

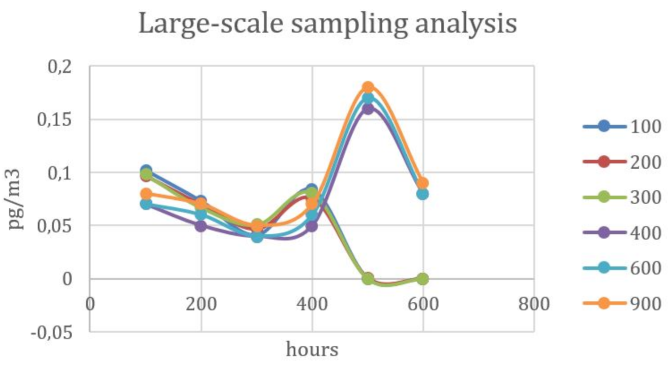

Figure S2.4: Average atmospheric concentration using 6 different intervals at 6 different time points. The shorter intervals (100 s, 200 s and 300 s) which are said to be more accurate are very similar whereas the longer intervals (400 s, 600 s and 900 s) deviate from the smaller intervals.

For the local simulation, the model was run for 10 days (since the local simulation is more computationally intense than the large-scale simulation) with LSYNCHTIME and LOUTSAMPLES set to interval lengths between 10 and 90 s (the average concentration time, set to 3600 s in the COMMAND file, has to again be a multiple of the LSYNCHTIME and thus the 70 s interval was excluded). In Figure S2.5, the average atmospheric concentration at the plume at 6 different time steps for each interval is depicted. Here it can be seen that the concentration decreases with increased sampling and since the accuracy does as well[19], the 10 s interval is clearly the best option. However, since the run using 10 s intervals took almost twice as long as using the 20 s interval, the 20 s interval was chosen instead, to save time and since results still appeared similar.

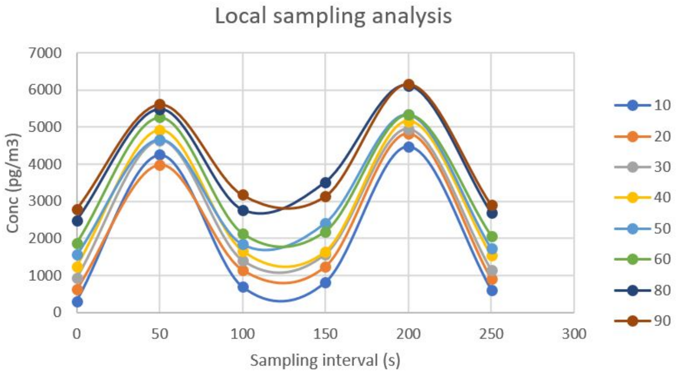

Figure S2.5: Concentration dependence on the sampling settings, ranging between 10 s to 90 s at the point source and for 6 different times

S2.3.4 Theory - Particle deposition

The two types of loss processes relevant for simulating the air dispersion ability in the particle phase, using FLEXPART, are dry particle removal and wet particle removal and both depend on

meteorological data and the specific particle's properties[11].

Dry removal is primarily dependent on dry particle deposition which is in turn, determined by the particles settling velocity but also by the resistance between air and the surface layer and the viscous sublayer between air and surface[4]. Dry particle deposition is therefore very dependent on particle size and density but also on environmental parameters regarding surface structures as seen in Equations 4 and 5[4].

$$v_g = \frac{gp_p d_p^2 C_{cun}}{18_\mu} \tag{4}$$

Equation 4 describes the settling velocity ($v_g,\ m/s$) of a specific particle as a function of the gravitational acceleration ($g,\ m/s^2$) particle density ($p_p,\ kg/m^3$) , its particle diameter ($d_p,\ m$), the dynamic air viscosity ($\mu,\ N/m^2$) and also the Cunningham correction constant ($C_{cun},\ dimensionless$).[4]

$$v_d = (r_a(Z) + r_b + r_a(Z) r_b v_g)^{-1} + v_g \tag{5}$$

Equation 5 describes the dry particle deposition velocity ($v_d,\ m/s$) as a function of the resistance between surface and height (Z) in air ($r_a(Z), s/m$), the resistance of the viscous layers between air and surface ($r_b,\ s/m$) and a term describing impaction ($r_a(Z) r_b v_g$) and the settling velocity of the particle ($v_g,\ m/s$)[4].

The particle diameter (d_p) and density (p_p) are the two inputs, set by the model user, that will highly affect the deposition rate whereas the other parameters are determined by meteorological data and FLEXPART files describing the surface layer and environmental conditions[4].

For wet particle removal two processes take place, in-cloud and below-cloud scavenging. The below-cloud scavenging is dependent on the collision probability of rain/snow with the particles but also on the attachment probability of the particle onto a rain droplet or a flake, thus the size of both particle and hydrometer, the intensity of the precipitation and the chemistry of the particle will all affect the below-cloud scavenging efficiency[11;22]. The equation determining below-cloud scavenging (Λ), seen in Equation 6, is incorporated in FLEXPART simulations.

$$Log_{10}\frac{\Lambda}{\Lambda_0} = C_*(a + bD_p^{-4} + cD_p^{-3} + dD_p^{-2} + eD_p^{-1} + f(\frac{I_s}{I_0})) \tag{6}$$

Equation 6 represents the below-cloud scavenging calculation used in FLEXPART to determine what fraction of the suspended particles are removed by this process. $\Lambda,\ s^{-1}$ represents the below-cloud scavenging coefficient which is dependent on the particle diameter ($dp,\ m$), $dp = 10^{Dp} * dp_o$, the precipitation rate ($I_{s/0},\ mm/h$), specific properties of rain droplets or snowflake (a-f) and the term C^* , which represents the specific properties of the actual particle in regards to either rain droplet or snowflake removal[18;11].

The particle mean diameter (d_p) and collection efficiency (C^*) are thus the two inputs that will affect the below- cloud scavenging parameter based on the property of a particle, whereas the other variables, like rain droplet and snowflake properties (a-f) and precipitation rate ($I_{s/0}$), are determined by the actual meteorological data for the specific period of the simulation.

Below-cloud particle scavenging, along with dry particle removal, is thought to be least effective for particles in the 0.1-0.2 µm range and to increase in importance with the increase in particle size. Below-cloud scavenging is also most relevant between ground level and 1000 m above ground, but will depend on the meteorological data and location of the actual clouds[11].

In-cloud scavenging is thought to be the most important removal process for particles in the submicron size range and since the scavenging takes place inside of clouds this removal process is most relevant above 1000 m, in the free troposphere[11]. Particles released close to ground level will

initially be affected by dry and below-cloud processes until they reach higher altitude [11]. In-cloud particle scavenging, equations 7 and 8, are used in FLEXPART to describe the particle removal from clouds based on a particle's nucleation efficiency and is also dependent on relevant weather conditions.

$$\Lambda = F_{nuc} * \frac{I}{PCW} * ic_r \tag{7}$$

Equation 7 represents the scavenging coefficient (Λ, s^{-1}) in terms of in-cloud particle deposition and is dependent on the nucleation efficiency (F_{nuc}, *dimensionless*) of the particle, the washout ratio of cloud water (I/PCW), determined by the precipitation intensity (I, mm/h) and the sub-grid precipitating cloud water (PCW, mm/h) and the water replenishment factor (ic_r, s^{-1}). I/PWC and ic_r are input parameters based on the meteorological and FLEXPART files repressively, whereas F_{nuc} is particle dependent and will vary based on the specific particle's property assumptions [18;11].

$$(F_{nuc}) = (1 - \alpha) * CCN_{eff} + \alpha * IN_{eff} \tag{8}$$

Equation 8 describes F_{nuc} which in turn describes a specific particle's efficiency of forming snowflakes or raindrops and the Cloud Condensation Nucleation efficiency (CCN_{eff}) and the Ice Nucleation efficiency (IN_{eff}) parameters are both particle dependent. $(1 - \alpha)$ is used to assess the nucleation efficiency when the precipitation is made up of a mix of rain and snow [18;11].

The CCN_{eff} and IN_{eff} parameters are dependent on both the size and chemical properties of the particle and is thus set by the model user, soluble particles are believed to be effective scavengers for CCN_{eff} whereas IN_{eff} favors solid aerosols over liquid ones according to Grythe et al. [11].

S2.3.5 Deposition parameters

As described in section S2.3.4, particle properties that are said to determine dry and wet deposition are density, particle diameter, C*, CCN_{eff}/IN_{eff} and thus five HFPO-DA specific scenarios were tested as seen in Table S2.5, The scenarios tested were: a minimum scenario, a maximum scenario, two mixed scenarios and a default scenario specifically recommended by Grythe et al, (2017) [11] when dealing with soluble particles. All five scenarios were run based on five potential particle size bins and density was not tested since it is a static chemical physical property and was hence set to 1850 kg/m^3 in each scenario.

Table S2.5: Five scenarios, using the minimum wet particle deposition parameters and the maximum wet particle deposition parameters, two mixed scenarios and the default settings based on a soluble particle for 6 relevant particle size buckets for all the ten thousand particles released.

Scenario	**In cloud**		**Below cloud**		**Concentration** (ng/m^2)					
	C_{rain}	C_{snow}	CCN_{eff}	IN_{eff}	0.05μm	0.1μm	0.5μm	1μm	5 μm	10 μm
min	0.1	0.1	0.1	0.1	2200	1638	1604	2475	3973	4087
max	10	10	1	1	3775	3645	3756	3907	4079	4119
default	1	1	0.9	0.1	3347	3123	3164	3514	4046	4106
mix 1	1	1	0.1	0.1	787	1953	2058	2903	4022	4100
mix 2	0.1	0.1	1	1	3307	3097	3088	3424	4020	4093

To illustrate the difference between the most efficient scavenging scenario and the least efficient scenario, two maps were plotted illustrating wet and dry deposition on the last day of the 30-day simulation for the mean particle size of 0.5 µm, which can be seen in Figure S2.6. It becomes

clear that the amount of HFPO-DA removed in a month is highly dependent on the scavenging coefficients.

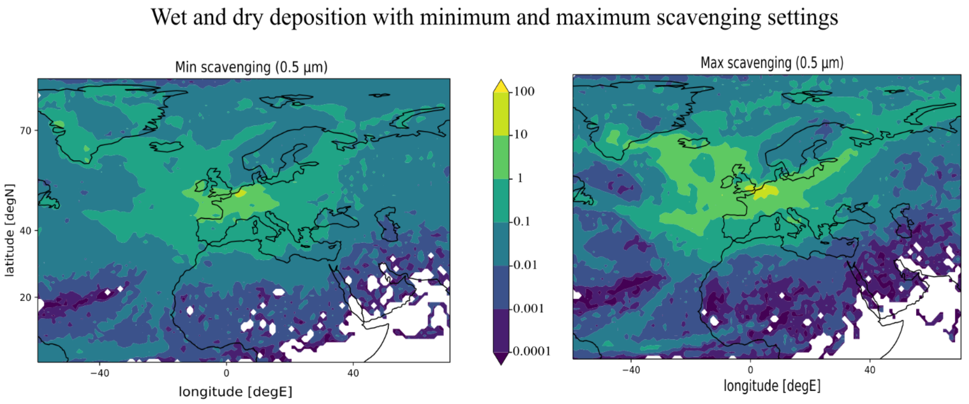

Figure S2.6: Maximum and minimum scavenging scenarios for a particle size of 0.5 µm after a month (700 h) of HFPO-DA release from Dordrecht, Netherlands and its impact on overall particle deposition.

To illustrate how the deposition rates, vary based on the scavenging coefficients but also on size, results retrieved from the sensitivity analysis were plotted. All plots represent the last day of a 30-day simulation and use the 6 size bins. Plot A in Figure S2.7, depicts the most efficient, the least efficient and the default deposition scenarios for wet and dry deposition. This plot highlights the importance of choosing appropriate scavenging coefficients for submicron particles and illustrates the unimportance of coefficients for larger particles. Plot B depicts the dry deposition process and shows how larger scavenging coefficients result in less dry deposition and lower coefficients in higher deposition. Plot C depicts wet deposition only and clearly illustrates the high dependency the scavenging coefficients have on wet deposition, whereas plot D depicts two mixed scenarios for wet deposition, which illustrates how in-cloud scavenging is the most efficient process for particle removal of submicron sizes and how below-cloud scavenging dominates for larger particles. All results align well with earlier work[11;22] and suggest that the default scavenging parameters suggested by Grythe et al (2017)[11], will represent an intermediate scenario between the two extremes, beneficial for a HFPO-DA which is believed to favor the submicron size range[15].

To visualize the air dispersion of the default scavenging setting for the different size bins, the six scenarios were plotted on a map, seen in Figure S2.8. This figure highlights the impact that particle size has on a particle's air dispersion ability, making it clear that particles in the submicron bins including 1 µm, are able to transport the furthest in the atmosphere and HFPO-DA particles of this size range run the highest risk of undergoing LRAT.

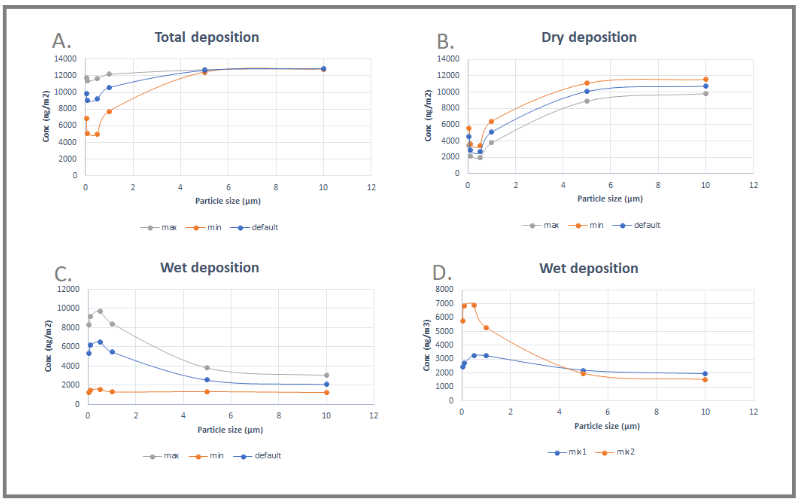

Figure S2.7: Four results from the particle removal sensitivity analysis. A) depicts the maximum scenario with $C^* = 10$ and CCN/IN_{eff} =1, a minimum scenario C^*=0.1 and CCN/IN_{eff} =0.1 and the default scenario of $C^* = 1$ and CCN/IN_{eff} =0.9, B) depicts dry deposition processes over the range of sizes and for scavenging parameters min, max and default, C) depicts wet deposition only for 3 scavenging scenarios; minimum, maximum and default and D) depicts two mixed scenarios. Mix1 with $C^* = 1$ and CCN/IN_{eff} =0.1 and mix2 with CCN/IN_{eff} =1 and $C^* = 0.1$.

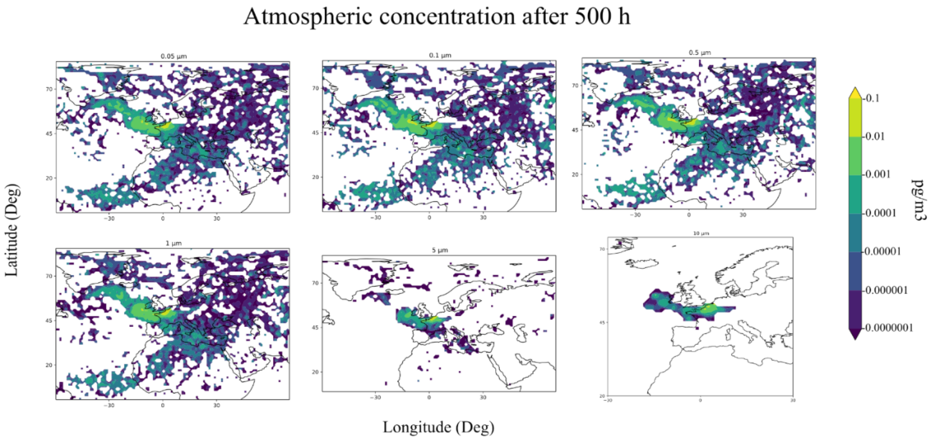

Figure S2.8: Results from model simulations using the intermediate efficient scavenging parameters for all the different size buckets at hour 500. It can be seen quite clearly that HFPO-DA's air dispersion ability is directly correlated to particle size. The sizes range between 0.5 and 1 µm lead to the highest atmospheric concentration and the largest spatial distributions

S2.3.6 Particle amount

The number of particles that can be released are set in the the RELEASE file and range from ten to many millions of particles and the time of a simulation is directly correlated to the number of particles released[18]. To ensure that enough particles are employed to accurately answer the questions at hand while concurrently minimizing the time intensity of the simulation, two sensitivity analyses were performed.

For the large-scale simulation, 6 model-runs were completed with the number of particles released ranging betwween ten thousand to fifteen million and for the local simulation, particles released varied between ten thousand to two million.

By analyzing Figure S2.9, it becomes quite clear that a release of between ten million and fifteen million particles results in very similar density and hence the analysis concludes that ten million particles should be sufficient for the large simulation since fifteen million particles presented a huge computational cost with little gain. By analyzing Figure S2.10, representing the local analysis, two million particles are shown to produce the best results.

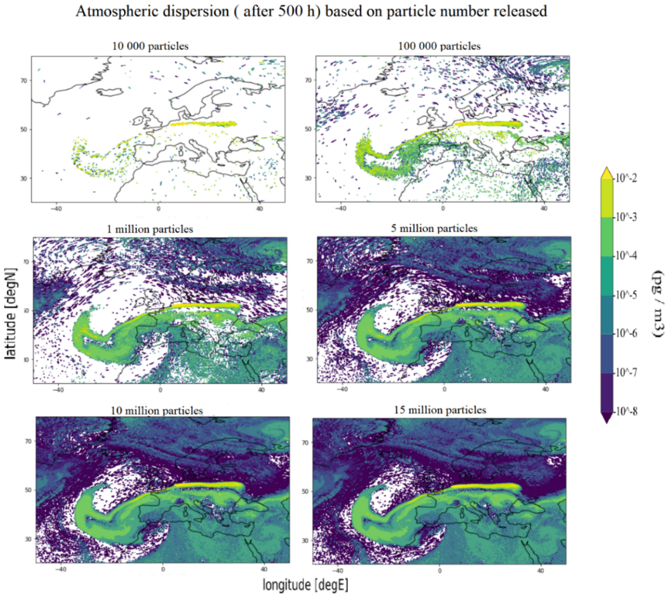

Figure S2.9: Multiple simulations using different numbers of released particles, ranging from ten thousand to 15 million. The particle density is represented by the atmospheric concentration at 500 hours into the simulation for the total area of interest (Lon -50 - 50, Lat 0 - 90) for the different releases. What can be seen is that the accumulated particles concentration tapers off with little to no difference between ten million and fifteen million particles.

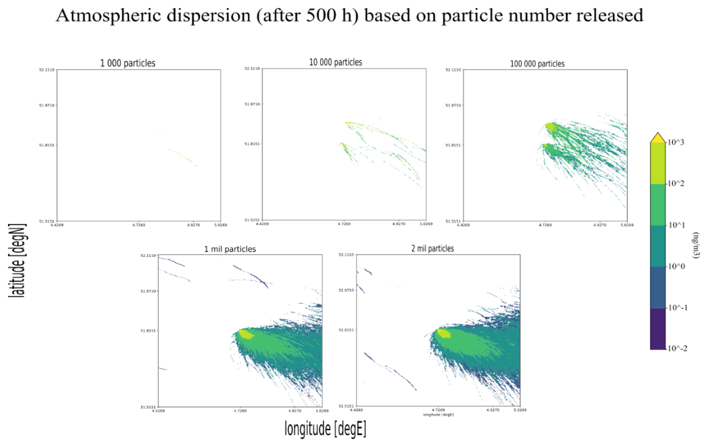

Figure S2.10: The effect of released particle amounts on the local simulation, showing that a simulation using two million particles is the best option.

S2.4 FLEXPART

S2.4.1 Installation

FLEXPART version 10.4 was downloaded from https://flexpart.eu/wiki/FpDownloads with the help of Matthias Brakebusch and installed/set up by following instructions from Pisso et al (2019). The meteorological data used for this project was retrieved from the ECMWF's database with support from Sabine Eckhardt and converted into appropriate input files for FLEXPART, with the open-source software bundle, Flex Extract[21]. A laptop equipped with a VPN was used to allow access to the server situated at Stockholm University. MobaXterm and a SSH key was further used to access a Linux terminal and the virtual machine where FLEXPART was stored and run. Once FLEXPART had completed a simulation, the output files were produced as NetCDF files that were further interpreted with Jupyter Notebook and Python code into tangible results.

S2.4.2 Bug in the code

When analyzing FLEXPART deposition outputs, negative deposition values were discovered. They were very small negative values, but should not have been there since a deposition can never be negative. After running multiple simulations, modifying settings with no change and consulting with Sabine Eckhardt, Ignacio Pisso and Petra Essei from FLEXPART.eu's ticket service, the issue was determined to be due to a bug in the FLEXPART code, specifically during the process where FLEXPART converts its outputs into the NetCDF format. The default FLEXPART output, a sparse matrix binary format, automatically filters spurious negative values and thus negative values did not occur in the default output. To decide how to move forward, two output files were compared, where the only difference between them was output format. This analysis showed that setting negative values to zero in the NetCDF format produced an identical version of the binary format and this was thus the route used to proceed. According to Igancio Pisso, the bug will be patched in the next FLEXPART release and should thus not be an issue in the future.

S2.4.3 Key user file settings

Below, five tables are listed that describe the settings of the five, key user FLEXPART files, COMMAND, OUTGRID, RELEASE and SPECIES, which determine the simulation of HFPO-DA.

The COMMAND file is used to set the simulation protocol and all settings in bold were default settings from Pisso et al (2019) that did not need to be changed. Table S2.6 represents the settings for the large-scale simulation and Table S2.7, represents the settings required when running the local simulation. The start and end dates were adjusted to fit the specifics of this study, IOUT was adjusted to produce the outputs as NetCDF files, IPOUT was adjusted to produce particle position files and LCONVECTION was turned on to allow for convective parameterization to be represented in the simulation. For the large-scale simulation, the CTL of -5 and IFINE of 4 was kept as presented in Pisso et al, 2019. If CTL is below zero it is computationally less intense and turbulence in the simulation is less detailed. However, according to Stolh et al (2005) this is a sufficient mode for large-scale simulations. For the local run, the CTL and IFINE were changed to 20 and 2 respectively, based on suggestions from Massimo Cassiani, to better describe the turbulence taking place. The LOUTSAMPLER and LSYNCTIME were set in table S6 based on sensitivity analysis B3 and changed in the local simulation to 20 s for each based on Massimo Cassini's suggestions.

Table S2.6: This COMMAND file describes the parameters used in the large-scale simulation. The bold text represents input that were left as described in Pisso et al, 2019 [18] and others were changed specifically for the HFPO-DA simulation based on sensitivity tests. A zeros mean that a specific setting is turned off.

COMMAND FILE	SETTING	EXPLANATION
LDIRECT	1	Forward mode
IBDATE	20200101	Start date
IBTIME	**0600**	Start time
IEDATE	20201231	End date
IETIME	**180000**	End time
LOUTSTEP	**3600**	Average output (s)
LOUTAVER	**3600**	Output interval (s)
LOUTSAMPLER	300	Sampling interval (s)
ITSPLIT	**999999**	Particle splitting int. (s)
LSYNCTIME	300	Synchronized time interval
CTL	**-5**	LSYNCTIME (Random walk)
IFINE	**4**	Vertical transport (s)
IOUT	9	Output mass and netcdf format
IPOUT	2	Particle position output at end
LSUBGRID	**0**	Orographic variation
LCONVECTION	1	Convection parameterixation
LAGESPECTRA	**1**	Age spectra calculation
IPIN	**0**	Warm start
IOUTPUTFOREACHRELEASE	1	Output for release location
IFLUX	**0**	Mass fluxes output through grid box boundaries
MDOMAINFILL	**0**	Domain filling
IND-SOURCE	**1**	Source unit mass
IND-RECEPTOR	**1**	Receptor unit mass
MQUASILAG	**0**	Track individual particles
NESTED-OUTPUT	**0**	Nested output
LINIT-COND	**0**	Bwk mode only
SURF-ONLY	**0**	Output for lowest model layer only
CBFLAG	**0**	Skewed not Gaussian

The OUTGRID file is used to set the modeled domain and the spatial resolution of interest. The spatial resolution (DXOUT/DYOUT) was adjusted, for the large-scale simulation based on the results from the spatial resolution sensitivity analysis (B2) and the spatial resolution for the local simulation was changed based on suggestions from Massimo Cassini. OUTHEIGHTS were kept as the default settings from Pisso et al (2019) [18] for the large-scale simulation to represent all heights

Table S2.7: This COMMAND file was used in the local simulation. The bold text represent inputs that were left as described in Pisso et al, 2019 and the regular text depicts inputs specifically changed for the HFPO-DA species. Sample and synch times were reduced from 300 to 20 (from large scale to local scale) based on a sensitivity analysis described in B3 and the CTL and IFINE were changed from -5, 4 for the large scale, based on Pisso et al (2019)[18] to 20, 2 based on Massimo Cassiani's recommendation.

COMMAND FILE	SETTING	EXPLANATION
LDIRECT	1	Forward mode
IBDATE	20200101	Start date
IBTIME	**0600**	Start time
IEDATE	20201231	End date
IETIME	**180000**	End time
LOUTSTEP	**3600**	Average output (s)
LOUTAVER	**3600**	Output interval (s)
LOUTSAMPLER	300	Sampling interval (s)
ITSPLIT	**999999**	Particle splitting int. (s)
LSYNCTIME	300	Synchronized time interval
CTL	**-5**	LSYNCTIME (Random walk)
IFINE	**4**	Vertical transport (s)
IOUT	9	Output mass and netcdf format
IPOUT	2	Particle position output at end
LSUBGRID	**0**	Orographic variation
LCONVECTION	1	Convection parameterixation
LAGESPECTRA	**1**	Age spectra calculation
IPIN	**0**	Warm start
IOUTPUTFOREACHRELEASE	1	Output for release location
IFLUX	**0**	Mass fluxes output through grid box boundaries
MDOMAINFILL	**0**	Domain filling
IND-SOURCE	**1**	Source unit mass
IND-RECEPTOR	**1**	Receptor unit mass
MQUASILAG	**0**	Track individual particles
NESTED-OUTPUT	**0**	Nested output
LINIT-COND	**0**	Bwk mode only
SURF-ONLY	**0**	Output for lowest model layer only
CBFLAG	**0**	Skewed not Gaussian

within the lower and upper atmosphere but were changed in the local simulation to focus on the lower atmospheric levels to better represent conditions from HFPO-DA's air sampling campaign (at ground level). The other settings, longitude, latitude and grid points were set to represent the desired modelled area of interest. The final settings of the OUTGRID file for both large-scale and local simulations can be seen below, in Table S2.8, S2.9.

Table S2.8: FLEXPART outputs and longitude and latitude settings, defining the left bottom corner for the large-scale simulation. The NUMGRID X and Y describe the number of grids in latitudinal and longitudinal direction respectively and DOUT X and Y describes the size of each grid and will therefore determine the overall size and spatial resolution of output[18]. The OUTHEIGHT determines the different atmospheric heights taken into an account.

OUTGRID FILE	Large-scale	EXPLANATION
OUTLONG	-60	Longitude of lower left corner
OUTLAT	0	Lattitude of lower left corner
NUMXGRID	1310	Grid Points X direction
NUMYGRID	860	Grid points X direction
DXOUT	0.1	Grid distance X direction (spatial resolution)
DYOUT	0.1	Grid distance Y direction (spatial resolution)
OUTHEIGHTS	**100, 500, 1000, 50000**	Sample heights (m)

Table S2.9: FLEXPART OUTGRID settings for the local simulation. By setting the longitude and latitude, the left bottom corner is defined and NUMGRID X and Y describe the number of grids in latitudinal and longitudinal direction respectively. The DOUT X and Y describes the size of each grid and will therefore determine the overall size and spatial resolution of the output[18] and was changed from 0.1 to 0.002 based on sensitivity analysis in section S4. The OUTHEIGHT determines the different atmospheric heights represented in the output and was changed to represent conditions during atmospheric samplings.

OUTGRID FILE	Local	EXPLANATION
OUTLONG	4.42695	Longitude lower left corner
OUTLAT	51.51512	Latitude lower left corner
NUMXGRID	300	Grid X direction
NUMYGRID	300	Grid Y direction
DXOUT	0.002	Grid dist. X direction
DYOUT	0.002	Grid dist. Y direction
OUTHEIGHTS	**10, 20, 30, 40, 50, 60, 70, 80, 90, 100, 200, 300, 400**	Sample heights (m)

The RELEASE file is used to determine the specifics surrounding the species release and the location of the release. The specific settings used in this study and the explanation of these settings can be seen in Table S2.10. Since the settling velocity differed between the two HFPO-DA modes, calculated in B1, two separate releases were set. The NSPEC and MASS settings were adjusted to align with the Particle mass and size distribution determination section B1 and the PART setting was adjusted in both the large-scale and local simulation to align with the results from Particle amount Sensitivity analysis in section 7. The only difference between the RELEASE files for the large-scale and local simulation were the particle release and date, and both settings are depicted with a slash in Table S2.10.

The SPECIES files are used to set information regarding the actual substances being released and the specific settings for this study can be seen in Table S2.11. HFPO-DA was released as two species at two release points due to the two modes' different settling velocities[11]. The PCRAIN-AERO/PCSNOE-AERO and the PCCN-AERO/PIN-AERO parameters were adjusted to align with

Table S2.10: The RELEASE file describes where and how the substance of interest is released. LON and LAT describe the exact location of the source, Z describes the height (in this case representing the height of the Chemours stack), ZKIND described above what media the source occurs, mass is set to 100 which can be manipulate to represent real scenarios and PART represents the number of particles released in the simulation.

RELEASE FILE	SETTINGS	EXPLANATION
NSPEC	2	Total number of species released
SPECIE NUM	12/13	Species numbers in directory
RELEASE 1		
IDATE1	20200101 / 20210601	Start date
ITIME1	090000	Start time
IDATE2	20201231 / 20211031	End date
ITIME2	090000	End time
LON1	4.727	Left longitude of release
LON2	4.727	Right longitude of release
LAT1	51.815	Lower latitude of release
LAT2	51.815	Upper latitude of release
Z1	25.90	Lower height of release (m)
Z2	25.90	Upper height of release (m)
ZKIND	1	Above ground
MASS 1	19.00, 0.00	Mass emitted (kg)
PART	3000000 / 1000000	Particles released (number) Large-scale / Local
COMMENT	RELEASE 1	Output File name
RELEASE 2		
IDATE1	20200101 / 20210601	Start date
ITIME1	090000	Start time
IDATE2	20201231 / 20211031	End date
ITIME2	090000	End time
LON1	4.724	Left longitude of release
LON2	4.724	Right longitude of release
LAT1	51.816	Lower latitude of release
LAT2	51.816	Upper latitude of release
Z1	25.90	Lower height of release (m)
Z2	25.90	Upper height of release (m)
ZKIND	1	Above ground
MASS 1	00.00, 81.00	Mass emitted (kg)
PART	3000000 / 1000000	Particles released (number) Large-scale / Local
COMMENT	RELEASE 2	Output File name

the deposition parameters sensitivity analysis in section 6, the PDENSITY and the PWEIGHTMOLECULAR inputs were based on HFPO-DA properties in Table 2 and PDQUER and PDSIGMA were based on the Particle mass and size distribution determination in section 2. Both the local and large-scale simulations shared identical Species files.

Table S2.11: SPECIES file containing information and properties of the aerosol release and HFPO-DA.

SPECIES FILE (12/13)	SETTINGS	EXPLANATION
PSPECIES	HFPO-DA	Name of chemical
PDEAY	off	Species half-life (degradation)
PCRAIN-AERO	1	Collection eff. rain
PCSNOW-AERO	1	Collection eff. snow
PCCN-AERO	0.9	Nucleation eff. rain
PIN-AERO	0.1	Nucleation eff. ice
PDENSITY	1850	Particle density (kg/m3)
PDQUER	Mode 1:1.2, Mode2:12	Particle diameter (µm)
PDSIGMA	Mode 1:3, Mode2:1.6	Standard deviation, spread
PWEIGHTMOLECULAR	330.5	Molar Weight (g/mol)

Table S2.12: Simulation time of each released particle. The AGECLASS file is set to 30 days and each particle will thus be followed for 30 days before it is removed and a new particle is released at the source[18].

AGECLASS FILE	SETTINGS	EXPLANATION
AGE	2592000	Lifetime of particles in seconds

S2.5 Emission evaluation

Once the local simulations had been conducted and the atmospheric measurements had been obtained, the two results could be compared. The local simulation assumed two potential emission scenarios, 95 kg/year and 3.2 kg/year, based on emission permits and abatement system insight according to Chemours Dordrecht's employee, (M. Reijmers, personal communication, February 2, 2022). The comparison between the model outputs and the real samples (Figue S2.11) suggest that the emissions decreased around June 22nd and that the mixed scenario, would be the most reasonable.

To investigate what part of the outputs reduced the correlation and what resulted in the best fit, the measurements and model outputs were split into three groups; one containing the June data (A-B), one containing the July - August data (C-D) and one containing the October data (E-F). These groups were plotted (see Figure S2.12) and the evaluation showed June to reduce the correlation (r = 0.51, p-value: $2.74 * 10^{-6} < 0.05$, R^2= 0.29), whereas the July - August and the October data showed good correlation and high significance (r = 0.87, p-value: $4.8 * 10^{-5} < 0.05$, R^2= 0.61) and (r = 0.85, p-value: $4 * 10^{-3} < 0.05$, R^2= 0.43) respectively.

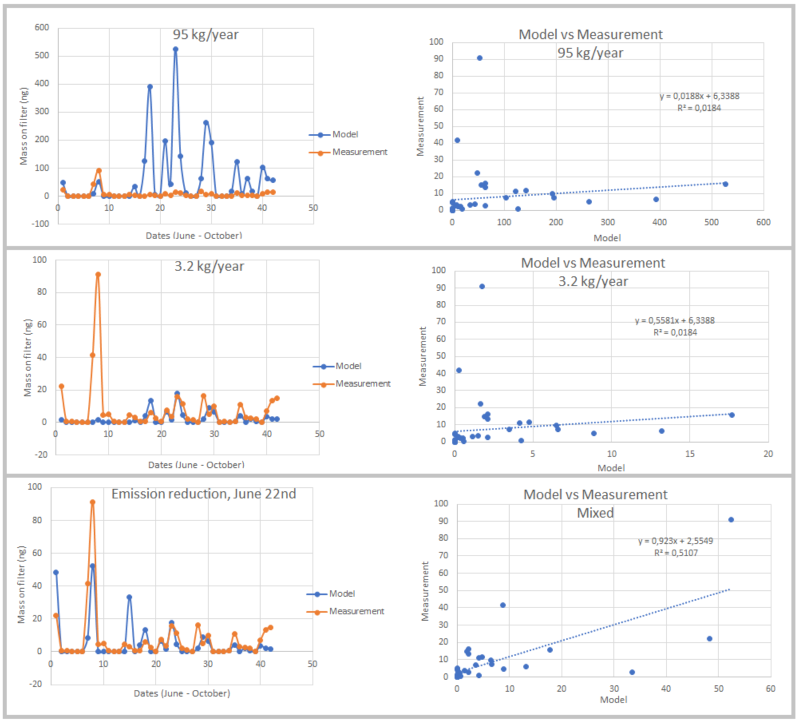

Figure S2.11: Two emission scenarios. The top two plots, show the 5-month period using the emission scenario of 95 kg/year. The two plots in the middle, shows the 5-month period using the 3,2 kg/year scenario and the bottom plot, which aligns the best, shows a mixed scenario, of 95/kg/year during June 1 – June 22, and then 3.2 kg/ year for the rest of the time.

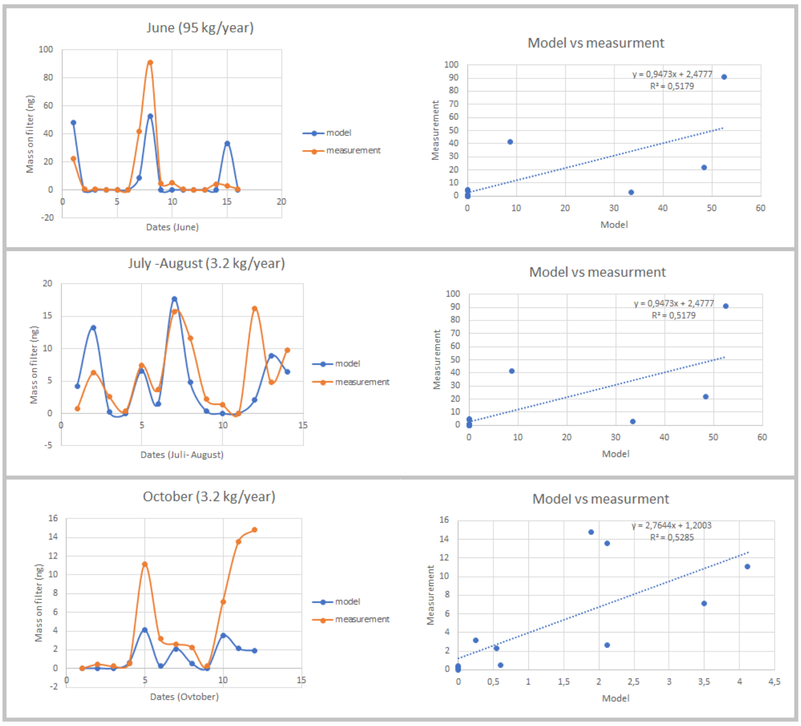

Figure S2.12: Model versus measurement comparison for the 3 different time periods, June, July -August and October , showing July-August and October to have high correlation and significance whereas June does not.

S2.6 Large-scale result

Two scenarios were used to assess the overall HFPO-DA deposition after a full year of emissions in 2020; a scenario depicted in SI-1 (450 kg/year) and the scenario depicted below (95 kg/year).

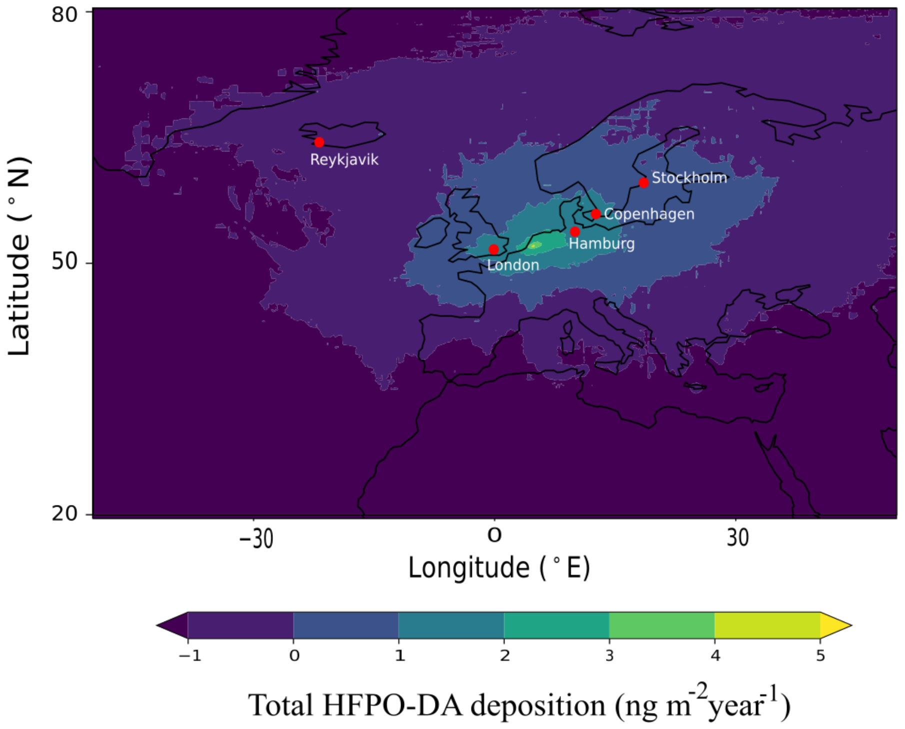

Figure S2.13: Scenario of the accumulated wet and dry HFPO-DA deposition after one full year (2020), with the Chemours facility in Dordrecht as the sole point source of the emissions, using the minimum HFPO-DA emission scenario of 95 kg/year. The red dots in the plots correspond to different cities in northern Europe while the scale represents the orders of magnitude with 0 = 1 ng m -2 year -1 .

References

[1] Jonathan L Barber, Urs Berger, Chakra Chaemfa, Sandra Huber, Annika Jahnke, Christian Temme, and Kevin C Jones. Analysis of per-and polyfluorinated alkyl substances in air samples from northwest europe. *Journal of environmental monitoring*, 9(6):530–541, 2007.

[2] Emma L D'Ambro, Havala OT Pye, Jesse O Bash, James Bowyer, Chris Allen, Christos Efstathiou, Robert C Gilliam, Lara Reynolds, Kevin Talgo, and Benjamin N Murphy. Characterizing the air emissions, transport, and deposition of per-and polyfluoroalkyl substances from a fluoropolymer manufacturing facility. *Environmental science & technology*, 55(2):862–870, 2021.

[3] ECHA. SVCH SUPPORT DOCUMENT, url: https://echa.europa.eu/documents/10162/53fa6a5b-e95f-3128-ea9d-fa27f43b18bc, year: 2019, acessed: 2021-12-03.

[4] S Eckhardt, A Stohl, H Sodemann, A Frank, P Seibert, and G Wotawa. The lagrangian particle dispersion model flexpart version 8.0. 2008.

[5] US EPA. Air quality criteria for particulate matter (vol one). 2004. URL `https://cfpub.epa.gov/ncea/risk/recordisplay.cfm?deid=87903`.

[6] Vermeulen Floor and Adril W Bom-Lemstra. Brief Zuid-Holland - Verdere verlaging PFAS-emissies Chemours naar lucht en water, published: December 2019, url: https://www.dcmr.nl/sites/default/files/2021-06/Kopie%20van%20Stukken-Chemours.pdf, acessed: 2022-02-03 but no longer available on website, Copies of the original material can be obtained from the corresponding author on request. .

[7] Vermeulen Floor and Adril W Bom-Lemstra. Brief Zuid-Holland - Reactie RIVM op EFSA-opinie PFAS, published: January 2021, url: https://www.dcmr.nl/sites/default/files/2021-06/Kopie%20van%20Stukken-Chemours.pdf, acessed: 2022-01-08 but no longer available on website, Copies of the original material can be obtained from the corresponding author on request. .

[8] Geosyntec. Site associated pfas fate and transport study chemours fayetteville works, url: https://www.chemours.de/-/media/files/corporate/p27-pfas-fate-transport-study-2019-06-24.pdf?rev=6a198d6bfae4429db853b01a52506e05&hash=AB025D2851C41580C53D3FE9ECEB8A93, acessed: 2022-09-05.

[9] Melissa Ines Gomis, Zhanyun Wang, Martin Scheringer, and Ian T Cousins. A modeling assessment of the physicochemical properties and environmental fate of emerging and novel per-and polyfluoroalkyl substances. *Science of the Total Environment*, 505:981–991, 2015.

[10] Kai-Uwe Goss. The p k a values of pfoa and other highly fluorinated carboxylic acids. *Environmental science & technology*, 42(2):456–458, 2008.

[11] Henrik Grythe, Nina I Kristiansen, Christine D Groot Zwaaftink, Sabine Eckhardt, Johan Ström, Peter Tunved, Radovan Krejci, and Andreas Stohl. A new aerosol wet removal scheme for the lagrangian particle model flexpart v10. *Geoscientific Model Development*, 10(4):1447–1466, 2017.

[12] Zachary R Hopkins, Mei Sun, Jamie C DeWitt, and Detlef RU Knappe. Recently detected drinking water contaminants: Genx and other per-and polyfluoroalkyl ether acids. *Journal-American Water Works Association*, 110(7):13–28, 2018.

[13] TSI Incorporated. Aerosol statistics lognormal distributions and dn/dlogdp, application note pr-001. URL `https://tsi.com/getmedia/1621329bf4104dce992be21e1584481a/PR-001RevA_AerosolStatisticsAppNote?ext=.pdf`.

[14] Vlassis A Karydis, Alexandra P Tsimpidi, Andrea Pozzer, and Jos Lelieveld. How alkaline compounds control atmospheric aerosol particle acidity. *Atmospheric Chemistry and Physics*, 21(19):14983–15001, 2021.

[15] Huiju Lin, Sachi Taniyasu, Eriko Yamazaki, Si Wei, Xinhong Wang, Nan Gai, Jin Hyo Kim, Heesoo Eun, Paul KS Lam, and Nobuyoshi Yamashita. Per-and polyfluoroalkyl substances in the air particles of asia: levels, seasonality, and size-dependent distribution. *Environmental Science & Technology*, 54(22):14182–14191, 2020.

[16] John R Murrell and Willard B Nixon. Determination of the dissociation constant and uv-vis absorption spectra of h-28307. *Easton, Maryland, USA, Wildlife International, Ltd*, page 44, 2008.

[17] David O'Hagan. Understanding organofluorine chemistry. an introduction to the c–f bond. *Chemical Society Reviews*, 37(2):308–319, 2008.

[18] Ignacio Pisso, Espen Sollum, Henrik Grythe, Nina I Kristiansen, Massimo Cassiani, Sabine Eckhardt, Delia Arnold, Don Morton, Rona L Thompson, Christine D Groot Zwaaftink, et al. The lagrangian particle dispersion model flexpart version 10.4. *Geoscientific Model Development*, 12 (12):4955–4997, 2019.

[19] A. Stohl, C. Forster, A. Frank, P. Seibert, and G. Wotawa. Technical note: The lagrangian particle dispersion model FLEXPART version 6.2. *Atmos. Chem. Phys.*, 5:2461–2474, 2005. doi: 10.5194/acp-5-2461-2005.

[20] Andreas Stohl, Gerhard Wotawa, Petra Seibert, and Helga Kromp-Kolb. Interpolation errors in wind fields as a function of spatial and temporal resolution and their impact on different types of kinematic trajectories. *Journal of Applied Meteorology and Climatology*, 34(10):2149–2165, 1995.

[21] Anne Tipka, Leopold Haimberger, and Petra Seibert. Flex_extract v7. 1.2–a software package to retrieve and prepare ecmwf data for use in flexpart. *Geoscientific Model Development*, 13 (11):5277–5310, 2020.

[22] Nadezda Zikova and Vladimir Zdimal. Precipitation scavenging of aerosol particles at a rural site in the czech republic. *Tellus B: Chemical and Physical Meteorology*, 68(1):27343, 2016.
